# Synthesis of ([1,2,4]triazolo[4,3-*a*]pyridin-3-ylmethyl)phosphonates and their benzo derivatives via 5-*exo*-*dig* cyclization

**DOI:** 10.3762/bjoc.15.159

**Published:** 2019-07-12

**Authors:** Aleksandr S Krylov, Artem A Petrosian, Julia L Piterskaya, Nataly I Svintsitskaya, Albina V Dogadina

**Affiliations:** 1 Department of Organic Chemistry, Saint-Petersburg State Institute of Technology, Saint Petersburg, 190013, Russia

**Keywords:** cyclization, fused-ring systems, nitrogen heterocycles, phosphorylation, rearrangement

## Abstract

A series of novel 3-methylphosphonylated [1,2,4]triazolo[4,3-*a*]pyridines was accessed through a 5-*exo*-*dig*-type cyclization of chloroethynylphosphonates and commercially available N-unsubstituted 2-hydrazinylpyridines. In addition, 3-methylphosphonylated [1,2,4]triazolo[4,3-*a*]quinolines and 1-methylphosphonylated [1,2,4]triazolo[3,4-*a*]isoquinolines were synthesized in a similar manner. The presence of a NO_2_ group in the starting hydrazinylpyridine induces a Dimroth-type rearrangement leading to 2-methylphosphonylated [1,2,4]triazolo[1,5-*a*]pyridines.

## Introduction

Due to the high polarization of the push–pull triple bond, haloacetylenes show high reactivity in nucleophilic substitution reactions. Our systematic studies of the reactions of chloroethynylphosphonates with various nucleophilic reagents have recently revealed a new direction of this reaction when using *C*,*N*-, *N*,*S*- and *N*,*N*-dinucleophiles. It is characterized by a selective 5-*endo*-*dig* cyclization to the corresponding five-membered rings. The obtained new compounds are of special interest due to the practical utility of the formed fused heterocycles, such as indoles [1], thiazolo[2,3-*b*][1,3,4]thiadiazole [2], and benzo[4,5]imidazo[2,1-*b*]thiazole [3], as well as due to the simultaneous presence of a biologically active phosphorus function in the molecules. Recently, we have shown that the reaction of chloroethynylphosphonates with 2-aminopyridines proceeds through a 5-*endo*-*dig* cyclization to form imidazo[1,2-*a*]pyridines [4]. In continuation of this study, herein we report an effective approach to the synthesis of new phosphonylated triazolopyridine derivatives by reacting chloroethynylphosphonates with 2-hydrazinylpyridines. The triazolopyridine ring is a structural fragment that is present in a number of drugs and [1,2,4]triazolo[4,3-*a*]pyridines were shown to have herbicidal [5–6], antifungal [7], neuroprotective [8–9] and antibacterial activity [10]. In addition, [1,2,4]triazolopyridine has been used as electron-acceptor unit in the synthesis of organic light emitting diodes (OLED) [11].

**Scheme 1:**
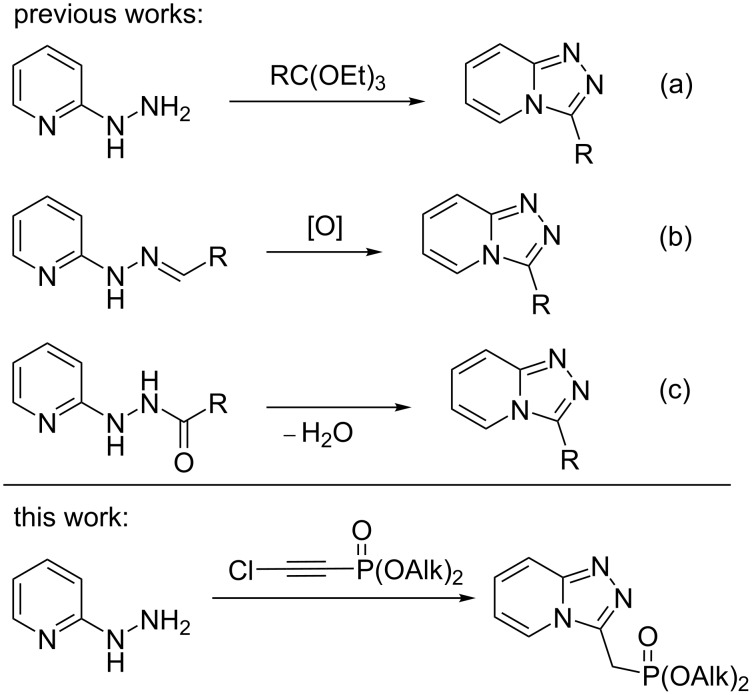
Synthetic approaches to [1,2,4]triazolo[4,3-*a*]pyridines.

2-Hydrazinylpyridines (a) and pyridinylhydrazones (b), as well as their acylated derivatives (c), are versatile scaffolds for the preparation of triazolopyridines (Scheme 1). The known methods for the [1,2,4]triazolopyridine ring formation use various condensation agents such as HCOOH, orthoesters, Lawesson’s reagent, hypervalent iodine reagents, etc.

The synthetic methods towards diverse [1,2,4]triazolo[3,4-*a*]pyridines have been reviewed in detail [12–13]. It should be noted that the use of acetylene species to create this heterocycle (including triazole ring) is presented only by few examples. There has been reported one method for the formation of a [1,2,4]triazolo[4,3-*a*]pyridine ring with participation of an acetylene triple bond; an oxidative cyclization during the reaction of terminal phenylacetylenes with 2-hydrazinylpyridines [14]. However, data on phosphorus-containing triazolopyridines are scarce, although the phosphoryl fragment widens the range of practical applications of such compounds. In this regard, Marchenko and co-workers [15] have reported the direct P(III)-phosphinylation of [1,2,4]triazolopyridines. The introduction of chlorethynylphosphonates in reactions with 2-hydrazinylpyridines allows to obtain methylphosphonylated triazolopyridine derivatives. Unlike our previous studies, in this case the reaction occurs through a 5-*exo*-*dig*-type cyclization.

## Results and Discussion

**Scheme 2:**
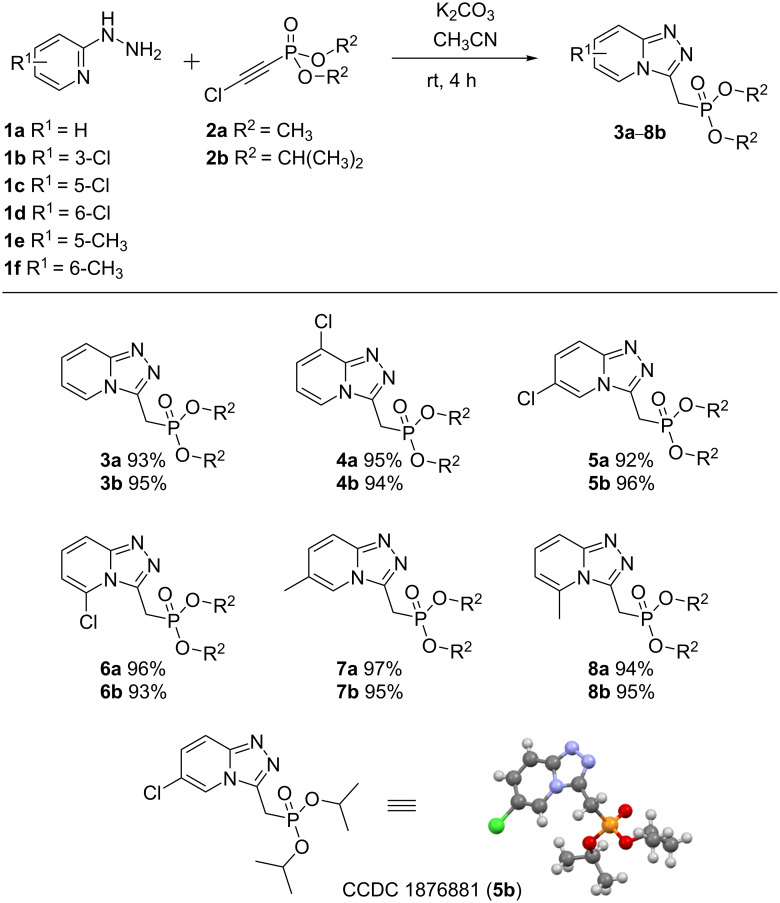
Synthesis of 3-methylphosphonylated [1,2,4]triazolo[4,3-*a*]pyridines*.* Reaction conditions: **1** (1 mmol), **2** (1 mmol), K_2_CO_3_ (1 mmol), CH_3_CN (5 mL), rt, 4 h.

The reactions of chloroethynylphosphonates with 2-hydrazinylpyridines were carried out in acetonitrile with an equimolar ratio of the starting reagents and anhydrous K_2_CO_3_ at room temperature. The reaction progress was monitored by ^31^P NMR spectroscopy. A complete conversion of chloroethynylphosphonate was achieved after 4 hours of reaction (Scheme 2). Note the reactions of 2-hydrazinylpyridines **1a**–**f** with chloroethynylphosphonates **2** afforded the title [1,2,4]triazolo[4,3-*a*]pyridines **3**–**8** in an almost quantitative yield. It should be emphasized that in the case of 2-hydrazinylpyridines the reaction selectivity is higher than in a similar reaction with 2-aminopyridines, where the formation of trace amounts of the corresponding amidines and amides was observed [4]. Moreover, the formazan-type products, the formation of which was observed in reactions of chloroethynylphosphonates with arylhydrazines, were not detected [16].

The structures of [1,2,4]triazolo[4,3-*a*]pyridines **3**–**8** were confirmed by IR, ^1^Н, ^13^C and ^31^P NMR spectroscopy. Chemical shifts of the phosphorus nucleus for compounds **3**–**8** were registered in the range of δ_P_ 22–23 and 18–19 ppm for dimethyl **а** and diisopropyl phosphonates **b**, respectively. In the ^1^Н NMR spectra of products **3**–**8**, the methylene unit was recorded as a doublet signal (δ_H_ 3.42–4.19 ppm) with a spin–spin coupling constant of ^2^*J*_НР_ ≈ 20 Hz. In the ^13^С NMR spectra, the methylene carbon resonated as a doublet at δ 23.5 ppm (*J*_CP_ = 143 Hz). In addition, the structures of triazolopyridines **3**–**8** were unambiguously confirmed by the crystal structure of **5b**.

**Scheme 3:**
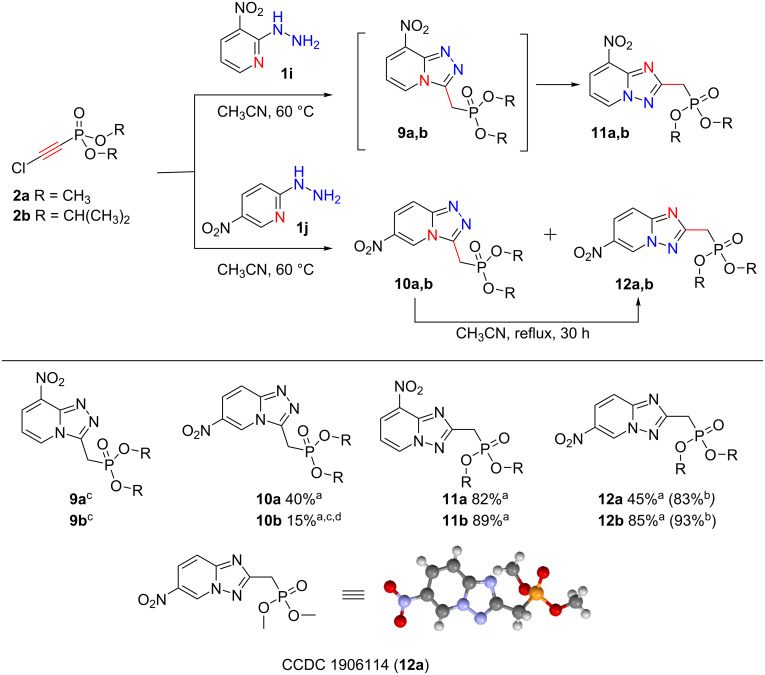
Synthesis of methylphosphonylated 6(8)-nitro-[1,2,4]triazolo[4,3-*a*]pyridines and 6(8)-nitro-[1,2,4]triazolo[1,5-*a*]pyridines. Conditions: ^a^**1** (1 mmol), **2** (1 mmol), CH_3_CN (5 mL), 60 °C. ^b^**1** (1 mmol), **2** (1 mmol), CH_3_CN (5 mL), reflux. ^c^The product was not isolated. ^d^The yield was determined by ^31^P NMR spectroscopy.

Remarkably, the presence of a nitro group in the pyridine ring of the starting 2-hydrazinylpyridines **1i** and **1j** significantly violates the reaction selectivity, leading to a rapid resinification of the reaction mixture. However, the exclusion of potassium carbonate allows the reaction to proceed selectively within 150 hours at a temperature of 60 °C. The reaction time could be reduced to 30 hours at reflux temperature. It should be noted that in this case the reaction led to the formation of 2 isomers one of which (**9**, **10**) is analogous to the [1,2,4]triazolo[4,3-*a*]pyridines **3**–**8** described above. The formation of another isomer **11**, **12**, is due to the Dimroth-type rearrangement, which is facilitated by the acceptor nitro group in the pyridine ring (Scheme 3) [17–18].

The reaction of 2-hydrazinyl-3-nitropyridine (**1i**) with dimethyl and diisopropyl chloroethynylphosphonates **2a** and **2b** proceeded selectively to furnish only [1,2,4]triazolo[1,5-*a*]pyridines **11**. The ease of the formation of isomers **11** in this case and **12** in other similar synthesis, is caused not only by the presence of a nitro group in the pyridine ring, but also by the presence of hydrogen chloride, which is eliminated by potassium carbonate in the former experiments (Scheme 2). The acid-promoted Dimroth rearrangement has been previously reported by Potts et al. [19].

**Scheme 4:**
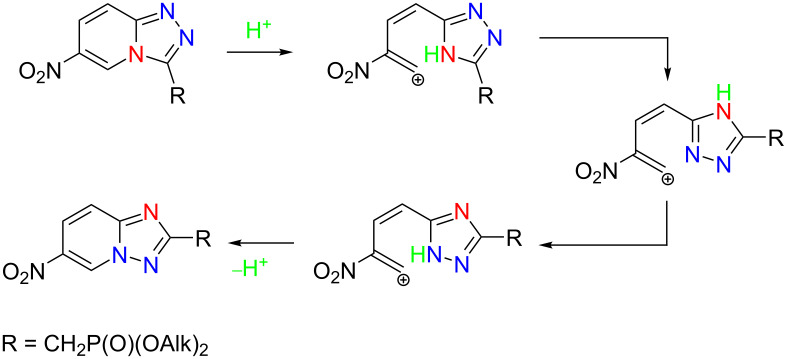
Acid-promoted Dimroth rearrangement pathway.

In contrast, the reaction of 2-hydrazinyl-5-nitropyridine (**1j**) with chloroethynylphosphonates at a temperature of 60 °C for 50 hours led to the formation of a mixture of isomers **10** and **12** in a ratio of ≈1:1. Boiling the reaction mixture after the complete conversion of chloroethynylphosphonate promotes a Dimroth-like rearrangement of [1,2,4]triazolo[4,3-*a*]pyridine **10** into [1,2,4]triazolo[1,5-*a*]pyridine **12** completely (Scheme 4).

Similar transformations have been observed in the reactions of 2-hydrazinylpyridines with ethyl imidates in the presence of 1.5 equiv of acetic acid [20]. When using highly electron-deficient 2-hydrazinopyridines, the [1,2,4]triazolo[4,3-*a*]pyridines obtained were converted into [1,2,4]triazolo[1,5-*a*]pyridines.

The structures of triazolopyridines **10**–**12** were confirmed by IR, ^1^H, ^13^C, and ^31^P NMR spectroscopy, high-resolution mass spectrometry, as well as single crystal X-ray diffraction.

**Scheme 5:**
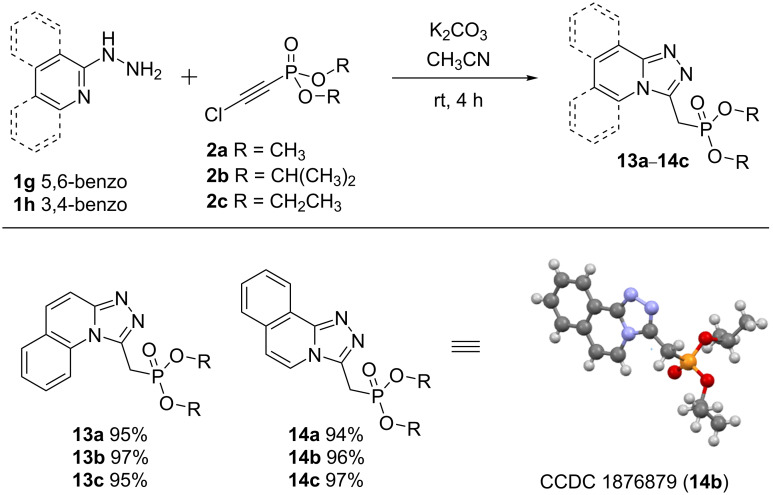
Synthesis of phosphonylated [1,2,4]triazolo[4,3-*a*]quinolines and [1,2,4]triazolo[3,4-*a*]isoquinolines. Reaction conditions: **1** (1 mmol), **2** (1 mmol), K_2_CO_3_ (1 mmol) CH_3_CN (5 mL), rt.

Next, under the conditions applied for the preparation of compounds **3**–**8**, the reaction of chloroethynylphosphonates with 2-hydrazinylquinoline (**1g**) and 1-hydrazinylisoquinoline (**1h**) resulted in the formation of [1,2,4]triazolo[4,3-*a*]quinolines **13** and [1,2,4]triazolo[3,4-*a*]isoquinolines **14**, respectively (Scheme 5).

In the ^31^P NMR spectra the chemical shifts of the phosphorus nuclei in **13** and **14** are observed in the 18.40–22.75 ppm region. The ^1^H NMR spectra contain characteristic doublet signals of the methylene group in the phosphoryl unit resonating at 3.75–4.19 ppm with ^2^*J*_HP_ = 20 Hz. At lower field, the signals of 6 protons of the (iso)quinoline rings are present at 7.1–8.7 ppm. In the ^13^C NMR spectra, the methylene and methine carbons of the triazole ring resonate as doublet signals with characteristic constants of spin–spin interaction with the phosphorus nucleus at 23.43–28.99 ppm (^1^*J*_СР_ = 143 Hz) and 53.5–72.1 ppm (^2^*J*_CP_ = 7 Hz), respectively. In addition, the structure of phosphonate **14b** was unambiguously proved by single crystal X-ray diffraction analysis.

**Scheme 6:**
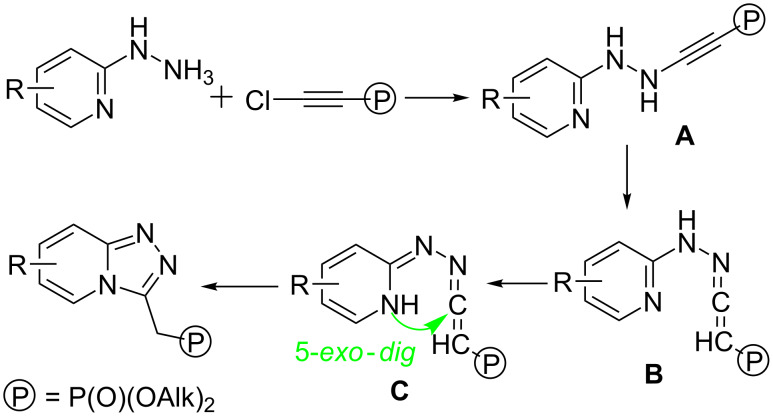
Plausible reaction pathway.

Probably, the reaction proceeds through the nucleophilic substitution of chlorine in the chloroethynylphosphonate to form ynamine intermediate **A**, isomerization of which provides ketenimine **B**. Further formation of the imine tautomer **C** enables an intramolecular 5-*exo*-*dig* cyclization to furnish the title [1,2,4]triazolo[4,3-*a*]pyridines (Scheme 6).

## Conclusion

In conclusion, a series of new 3-methylphosphonylated [1,2,4]triazolo[4,3-*a*]pyridines, [1,2,4]triazolo[3,4-*a*]isoquinolines and 1-methylphosphonylated [1,2,4]triazolo[4,3-*a*]quinolines were synthesized through a catalyst-free 5-*exo*-*dig*-type cyclization of chloroethynylphosphonates and commercially available N-unsubstituted 2-hydrazinylpyridines and 2(1)-hydrazinyl(iso)quinolines. Due to the presence of the fused [1,2,4]triazole hetaryl pharmacophore fragment and a phosphoryl group in the obtained compounds they are of great interest as promising substances with potential biological activity.

## Supporting Information

File 1 Experimental procedures, characterization data, and copies of ^1^H, ^13^C, and ^31^P NMR spectra for obtained compounds.

File 2 Crystallographic data for compound **5b** (CCDC 1876881).

File 3 Crystallographic data for compound **12a** (CCDC 1906114).

File 4 Crystallographic data for compound **14b** (CCDC 1876879).

## References

[1] Egorova A V, Viktorov N B, Starova G L, Svintsitskaya N I, Garabadziu A V, Dogadina A V (2017). Tetrahedron Lett.

[2] Egorov D M, Piterskaya Y L, Dogadina A V, Svintsitskaya N I (2015). Tetrahedron Lett.

[3] Egorov D M, Piterskaya Y L, Kartsev D D, Polukeev V A, Krivchun M N, Dogadina A V (2018). Russ J Gen Chem.

[4] Krylov A S, Kaskevich K I, Erkhitueva E B, Svintsitskaya N I, Dogadina A V (2018). Tetrahedron Lett.

[5] Liu X-H, Xu X-Y, Tan C-X, Weng J-Q, Xin J-H, Chen J (2015). Pest Manage Sci.

[6] Liu X-H, Zhai Z-W, Xu X-Y, Yang M-Y, Sun Z-H, Weng J-Q, Tan C-X, Chen J (2015). Bioorg Med Chem Lett.

[7] Liu X-H, Sun Z-H, Yang M-Y, Tan C-X, Weng J-Q, Zhang Y-G, Ma Y (2014). Chem Biol Drug Des.

[8] Ye Q, Mao W, Zhou Y, Xu L, Li Q, Gao Y, Wang J, Li C, Xu Y, Xu Y (2015). Bioorg Med Chem.

[9] Chun K, Park J-S, Lee H-C, Kim Y-H, Ye I-H, Kim K-J, Ku I-W, Noh M-Y, Cho G-W, Kim H (2013). Bioorg Med Chem Lett.

[10] Sadana A K, Mirza Y, Aneja K R, Prakash O (2003). Eur J Med Chem.

[11] Vadagaonkar K S, Yang C-J, Zeng W-H, Chen J-H, Patil B N, Chetti P, Chen L-Y, Chaskar A C (2019). Dyes Pigm.

[12] Shen Z-H, Wang Q, Yang M-Y, Sun Z-H, Weng J-Q, Tan C-X, Wu H-K, Han L, Liu X-H (2017). Curr Org Chem.

[13] Jones G, Sliskovic D R, Katritzky A R (1983). The chemistry of the triazolopyridines. Advances in Heterocyclic Chemistry.

[14] Reddy L M, Reddy V V, Prathima P S, Reddy C K, Reddy B V S (2018). Adv Synth Catal.

[15] Marchenko A P, Koidan H N, Kirilchuk A A, Rozhenko A B, Yurchenko A A, Kostyuk A N (2015). Heteroat Chem.

[16] Lyamenkova D V, Viktorov N B, Ponyaev A I, Dogadina A V (2014). Russ J Gen Chem.

[17] Bouteau B, Lancelot J-C, Robba M (1990). J Heterocycl Chem.

[18] Kost A N, Gromov S P, Sagitullin R S (1981). Tetrahedron.

[19] Potts K T, Surapaneni C R (1970). J Heterocycl Chem.

[20] Schmidt M A, Qian X (2013). Tetrahedron Lett.

