# Supplementary material for: Synthesis of ([1,2,4]triazolo[4,3-a]pyridin-3-ylmethyl)phosphonates and their benzo derivatives via 5-exo-dig cyclization
Source: Beilstein J Org Chem. 2019 Jul 12;15:1563–8. doi: 10.3762/bjoc.15.159 (PMC6633597; doi:10.3762/bjoc.15.159)

BEILSTEIN JOURNAL OF ORGANIC CHEMISTRY

Supporting Information

for

Synthesis of ([1,2,4]triazolo[4,3-*a*]pyridin-3-ylmethyl)phosphonates and their benzo derivatives via 5-*exo*-*dig* cyclization

Aleksandr S. Krylov, Artem A. Petrosian, Julia L. Piterskaya, Nataly I. Svintsitskaya and Albina V. Dogadina

Beilstein J. Org. Chem. **2019,** *15,* 1563–1568. doi:10.3762/bjoc.15.159

Experimental procedures, characterization data, and copies of ^{1}H, ^{13}C, and ^{31}P NMR spectra for obtained compounds

License and Terms: This is a supporting information file under the terms of the Creative Commons Attribution License (http://creativecommons.org/licenses/by/4.0). Please note that the reuse, redistribution and reproduction in particular requires that the authors and source are credited.
The license is subject to the *Beilstein Journal of Organic Chemistry* terms and conditions: (https://www.beilstein-journals.org/bjoc)

List of Contents

A. General Methods S2
B. General Procedures S2
C. References S2
D. Analytical Data S2
E. NMR Spectra S10

A. General Methods

All reactions were carried out under an inert atmosphere of argon in oven dried glassware with magnetic stirring, unless otherwise noted. All chemicals were purchased from were obtained from commercial suppliers and used without further purification. Column chromatography purifications were performed using Merck silica gel 60. Commercial grade solvents and reagents were used without further purification. Analytical thin layer chromatography (TLC) was performed using Merck 60 F254 silica gel plate. Subsequent to elution, plates were visualized using UV radiation (254 nm). Melting points were uncorrected and were recorded on a Kofler hot-stage (VEB Wägetechnik Rapido, PHMK 81/2969).^{1}H, ^{13}C, ^{15}N and ^{31}P NMR spectra were registered at 400.13, 100.61, 41.50 and 161.98 MHz, respectively, using a Bruker Avance 400 spectrometer. Residual solvent peaks were used as reference. IR spectra were registered on an IRPrestige-21 (Shimadzu) instrument from KBr pellets. HRMS (ESI) analysis was performed on a BrukermicrOTOF mass spectrometer. The X-ray crystal structures were detected on single crystal diffractometers Agilent Technologies (Oxford Diffraction) «Supernova» and «Xcalibur».

B. Typical experimental procedure for the synthesis of dialkyl 2-chloroethynylphosphonates.[1,2]

In a 250 ml round-bottomed flask equipped with an efficient stirrer, thermometer, and reflux condenser a mixture of sodium hydroxide (3 mol), water (150 ml) and benzyltriethylammonium chloride (2 g) was heated with stirring on an oil bath up to 120 ºC. Then, 10 ml of diethyl ether and a solution of trichloroethylene (1 mol) in 100 ml of diethyl ether were added dropwise. Simultaneously the reaction product was distilled off. The distillate consists mainly of the solution of dichloroacetylene in ether. The distillate was dried over calcium chloride, and then over sodium metal. Yield 60%. Trialkylphosphite (0.6 mol) was added dropwise with stirring at 5–20 °C to ether solution of dichloroacetylene obtained. The reaction mixture was kept overnight. Then, diethyl ether was distilled off. The residue was distilled in vacuum (1 mm Hg), collecting the fraction bp 52–60 °C. Yield 68–72%.

Typical experimental procedure for the synthesis of compounds 3-8, 13 and 14. The corresponding 2-hydrazinylpyridines **1a**–**f,g,h** (1 mmol) was added to a mixture of dialkyl chloroacetylenephosphonate **2a**–**c** (1 mmol) and K_2CO_3 (1 mmol) in 5 mL of anhydrous acetonitrile at room temperature. The mixture was stirred at room temperature until the starting chloroethynylphosphonate consumed (monitoring with ^{31}P NMR). After the reaction was completed, the reaction mixture was filtered and evaporated. The oily residue was chromatographed on SiO_2 eluting with 1% MeOH in $CHCl_3$.

Typical experimental procedure for the synthesis of compounds 10-12. The corresponding 2-hydrazinylpyridines **1i,j** (1 mmol) was added to a mixture of dialkyl chloroacetylenephosphonate **2a,b** (1 mmol) in 5 mL of anhydrous acetonitrile at room temperature. The mixture was stirred (at 60°C for **10**; at reflux for **11** and **12**) until the starting chloroethynylphosphonate consumed (monitoring with ^{31}P NMR). After the reaction was completed, the reaction mixture was filtered and evaporated. The oily residue was chromatographed on SiO_2 eluting with 1% MeOH in $CHCl_3$.

C. References

1. Ionin, B. I.;Petrov, A. A. *Zh. Obshch. Khim.* **1965**, *35*, 1917–1921.
2. Pielichowski, J.; Popielarz, R. *Synthesis (Stuttg).* **1984**, *05*, 433–434.

D. Analytical Data

Dimethyl ([1,2,4]triazolo[4,3-a]pyridin-3-ylmethyl)phosphonate 3a

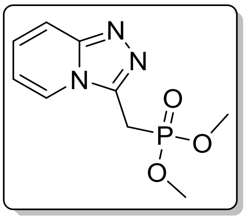

Following the general procedure, **3a** was obtained from **1a** and **2a** as pale yellow oil. ^{1}H NMR (400 MHz, $CDCl_3$) δ 8.23 (d, *J* = 7.0 Hz, 1H, 5-$H_{ar.}$), 7.72 (d, *J* = 9.3 Hz, 1H, 8-$H_{ar.}$), 7.39 – 7.17 (m, 1H, 7-$H_{ar.}$), 6.87 (m, 1H, 6-$H_{ar.}$), 3.78 (d, $^2J_{HP}$ = 21.5 Hz, 2H, CH_2), 3.73 (d, $^3J_{HP}$ = 11.1 Hz, 6H, 2CH_3). ^{13}C NMR (101 MHz, $CDCl_3$) δ 150.5, 138.8 (d, $^2J_{CP}$ = 10.0 Hz), 127.2, 123.2, 116.3, 113.8, 53.4 (d, $^2J_{CP}$ = 6.8 Hz), 23.4 (d, $^1J_{CP}$ = 143.0 Hz).^{31}P NMR (162 MHz, $CDCl_3$) δ 22.85. ν_{max}(KBr)/cm^{-1} 1433, 2951, 1637, 1510, 1388, 1251, 1238, 1188, 1043, 1012, 871, 814, 738. HRMS-ESI (m/z): calcd for $C_9H_{12}N_3O_3P$, $[M+H]^+$: 242.0689, found 242.0694.

Diisopropyl ([1,2,4]triazolo[4,3-a]pyridin-3-ylmethyl)phosphonate 3b

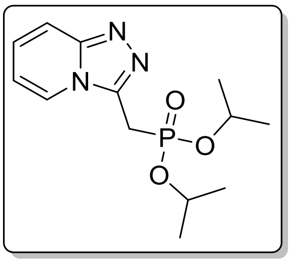

Following the general procedure, **3b** was obtained from **1a** and **2b** as pale yellow oil. ^{1}H NMR (400 MHz, $CDCl_3$) δ 8.05 (d,$^3J_{HH}$ = 9.3 Hz, 1H, 5-$H_{ar.}$), 7.38 (d,$^3J_{HH}$ = 9.3 Hz, 1H, 8-$H_{ar.}$), 7.03 – 6.88 (m, 1H, 7-$H_{ar.}$), 6.56 (m, 1H, 6-$H_{ar.}$), 4.34 (m, $^3J_{HP}$ = 12.6 Hz, $^3J_{HH}$ = 6.2 Hz, 2H, 2CH), 3.47 (d,$^3J_{HP}$ = 20.3 Hz, 2H, CH_2), 0.96 (d, $^3J_{HH}$ = 6.2 Hz, 6H, 2CH_3), 0.92 (d, $^3J_{HH}$ = 6.2 Hz, 6H, 2CH_3).^{13}C NMR (101 MHz, $CDCl_3$) δ 150.1, 139.2 (d, $^2J_{CP}$ = 9.9 Hz), 126.9, 123.6, 115.6, 113.1, 71.6 (d, $^2J_{CP}$ = 6.9 Hz), 25.1 (d, $^1J_{CP}$ = 143.3 Hz), 23.7 (d, $^3J_{CP}$ = 3.9 Hz), 23.5 (d, $^3J_{CP}$ = 4.9 Hz). ^{31}P NMR (162 MHz, $CDCl_3$) δ 18.40. ν_{max}(KBr)/cm^{-1} 3461, 2981, 1637, 1507, 1387, 1376, 1251, 1105, 988, 759. HRMS-ESI (m/z): calcd for $C_{13}H_{20}N_3O_3P$, $[M+H]^+$: 298.1315, found 298.1301.

Dimethyl ((8-chloro-[1,2,4]triazolo[4,3-a]pyridin-3-yl)methyl)phosphonate 4a

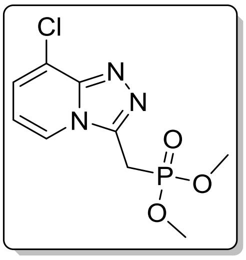

Following the general procedure, **4a** was obtained from **1b** and **2a** as pale solid. ^{1}H NMR (400 MHz, $CDCl_3$) δ 8.24 (d, $^3J_{HH}$ = 7.0 Hz, 1H, 5-$H_{ar.}$), 7.34 (d, $^3J_{HH}$ = 7.1 Hz, 1H, 7-$H_{ar.}$), 6.85 (t, $^3J_{HH}$ = 7.0 Hz, 1H, 6-$H_{ar.}$), 3.81 (d, *J* = 20.6 Hz, 2H, CH_2), 3.76 (d, $^3J_{HP}$ = 11.0 Hz, 6H, 2OCH_3). ^{13}C NMR (101 MHz, $CDCl_3$) δ 148.7, 140.5 (d, $^2J_{CP}$ = 10.1 Hz), 126.1, 122.5, 122.1, 113.7, 53.5 (d, $^2J_{CP}$ = 6.7 Hz), 23.7 (d, $^1J_{CP}$ = 142.6 Hz).^{31}P NMR (162 MHz, $CDCl_3$) δ 22.33. ν_{max}(KBr)/cm^{-1} 3434, 2952, 1630, 1506, 1383, 1321, 1263, 1239, 1041, 1026, 1009, 945, 865, 818. HRMS-ESI (m/z): calcd for $C_9H_{11}ClN_3O_3P$, $[M+Na]^+$: 298.0119, found 298.0132. M. p. 155 - 160 °C.

Diisopropyl ((8-chloro-[1,2,4]triazolo[4,3-a]pyridin-3-yl)methyl)phosphonate 4b

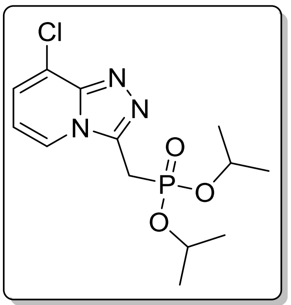

Following the general procedure, **4b** was obtained from **1b** and **2b** as pale yellow oil. ^{1}H NMR (400 MHz, $CDCl_3$) δ 8.13 (d, $^3J_{HH}$ = 6.9 Hz, 1H, 5-$H_{ar.}$), 7.14 (d, $^3J_{HH}$ = 7.1 Hz, 1H, 7-$H_{ar.}$), 6.66 (t, $^3J_{HH}$ = 7.0 Hz, 1H, 6-$H_{ar.}$), 4.44 (m, 2H, 2CH), 3.59 (d, $^2J_{HP}$ = 20.3 Hz, 2H, CH_2), 1.08 (d, $^3J_{HH}$ = 6.1 Hz, 6H, 2CH_3), 1.04 (d,

$^{3}J_{HH}$ = 6.2 Hz, 6H, $2CH_3$). ^{13}C NMR (101 MHz, $CDCl_3$) δ 148.2, 141.1 (d, $^{2}J_{CP}$ = 9.9 Hz), 126.1, 122.7, 121.6, 113.2, 72.0 (d, $^{2}J_{CP}$ = 7.0 Hz), 25.3 (d, $^{1}J_{CP}$ = 143.1 Hz), 23.8 (d, $^{3}J_{CP}$ = 4.0 Hz), 23.7 (d, $^{3}J_{CP}$ = 4.9 Hz). ^{31}P NMR (162 MHz, $CDCl_3$) δ 18.08. ν_{max}(KBr)/cm^{-1} 3464, 2981, 1632, 1502, 1385, ,1249, 1104, 990, 739. HRMS-ESI (m/z): calcd for $C_{13}H_{19}ClN_3O_3P$, $[M+H]^+$: 332.0925, found 332.0925.

Dimethyl ((6-chloro-[1,2,4]triazolo[4,3-a]pyridin-3-yl)methyl)phosphonate 5a

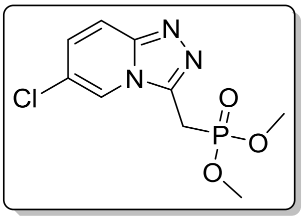

Following the general procedure, **5a** was obtained from **1c** and **2a** as pale solid. ^{1}H NMR (400 MHz, $CDCl_3$) δ 8.32 (s, 1H, 5-$H_{ar.}$), 7.70 (d, $^{3}J_{HH}$ = 9.7 Hz, 1H, 8-$H_{ar.}$), 7.22 (dd, $^{3}J_{HH}$ = 9.7,$^{4}J_{HH}$= 1.6 Hz, 1H, 7-$H_{ar.}$), 3.76 (d, $^{3}J_{HP}$ = 11.0 Hz, 6H, $2OCH_3$),3.75 (d, $^{2}J_{HP}$ = 20.6 Hz, 2H, CH_2). ^{13}C NMR (101 MHz, $CDCl_3$) δ 149.3, 139.2 (d, $^{2}J_{CP}$ = 9.9 Hz), 129.2, 122.7, 121.2, 116.9, 53.6 (d, $^{3}J_{CP}$ = 6.7 Hz), 23.6 (d, $^{1}J_{CP}$ = 143.0 Hz).^{31}P NMR (162 MHz, $CDCl_3$) δ 22.45. ν_{max}(KBr)/cm^{-1} 3087, 2950, 1635, 1506, 1255, 1239, 1043, 1024, 874, 820. HRMS-ESI (m/z): calcd for $C_9H_{11}ClN_3O_3P$, $[M+H]^+$: 276.0299, found 276.0308. M. p. 144 - 145 °C.

Diisopropyl ((6-chloro-[1,2,4]triazolo[4,3-a]pyridin-3-yl)methyl)phosphonate 5b

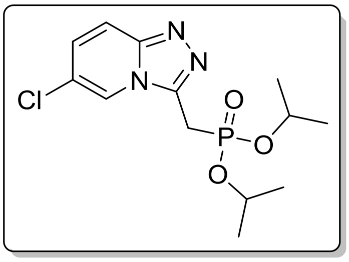

Following the general procedure, **5b** was obtained from **1c** and **2b** as pale solid. ^{1}H NMR (400 MHz, $CDCl_3$) δ 8.37 (s, 1H, 5-$H_{ar.}$), 7.67 (d, $^{3}J_{HH}$ = 9.7 Hz, 1H, 8-$H_{ar.}$), 7.19 (dd, $^{3}J_{HH}$ = 9.7,$^{4}J_{HH}$ 1.8 Hz, 1H, 7-$H_{ar.}$), 4.65 (m, 2H, 2CH), 3.69 (d, $^{2}J_{HP}$ = 20.3 Hz, 2H, CH_2), 1.26 (d, $^{3}J_{HH}$ = 6.1 Hz, 6H, $2CH_3$), 1.25 (d, $^{3}J_{HH}$ = 6.1 Hz, 6H, $2CH_3$). ^{13}C NMR (101 MHz, $CDCl_3$) δ 149.1, 139.8 (d, $^{2}J_{CP}$ = 10.2 Hz), 128.9, 122.1, 121.66, 116.8, 72.4 (d, $^{2}J_{CP}$ = 7.2 Hz), 25.7 (d, $^{1}J_{CP}$ = 143.7 Hz), 24.1 (d, $^{3}J_{CP}$ = 4.2 Hz), 24.0 (d, $^{3}J_{CP}$ = 4.7 Hz). ^{31}P NMR (162 MHz, $CDCl_3$) δ 18.12. ν_{max}(KBr)/cm^{-1} 3075, 2980, 2921, 1634, 1506, 1384, 1238, 1025, 982, 802, 796. HRMS-ESI (m/z): calcd for $C_{13}H_{19}ClN_3O_3P$, $[M+H]^+$: 332.0925, found 332.0929. M. p. 132 - 133 °C.

Dimethyl ((5-chloro-[1,2,4]triazolo[4,3-a]pyridin-3-yl)methyl)phosphonate 6a

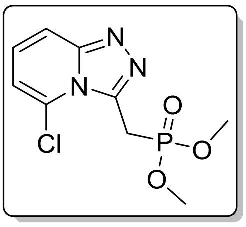

Following the general procedure, **6a** was obtained from **1d** and **2a** as pale solid. ^{1}H NMR (400 MHz, $CDCl_3$) δ 8.24 (d, $^{3}J_{HH}$ = 7.0 Hz, 1H, 8-$H_{ar.}$), 7.34 (d, $^{3}J_{HH}$ = 7.1 Hz, 1H, 7-$H_{ar.}$), 6.85 (t, $^{3}J_{HH}$ = 7.0 Hz, 1H, 6-$H_{ar.}$), 4.19 (d, J = 20.6 Hz, 2H, CH_2), 3.76 (d, $^{3}J_{HP}$ = 11.0 Hz, 6H, $2OCH_3$). ^{13}C NMR (101 MHz, $CDCl_3$) δ 148.7, 140.5 (d, $^{2}J_{CP}$ = 10.1 Hz), 126.1, 122.5, 122.1, 113.7, 53.5 (d, $^{2}J_{CP}$ = 6.7 Hz), 23.7 (d, $^{1}J_{CP}$ = 142.6 Hz).^{31}P NMR (162 MHz, $CDCl_3$) δ 22.33. ν_{max}(KBr)/cm^{-1} 2957, 2360, 1630, 1494, 1404, 1354, 1298, 1267, 1248, 1230, 1138, 1003, 875, 829, 783, 731, 669, 597, 524. HRMS-ESI (m/z): calcd for $C_9H_{11}ClN_3O_3P$, $[M+H]^+$: 276.0299, found 276.0293. M. p. 99 - 100 °C.

Diisopropyl ((5-chloro-[1,2,4]triazolo[4,3-a]pyridin-3-yl)methyl)phosphonate 6b

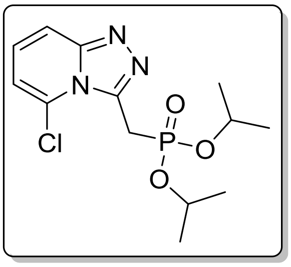

Following the general procedure, **3b** was obtained from **1d** and **2b** as pale yellow oil. ^{1}H NMR (400 MHz, $CDCl_3$) δ 7.64 (dd, $^{3}J_{HH}$ = 9.2,$^{4}J_{HH}$= 0.9 Hz, 1H, 8-$H_{ar.}$), 7.14 (dd, $^{3}J_{HH}$ = 9.2, 7.0 Hz, 1H, 7-$H_{ar.}$), 6.82 (dd, $^{3}J_{HH}$ = 7.0,$^{4}J_{HH}$= 0.8 Hz, 1H, 6-$H_{ar.}$), 4.66 (m, 2H, 2CH), 4.11 (d, $^{2}J_{HP}$ = 20.7 Hz, 2H, CH_2), 1.26 (d, $^{3}J_{HH}$ = 6.2 Hz, 6H), 1.23 (d, $^{3}J_{HH}$ = 6.2 Hz, 6H). ^{13}C NMR (101 MHz, $CDCl_3$) δ 152.0, 140.4 (d, $^{2}J_{CP}$ = 11.0 Hz), 127.5, 125.4, 115.2, 115.0, 71.5 (d, $^{2}J_{CP}$ = 7.2 Hz), 27.9 (d, $^{1}J_{CP}$ = 142.9 Hz), 23.8 (d, $^{3}J_{CP}$ = 3.7 Hz), 23.7 (d, $^{3}J_{CP}$ = 5.2 Hz). ^{31}P NMR (162 MHz, $CDCl_3$) δ 18.20. ν_{max}(KBr)/cm^{-1} 3471, 2981, 1629, 1496, 1387, 1255, 1106, 987, 788. HRMS-ESI (m/z): calcd for $C_{13}H_{19}ClN_3O_3P$, $[M+H]^+$: 332.0925, found 332.0919.

Dimethyl ((6-methyl-[1,2,4]triazolo[4,3-a]pyridin-3-yl)methyl)phosphonate 7a

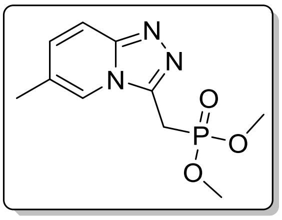

Following the general procedure, 7**a** was obtained from **1e** and **2a** as pale solid. ^{1}H NMR (400 MHz, $CDCl_3$) δ 8.01 (d, $^{4}J_{HH}$ = 1.4 Hz, 1H, 5-$H_{ar.}$), 7.67 (d, $^{3}J_{HH}$ = 9.4 Hz, 1H, 8-$H_{ar.}$), 7.14 (dd, $^{3}J_{HH}$ = 9.4,$^{4}J_{HH}$= 1.4 Hz, 1H, 7-$H_{ar.}$), 3.77 (d, $^{3}J_{HP}$ = 11.1 Hz, 6H, 2CH_3), 3.77 (d, $^{2}J_{HP}$ = 20.3 Hz, 2H, CH_2), 2.38 (s, 3H, CH_3). ^{13}C NMR (101 MHz, $CDCl_3$) δ 150.0, 138.2 (d, $^{2}J_{CP}$ = 9.8 Hz), 130.7, 123.9, 120.2, 115.6, 53.4 (d, $^{2}J_{CP}$ = 6.8 Hz), 23.4 (d, $^{1}J_{CP}$ = 143.2 Hz), 18.3. ^{31}P NMR (162 MHz, $CDCl_3$) δ 22.92. ν_{max}(KBr)/cm^{-1} 3396, 2947, 1649, 1520, 1425, 1256, 1187, 1042, 1020, 876, 822, 759, 773. HRMS-ESI (m/z): calcd for $C_{10}H_{14}N_3O_3P$, $[M+Na]^+$: 278.0665, found 278.0666. M. p. 128 - 130 °C.

Diisopropyl ((6-methyl-[1,2,4]triazolo[4,3-a]pyridin-3-yl)methyl)phosphonate 7b

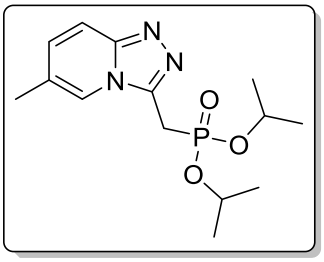

Following the general procedure, **7b** was obtained from **1e** and **2b** as pale yellow oil. ^{1}H NMR (400 MHz, $CDCl_3$) δ 7.79 (s, 1H, 5-$H_{ar.}$), 7.27 (d,$^{3}J_{HH}$ = 9.4 Hz, 1H, 8-$H_{ar.}$), 6.80 (d, $^{3}J_{HH}$ = 9.4 Hz, 1H, 7-$H_{ar.}$), 4.34 (m, $^{3}J_{HH}$ = 6.2 Hz, 2H, 2CH), 3.42 (d, $^{2}J_{HP}$ = 20.3 Hz, 2H, CH_2), 2.02 (s, 3H, CH_3) 0.96 (d, $^{3}J_{HH}$ = 6.2 Hz, 6H, 2CH_3), 0.92 (d, $^{3}J_{HH}$ = 6.2 Hz, 6H, 2CH_3). ^{13}C NMR (101 MHz, $CDCl_3$) δ 149.4, 138.6 (d, $^{2}J_{CP}$ = 10.1 Hz), 130.3, 122.9, 120.6, 114.9, 71.6 (d, $^{2}J_{CP}$ = 7.0 Hz), 25.1 (d, $^{1}J_{CP}$ = 143.6 Hz), 23.7 (d, $^{3}J_{CP}$ = 3.9 Hz), 23.5 (d, $^{3}J_{CP}$ = 5.0 Hz), 17.9. ^{31}P NMR (162 MHz, $CDCl_3$) δ 18.51. ν_{max}(KBr)/cm^{-1} 3494, 2981, 1646, 1525, 1383, 1274, 1209, 1105, 999, 813. HRMS-ESI (m/z): calcd for $C_{14}H_{22}N_3O_3P$, $[M+H]^+$: 312.1472, found 312.1466.

Dimethyl ((5-methyl-[1,2,4]triazolo[4,3-a]pyridin-3-yl)methyl)phosphonate 8a

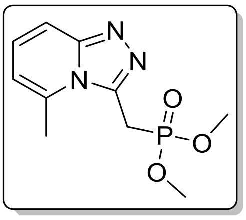

Following the general procedure, **8a** was obtained from **1f** and **2a** as pale yellow oil. ^{1}H NMR (400 MHz, $CDCl_3$) δ 7.09 (d, $^3J_{HH}$ = 9.2 Hz, 1H, 8-H$_{ar.}$), 6.70 (dd,$^3J_{HH}$ = 9.2, 6.7 Hz, 1H, 7-H$_{ar.}$), 6.14 (d, $^3J_{HH}$ = 6.7 Hz, 1H, 6-H$_{ar.}$), 3.61 (d, $^2J_{HP}$ = 20.6 Hz, 2H, CH_2), 3.35 (d, $^3J_{HP}$ = 11.1 Hz, 6H, $2CH_3$), 2.53 (s, 3H, CH_3). ^{13}C NMR (101 MHz, $CDCl_3$) δ 151.5, 139.0 (d, $^2J_{CP}$ = 9.9 Hz), 134.7, 127.5, 114.4, 113.7, 53.1 (d, $^2J_{CP}$ = 6.9 Hz), 25.4 (d, $^1J_{CP}$ = 141.8 Hz), 19.3. ^{31}P NMR (162 MHz, $CDCl_3$) δ 23.00. ν_{max}(KBr)/cm^{-1} 3420, 3273, 2360, 1651, 1573, 1518, 1446, 1232, 1199, 1107, 1043, 486, 798, 769, 746, 696. HRMS-ESI (m/z): calcd for $C_{10}H_{14}N_3O_3P$, $[M+H]^+$: 256.0846, found 256.0852.

Diisopropyl ((5-methyl-[1,2,4]triazolo[4,3-a]pyridin-3-yl)methyl)phosphonate 8b

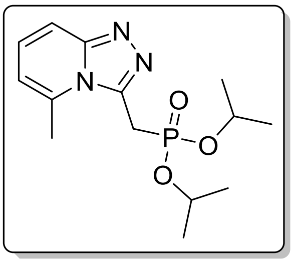

Following the general procedure, **8b** was obtained from **1f** and **2b** as pale yellow oil. ^{1}H NMR (400 MHz, $CDCl_3$) δ 7.56 (d, $^3J_{HH}$ = 9.2 Hz, 1H, 8-H$_{ar.}$), 7.09 (dd, $^3J_{HH}$ = 9.2, 6.7 Hz, 1H, 7-H$_{ar.}$), 6.51 (d, $^3J_{HH}$ = 6.6 Hz, 1H, 6-H$_{ar.}$), 4.79 – 4.40 (m, 2H, 2OCH), 3.92 (d, $^2J_{HP}$ = 20.6 Hz, 2H, CH_2), 2.97 (s, 3H, CH_3) 1.40 – 1.05 (m, 12H, $4CH_3$). ^{13}C NMR (101 MHz, $CDCl_3$) δ 152.0, 139.8 (d, $^2J_{CP}$ = 10.5 Hz), 134.9, 127.4, 114.5, 114.5, 71.9 (d, $^2J_{CP}$ = 7.0 Hz) 27.7 (d, $^1J_{CP}$ = 143.3 Hz), 24.0 (d, $^3J_{CP}$ = 3.8 Hz), 23.8 (d, $^3J_{CP}$ = 5.1 Hz), 19.9. ^{31}P NMR (162 MHz, $CDCl_3$) δ 18.65. ν_{max} (KBr)/cm^{-1} 2978, 1640, 1512, 1465, 1383, 1245, 1107, 994, 791. HRMS-ESI (m/z): calcd for $C_{14}H_{22}N_3O_3P$, $[M+H]^+$: 312.1472, found 312.1479.

Dimethyl ((6-nitro-[1,2,4]triazolo[4,3-a]pyridin-3-yl)methyl)phosphonate 10a

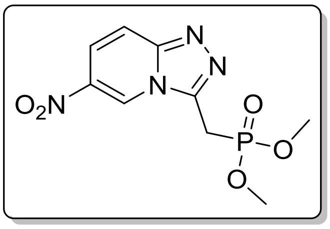

Following the general procedure, **10a** was obtained from **1j** and **2a** as pale yellow oil. ^{1}H NMR (400 MHz, CDCl3) δ 9.48 (s, 1H, 5-H$_{ar.}$), 8.05 (d, $^3J_{HH}$ = 10.0 Hz, 1H, 8-H$_{ar.}$), 7.86 (d, $^3J_{HH}$ = 10.0 Hz, 1H, 7-H$_{ar.}$), 3.91 (d, $^2J_{HP}$ = 20.6 Hz, 2H, CH_2), 3.82 (d, $^3J_{HP}$ = 11.1 Hz, 6H, $2CH_3$). ^{13}C NMR (101 MHz, $CDCl_3$) δ 149.9, 141.9 (d, $^2J_{CP}$ = 10.1 Hz), 138.4, 124.6, 121.5, 116.6, 53.7 (d, $^2J_{CP}$ = 6.7 Hz), 23.6 (d,$^1J_{CP}$= 142.5 Hz). ^{31}P NMR (162 MHz, $CDCl_3$) δ 21.88. ν_{max}(KBr)/cm^{-1} 3038, 2361, 1636, 1527, 1473, 1257, 1030, 866, 827, 794, 756, 534. HRMS-ESI (m/z): calcd for $C_9H_{12}N_4O_5P$, $[M+H]^+$: 287.0540, found 287.0548

Dimethyl ((8-nitro-[1,2,4]triazolo[1,5-a]pyridin-3-yl)methyl)phosphonate 11a

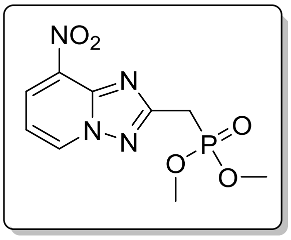

Following the general procedure, **11a** was obtained from **1i** and **2a** as pale solid. ^{1}H NMR (400 MHz, $CDCl_3$) δ 8.90 (dd, $^3J_{HH}$ = 6.7,$^4J_{HH}$= 1.2 Hz, 1H, 5-H$_{ar.}$), 8.57 (dd, $^3J_{HH}$ = 7.9,$^3J_{HH}$= 1.2 Hz, 1H, 7-H$_{ar.}$), 7.23 (dd, $^3J_{HH}$ = 7.9, 6.7 Hz, 1H, 6-H$_{ar.}$), 3.90 (d, $^2J_{HP}$= 11.1 Hz, 6H, $2CH_3$), 3.71 (d, $^2J_{HP}$ = 21.6 Hz, 2H, CH_2).^{13}C NMR (101 MHz, $CDCl_3$) δ 161.9 (d, $^2J_{CP}$ = 8.1 Hz), 145.5, 136.5, 133.9, 128.1, 111.6, 53.4 (d, $^2J_{CP}$ = 6.5 Hz), 27.0 (d, $^1J_{CP}$ = 140.2 Hz). ^{31}P NMR (162 MHz, $CDCl_3$) δ 24.68. ν_{max} (KBr)/cm^{-1} 3037, 2361, 1635, 1528, 1474, 1296, 1257, 1215, 1030, 989, 866, 827, 795, 756, 534. HRMS-ESI (m/z): calcd for $C_9H_{12}N_4O_5P$, $[M+H]^+$: 287.0540, found 287.0553. M. p. 104 - 105 °C.

Diisopropyl ((8-nitro-[1,2,4]triazolo[1,5-a]pyridin-3-yl)methyl)phosphonate 11b

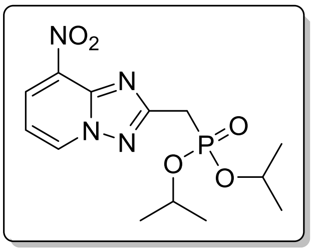

Following the general procedure, **11b** was obtained from **1i** and **2b** as pale solid. ^{1}H NMR (400 MHz, CDCl3) δ 8.88 (dd, $^3J_{HH}$ = 6.8,$^4J_{HH}$= 1.1 Hz, 1H, 5-H$_{ar.}$), 8.53 (dd, $^3J_{HH}$ = 7.8,$^3J_{HH}$= 1.1 Hz, 1H, 7-H$_{ar.}$), 7.20 (dd, $^3J_{HH}$ = 7.8, 6.8 Hz, 1H, 6-H$_{ar.}$), 4.80 (m, 2H), 3.62 (d, ^{2}J$_{HP}$ = 21.5 Hz, 1H), 1.36 (d, $^3J_{HH}$ = 5.6 Hz, 3H, CH_3),1.35 (d, $^3J_{HH}$ = 5.6 Hz, 3H, CH_3).^{13}C NMR (101 MHz, $CDCl_3$) δ 162.4 (d, $^2J_{CP}$ = 8.1 Hz), 145.4, 136.4, 133.9, 128.0, 111.4, 71.6 (d, $^2J_{CP}$ = 6.6Hz), 28.9 (d, $^1J_{CP}$ = 140.5 Hz), 24.1 (d, J = 3.6 Hz), 23.9 (d, J = 5.2 Hz). ^{31}P NMR (162 MHz, $CDCl_3$) δ 19.71. ν$_{max}$ (KBr)/cm^{-1} 3046, 2982, 2932, 1638, 1528, 1477, 1383, 1255, 1244, 1109, 976, 785, 756, 681, 540. HRMS-ESI (m/z): calcd for $C_{13}H_{19}N_4O_5P$, [M+H]$^+$: 343.2875, found 343.1166. M. p. 89 - 90 °C.

Dimethyl ((6-nitro-[1,2,4]triazolo[1,5-a]pyridin-3-yl)methyl)phosphonate 12a

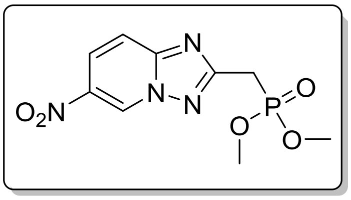

Following the general procedure, **12a** was obtained from **1j** and **2a** as pale yellow oil. 1H NMR (400 MHz, $CDCl_3$) δ 9.61 (d, $^4J_{HH}$ = 2.1 Hz, 1H, 5-H$_{ar.}$), 8.34 (dd, $^3J_{HH}$ = 9.8 Hz,$^4J_{HH}$ = 2.1 Hz, 1H, 7-H$_{ar.}$), 7.82 (d, $^3J_{HH}$ = 9.8 Hz, 1H, 8-H$_{ar.}$), 3.87 (d, $^3J_{HP}$ = 21.6 Hz, 2H, CH_2), 3.63 (d, $^2J_{HP}$ = 11.1 Hz, 6H, 2CH_3). ^{13}C NMR (101 MHz, $CDCl_3$) δ 163.3 (d, $^2J_{CP}$ = 8.6 Hz), 152.7, 137.9, 127.7, 124.2, 115.9, 53.3 (d, $^2J_{CP}$ = 6.1 Hz), 27.0 (d,$^1J_{CP}$= 140.1 Hz).^{31}P NMR (162 MHz, CDCl3) δ 24.53. ν$_{max}$ (KBr)/cm^{-1} 3075, 2918, 2361, 2342, 1643, 1526, 1472, 1389, 1350, 1252, 1022, 862, 798, 735, 532. HRMS-ESI (m/z): calcd for $C_9H_{12}N_4O_5P$, [M+H]$^+$: 287.1812, found 287.0540.

Diisopropyl ((6-nitro-[1,2,4]triazolo[4,3-a]pyridin-3-yl)methyl)phosphonate 12b

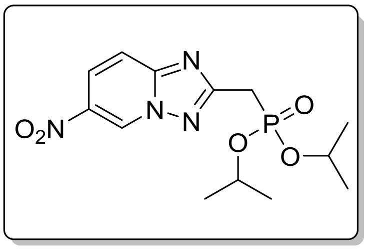

Following the general procedure, **12b** was obtained from **1j** and **2b** as pale yellow oil. ^{1}H NMR (400 MHz, $CDCl_3$) δ 9.60 (dd, $^3J_{HH}$ = 2.2, $^4J_{HH}$ = 0.8 Hz, 1H, 5-H$_{ar.}$), 8.32 (dd, $^3J_{HH}$ = 9.8, $^3J_{HH}$ = 2.2 Hz, 1H, 7-H$_{ar.}$), 7.80 (dd, $^3J_{HH}$ = 9.8, 0.8 Hz, 1H, 6-H$_{ar.}$), 4.80 (m, 2H), 3.56 (d, ^{2}J$_{HP}$ = 21.6 Hz, 2H, CH_2), 1.36 (d, $^3J_{HH}$ = 6.1 Hz, 3H, CH_3), 1.34 (d, $^3J_{HH}$ = 6.1 Hz, 3H, CH_3).^{13}C NMR (101 MHz, $CDCl_3$) δ 163.9 (d, $^2J_{CP}$ = 8.5 Hz), 152.6, 137.8, 127.6, 124.0, 115.8, 71.5 (d, $^2J_{CP}$ = 6.6 Hz), 29.2 (d, $^1J_{CP}$ = 140.4 Hz), 24.1 (d, J = 3.7 Hz), 23.9 (d, J = 5.1 Hz). ^{31}P NMR (162 MHz, $CDCl_3$) δ 19.65. ν$_{max}$ (KBr)/cm^{-1} 3078, 2982, 1639, 1558, 1529, 1476, 1348, 1234, 1107, 982, 783, 761, 734, 529. HRMS-ESI (m/z): calcd for $C_{13}H_{19}N_4O_5P$, [M+H]$^+$: 343.2875, found 343.1166

Dimethyl ([1,2,4]triazolo[4,3-a]quinolin-1-ylmethyl)phosphonate 13a

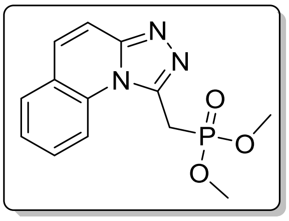

Following the general procedure, 1**3a** was obtained from **1g** and **2a** as pale solid. ^{1}H NMR (400 MHz, $CDCl_3$) δ 8.85 – 8.48 (m, 1H), 7.99 (d, $^{3}J_{HH}$ = 7.4 Hz, 1H), 7.81 – 7.56 (m, 3H), 7.10 (d, $^{3}J_{HH}$ = 7.4 Hz, 1H), 3.80 (d, $^{2}J_{HP}$ = 20.4 Hz, 2H, CH_2), 3.77 (d, $^{3}J_{HP}$ = 11.1 Hz, 6H, $2OCH_3$). ^{13}C NMR (101 MHz, $CDCl_3$) δ 149.3, 140.6 (d, $^{2}J_{CP}$ = 9.8 Hz), 130.2, 129.7, 129.1, 127.1, 123.9, 121.4, 119.8, 115.2, 53.5 (d, $^{2}J_{CP}$ = 6.7 Hz), 23.4 (d, $^{1}J_{CP}$ = 142.6 Hz).^{31}P NMR (162 MHz, $CDCl_3$) δ 22.75. ν_{max}(KBr)/cm^{-1} 3473, 2941, 1661, 1643, 1524, 1453, 1401, 1130, 1063, 1023, 898, 885, 839, 808, 790. HRMS-ESI (m/z): calcd for $C_{13}H_{14}N_3O_3P$, $[M+H]^+$: 292.0846, found 292.0839. M. p. 108 - 109 °C.

Diisopropyl ([1,2,4]triazolo[4,3-a]quinolin-1-ylmethyl)phosphonate 13b

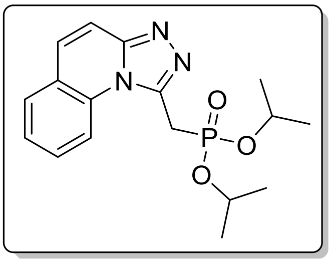

Following the general procedure, **13b** was obtained from **1g** and **2b** as pale solid. ^{1}H NMR (400 MHz, $CDCl_3$) δ8.79 – 8.68 (m, 1H), 8.10 (d, $^{3}J_{HH}$ = 7.4 Hz, 1H), 7.89 – 7.57 (m, 3H), 7.10 (d, $^{3}J_{HH}$ = 7.4 Hz, 1H), 4.67 (m, 2H, 2OCH), 3.75 (d, $^{2}J_{HP}$ = 20.4 Hz, 2H, CH_2), 1.29 (d, $^{3}J_{HH}$= 6.5 Hz, 6H), 1.28 (d, , $^{3}J_{HH}$ = 6.5 Hz, 6H). ^{13}C NMR (101 MHz, $CDCl_3$) δ 149.3, 141.2 (d, $^{2}J_{CP}$ = 9.6 Hz), 130.1, 129.7, 129.0, 127.1, 124.0, 121.5, 120.4, 114.7, 72.1 (d, $^{2}J_{CP}$ = 6.9 Hz), 25.5 (d, $^{1}J_{CP}$ = 143.2 Hz), 24.0 (d, $^{3}J_{CP}$ = 4.2 Hz), 24.1 (d, $^{3}J_{CP}$ = 4.8 Hz). ^{31}P NMR (162 MHz, $CDCl_3$) δ 18.40. ν_{max} (KBr)/cm^{-1} 3464, 2977, 1642, 1524, 1460, 1377, 1242, 1107, 999, 976, 794. HRMS-ESI (m/z): calcd for $C_{17}H_{22}N_3O_3P$, $[M+H]^+$: 348.1472, found 348.1486. M. p. 90 - 91 °C.

Diethyl ([1,2,4]triazolo[4,3-a]quinolin-1-ylmethyl)phosphonate 13c

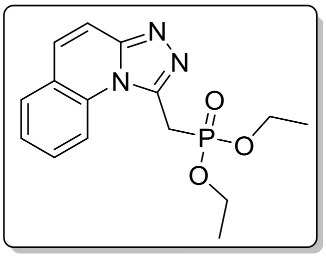

Following the general procedure, **13c** was obtained from **1g** and **2c** as pale yellow oil. ^{1}H NMR (400 MHz, $CDCl_3$) δ 8.38 (d, ,$^{3}J_{HH}$ = 7.9Hz, 1H), 7.77 (d, $^{3}J_{HH}$ = 7.9 Hz, 1H), 7.67 (t, $^{3}J_{HH}$ = 7.9 Hz, 1H), 7.58 (dd, $^{3}J_{HH}$ = 7.9 Hz, 1H), 7.55 – 7.46 (m,2H), 4.18– 4.08 (m, 2H, CH_2), 4.14 (d, $^{2}J_{HP}$ = 20.3 Hz, 4H, $2OCH_2$), 1.22 (d, $^{3}J_{HH}$ = 7.1 Hz, 3H, CH_3),1.20 (d, $^{3}J_{HH}$ = 7.1 Hz, 3H, CH_3).^{13}C NMR (101 MHz, $CDCl_3$) δ 150.3, 142.0 (d, $^{2}J_{CP}$ = 9.7 Hz), 131.8, 129.3, 129.2, 126.3, 124.6, 116.9, 114.9, 63.1 (d, $^{2}J_{CP}$ = 7.0 Hz), 27.8 (d, $^{1}J_{CP}$ = 142.4 Hz), 16.2 (d, $^{2}J_{CP}$ = 6.4 Hz). ^{31}P NMR (162 MHz, $CDCl_3$) δ 19.71. ν_{max} (KBr)/cm^{-1} 3421, 2982, 1657, 1605, 1504, 1447, 1372, 1232, 1050, 1026, 969, 830, 811, 758. HRMS-ESI (m/z): calcd for $C_{15}H_{18}N_3O_3P$, $[M+Na]^+$: 342.0978, found 342.0991.

Dimethyl ([1,2,4]triazolo[4,3-a]isoquinolin-3-ylmethyl)phosphonate 14a

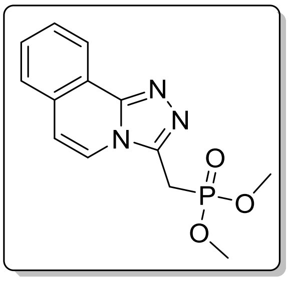

Following the general procedure, **14a** was obtained from **1h** and **2a** as pale solid. ^{1}H NMR (400 MHz, $CDCl_3$) δ 8.40 (d, $^3J_{HH}$ = 8.6 Hz, 1H), 7.83 (dd, $^3J_{HH}$= 8.6, $^4J_{HH}$= 1.4 Hz, 1H), 7.78 – 7.69 (m, 1H), 7.65 (d, $^3J_{HH}$ = 8.6 Hz, 1H), 7.61 – 7.53 (m, 1H), 4.19 (d, $^2J_{HP}$ = 20.5 Hz, 2H, CH_2), 3.82 (d, $^2J_{HP}$ = 11.1 Hz, 6H, $2OCH_3$). ^{13}C NMR (101 MHz, $CDCl_3$) δ 150.4, 141.8 (d, $^2J_{CP}$ = 9 Hz), 131.9, 129.8, 129.4, 126.4, 124.8, 116.6, 115.1, 53.6 (d, $^2J_{CP}$ = 7.2 Hz), 27.2 (d, $^1J_{CP}$ = 143.2 Hz).^{31}P NMR (162 MHz, $CDCl_3$) δ 22.23. ν_{max}(KBr)/cm^{-1} 4422, 2957, 1622, 1561, 1412, 1260, 1244, 1049, 1014, 864, 822. HRMS-ESI (m/z): calcd for $C_{13}H_{14}N_3O_3P$, $[M+H]^+$: 292.0846, found 292.0836. M. p. 132 - 133 °C.

Diisopropyl ([1,2,4]triazolo[4,3-a]isoquinolin-3-ylmethyl)phosphonate 14b

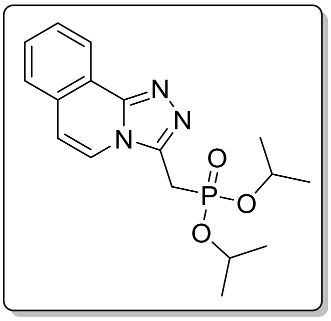

Following the general procedure, **14b** was obtained from **1h** and **2b** as pale solid. ^{1}H NMR (400 MHz, $CDCl_3$) δ 8.43 (d, J = 8.6 Hz, 1H), 7.78 (d, $^3J_{HH}$ = 8.6 Hz, 1H), 7.68 (t, $^3J_{HH}$ = 8.6 Hz, 1H), 7.60 (d, $^3J_{HH}$= 8.6 Hz, 1H), 7.56 – 7.48 (m, 2H), 4.89 – 4.55 (m, 2H), 4.12 (d, $^2J_{HP}$ = 20.3 Hz, 2H, CH_2), 1.23 (d, $^3J_{HH}$ = 6.2 Hz, 12H, $4CH_3$). ^{13}C NMR (101 MHz, $CDCl_3$) δ 150.3, 142.4 (d, $^2J_{CP}$ = 10.0 Hz), 132.0, 129.6, 129.2, 129.1, 126.2, 124.6, 117.3, 115.1, 72.1 (d,$^2J_{CP}$= 7.2 Hz), 29.0 (d, $^1J_{CP}$ = 143.6 Hz), 23.9 (d, J = 5.2 Hz), 23.8 (d, J = 5.2 Hz). ^{31}P NMR (162 MHz, $CDCl_3$) δ 18.40. ν_{max}(KBr)/cm^{-1} 3442, 2976, 1656, 1625, 1563, 1539, 1411, 1376, 1276, 1224, 1105, 1022, 1001, 810. HRMS-ESI (m/z): calcd for $C_{17}H_{22}N_3O_3P$, $[M+H]^+$: 348.1472, found 348.1476. M. p. 117 - 118 °C.

Diethyl ([1,2,4]triazolo[4,3-a]isoquinolin-3-ylmethyl)phosphonate 14c

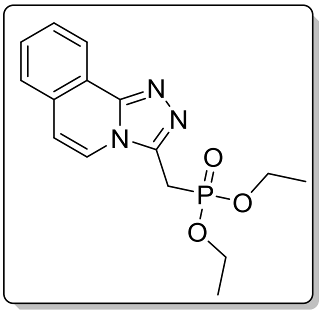

Following the general procedure, **14c** was obtained from **1h** and **2c** as pale solid. ^{1}H NMR (400 MHz, $CDCl_3$) δ 8.49 (d, ,$^3J_{HH}$ = 8.7 Hz, 1H), 7.82 (dd, $^3J_{HH}$ = 7.5, $^4J_{HH}$ = 1.6 Hz, 1H), 7.75 – 7.70 (m,$^3J_{HH}$ = 8.7, $^3J_{HH}$ = 7.5,$^4J_{HH}$ = 1.6 Hz, 1H), 7.64 (d, $^3J_{HH}$ = 8.7 Hz, 1H), 7.60 – 7.52 (m, 2H), 4.74 (m, $^3J_{HP}$ = 7.5 Hz, $^3J_{HH}$ = 6.2 Hz, 2H, CH_2), 4.15 (d, $^2J_{HP}$ = 20.2 Hz, 4H, $2OCH_2$), 1.26 (d, $^3J_{HH}$= 6.2 Hz, 6H, $2CH_3$).^{13}C NMR (101 MHz, $CDCl_3$) δ 150.4, 142.4 (d, $^2J_{CP}$ = 9.7 Hz), 132.0, 129.6, 129.1, 126.3, 124.7, 117.3, 115.1, 72.1 (d, $^2J_{CP}$ = 7.1 Hz), 29.0 (d, $^1J_{CP}$ = 143.8 Hz), 24.0 (d, $^2J_{CP}$ = 5.2 Hz), 23.8 (d, $^2J_{CP}$ = 5.2 Hz). ^{31}P NMR (162 MHz, $CDCl_3$) δ 17.83. ν_{max} (KBr)/cm^{-1} 3501, 3328, 3234, 2982, 2888, 1643, 1529, 1235, 1047, 1023, 958, 782. HRMS-ESI (m/z): calcd for $C_{13}H_{14}N_3O_3P$, $[M+H]^+$: 320.1159, found 320.1150. M. p. 58 - 59 °C.

E. NMR Spectra

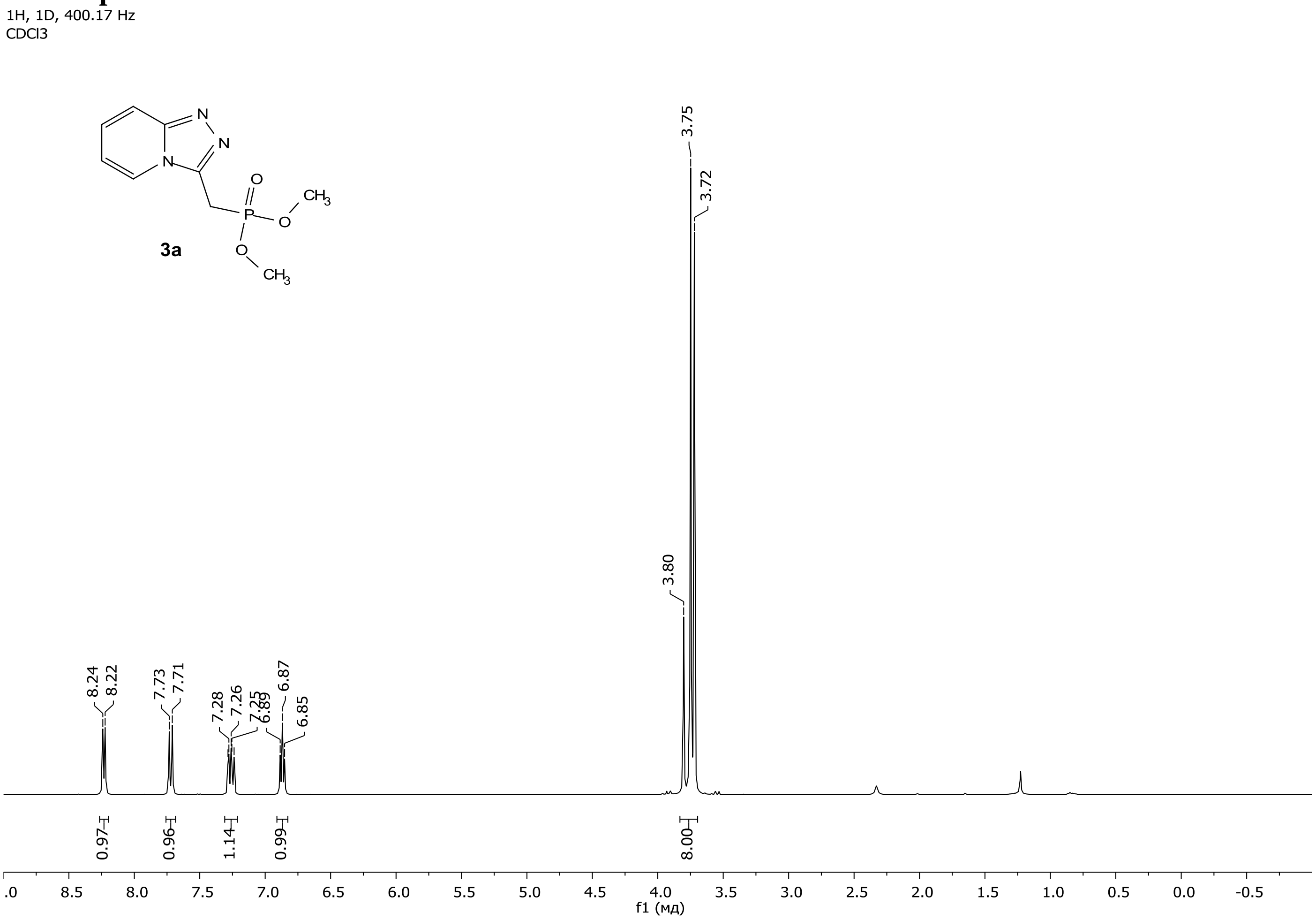

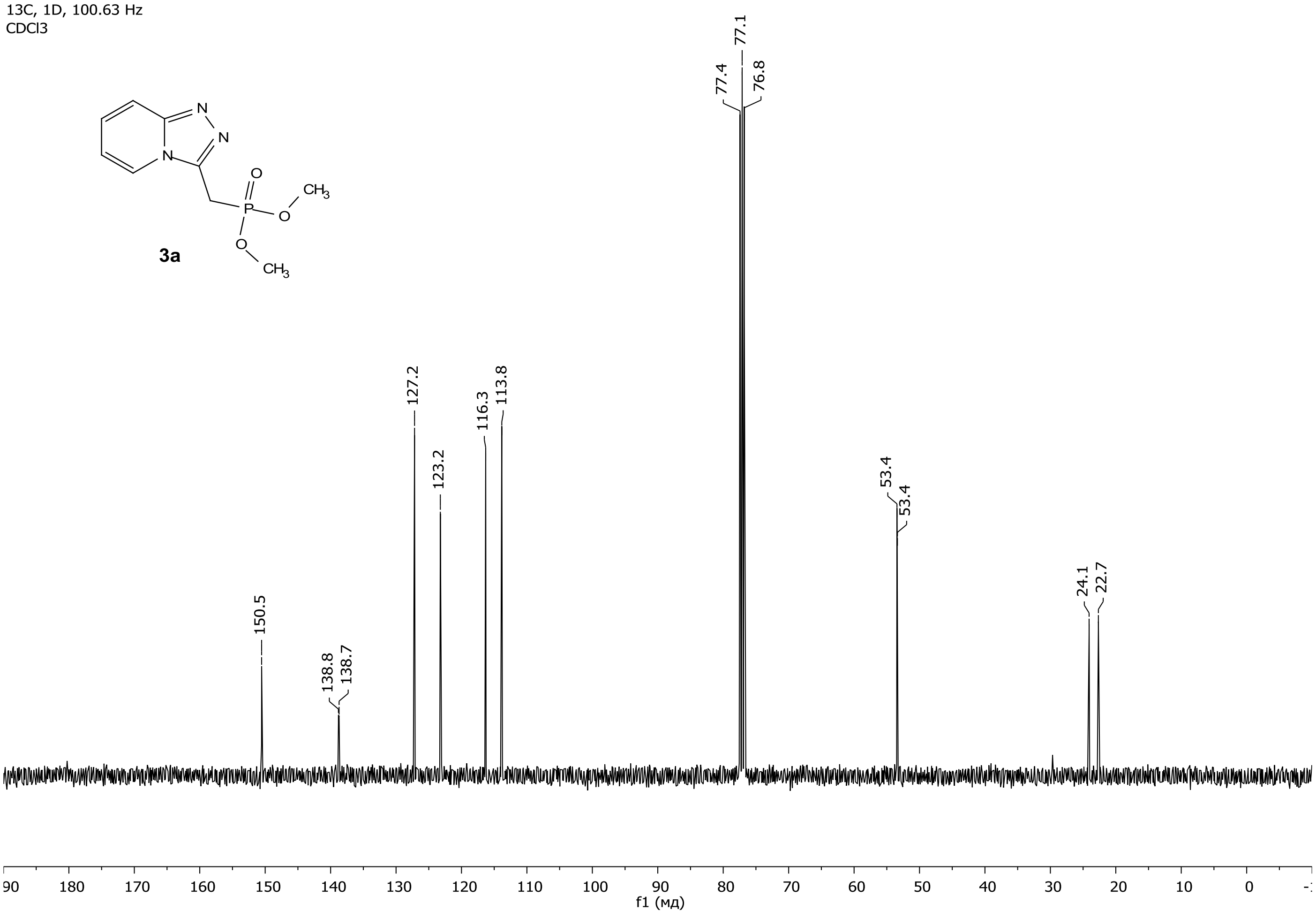

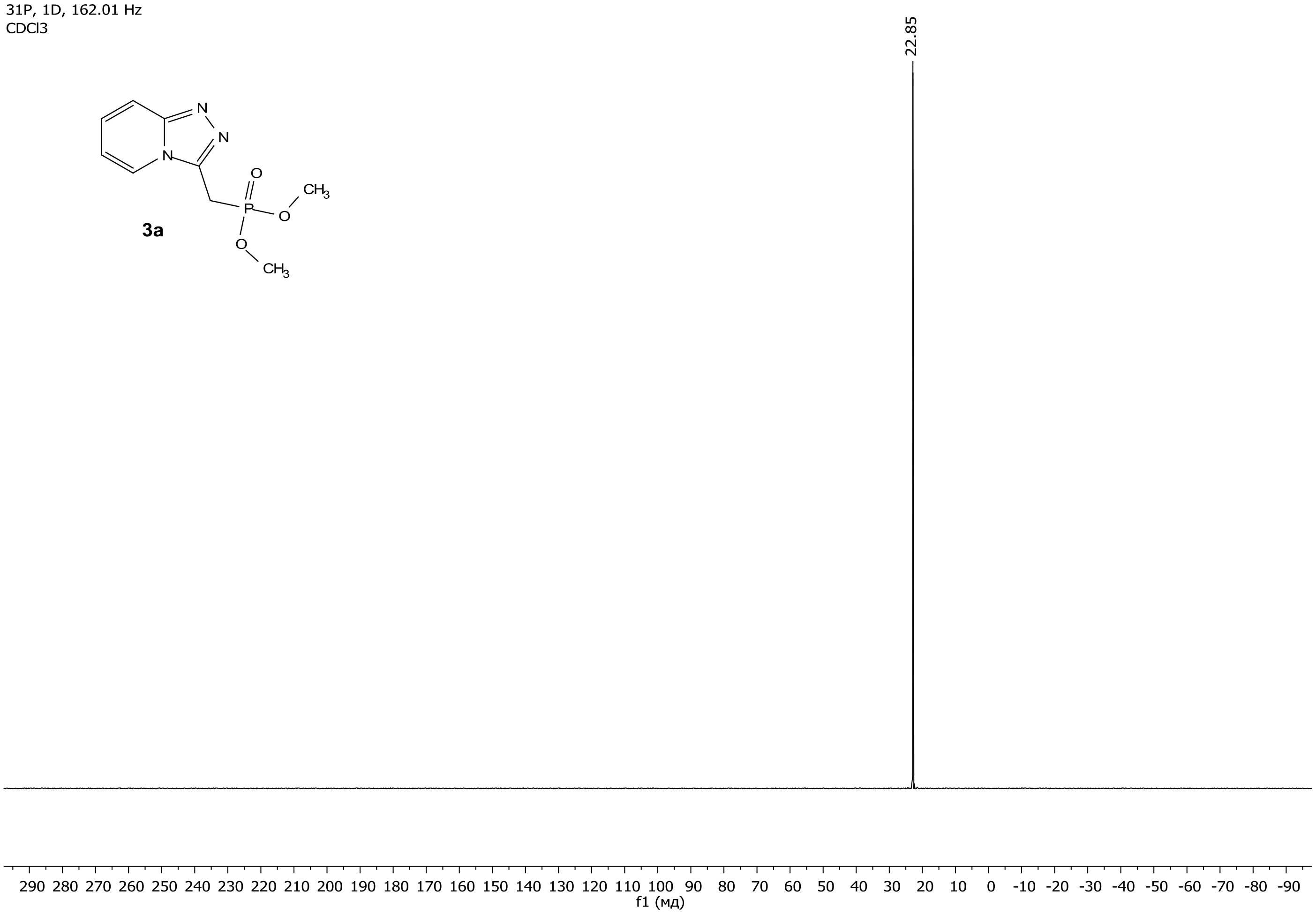
31P, 1D, 162.01 Hz
CDCl3
3a
22.85
f1 (мд)

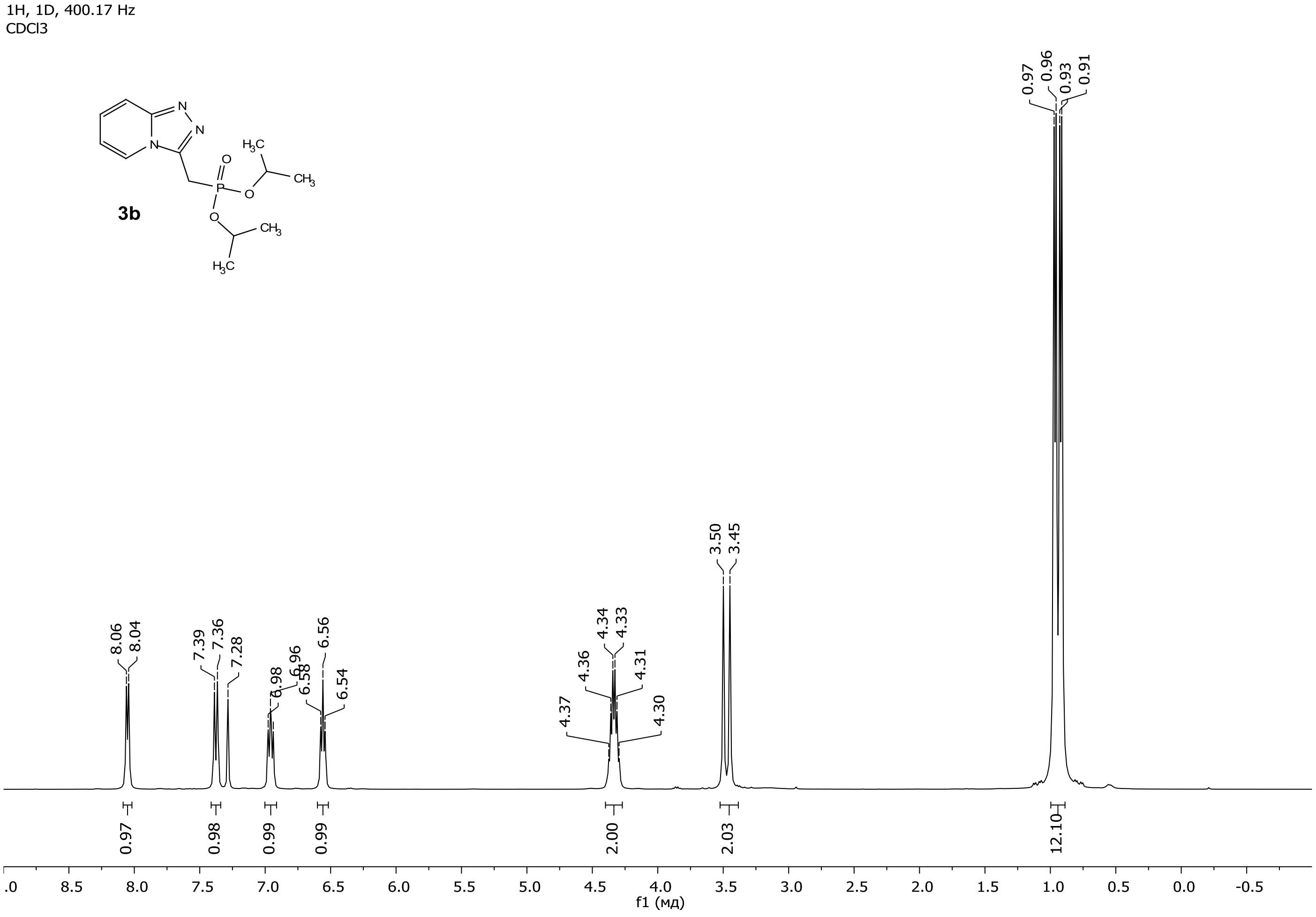
1H, 1D, 400.17 Hz
CDCl3
3b
8.06 8.04 7.39 7.36 7.28 6.98 6.96 6.58 6.56 6.54
4.37 4.36 4.34 4.33 4.31 4.30
3.50 3.45
0.97 0.96 0.93 0.91
0.97 0.98 0.99 0.99 2.00 2.03 12.10
f1 (мд)

13C, 1D, 100.63 Hz
Chloroform

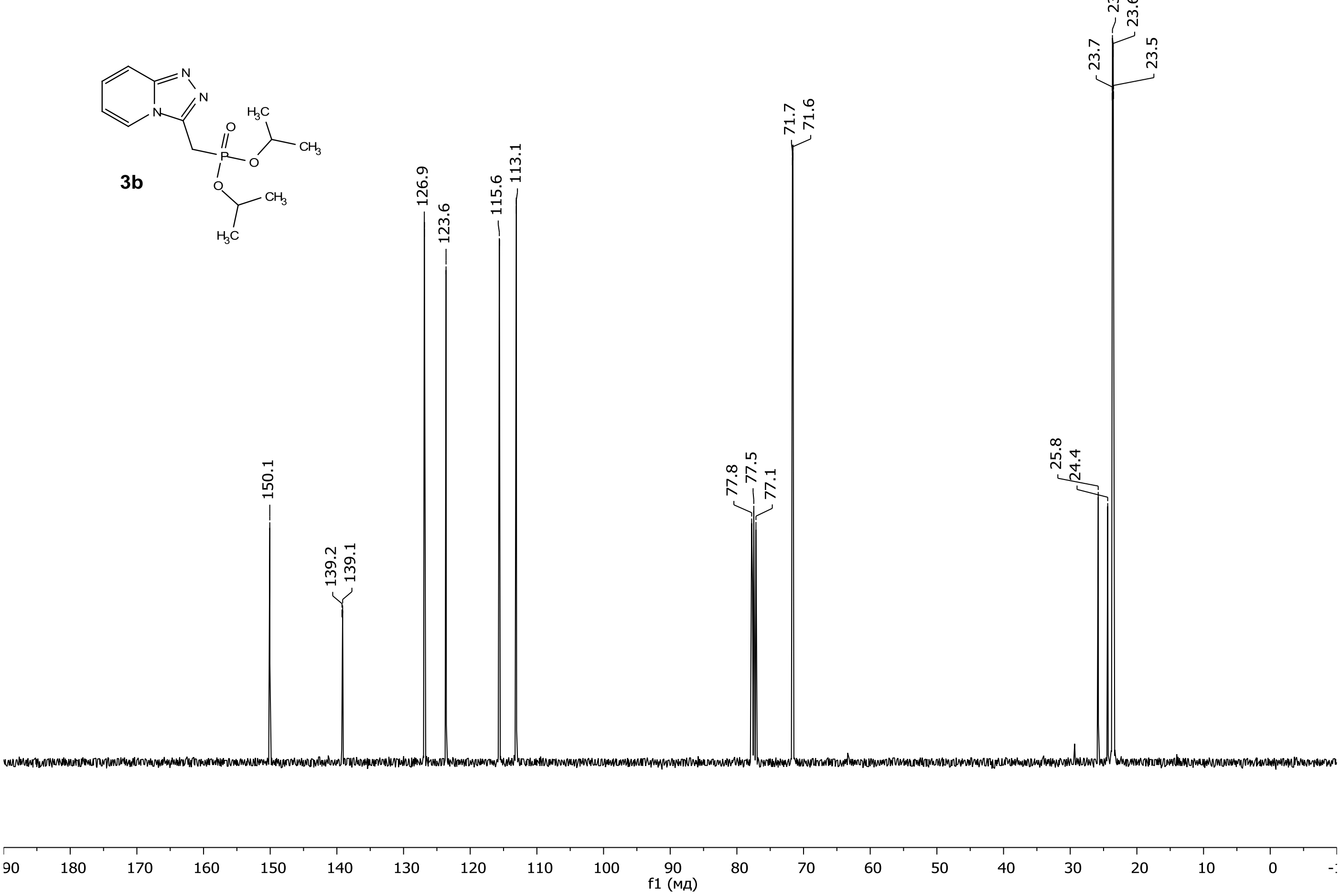

31P, 1D, 162.01 Hz
CDCl3

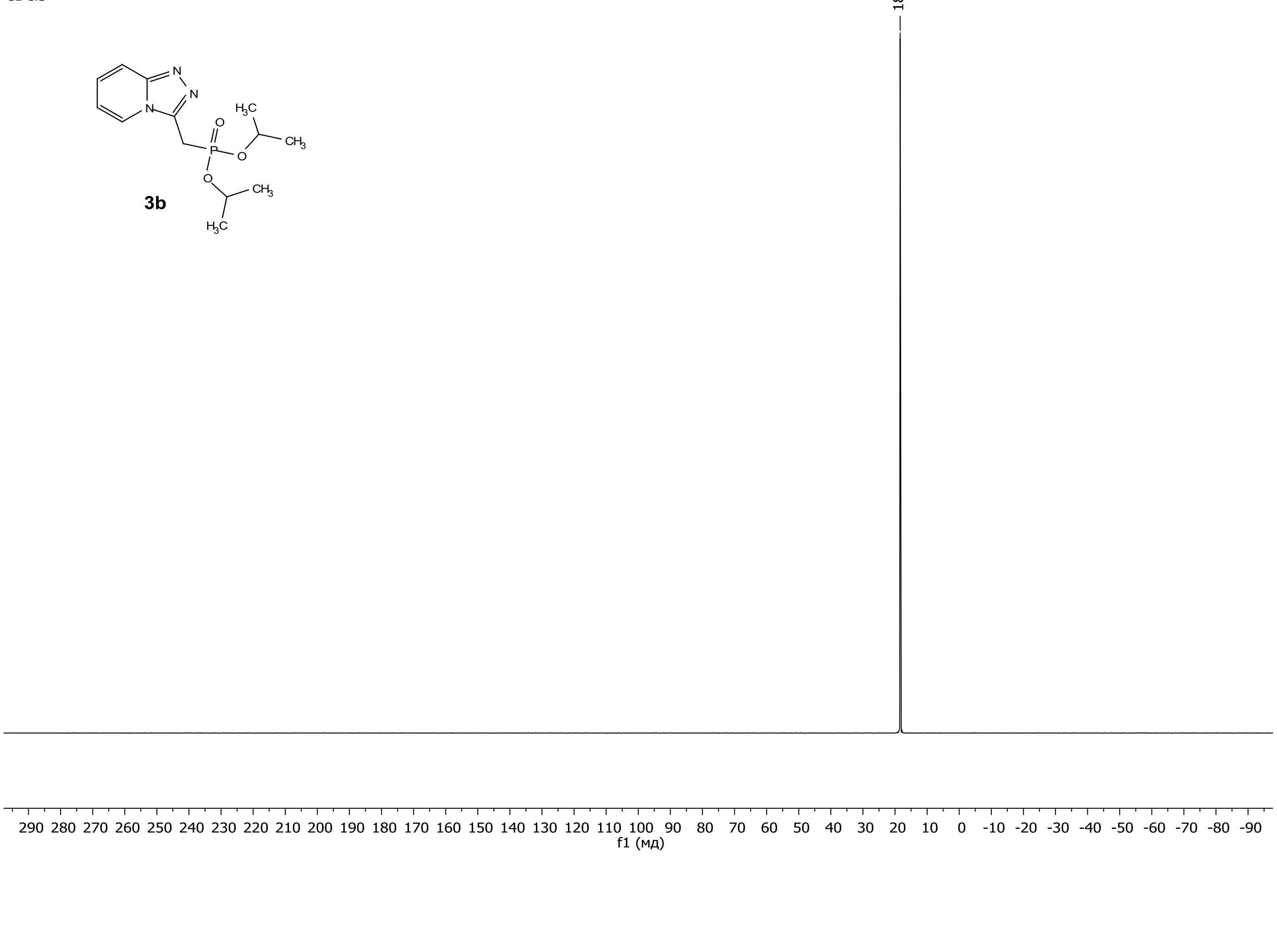

1H, 1D, 400.17 Hz
CDCl3

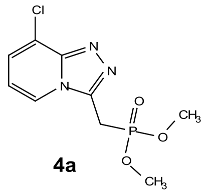

4a

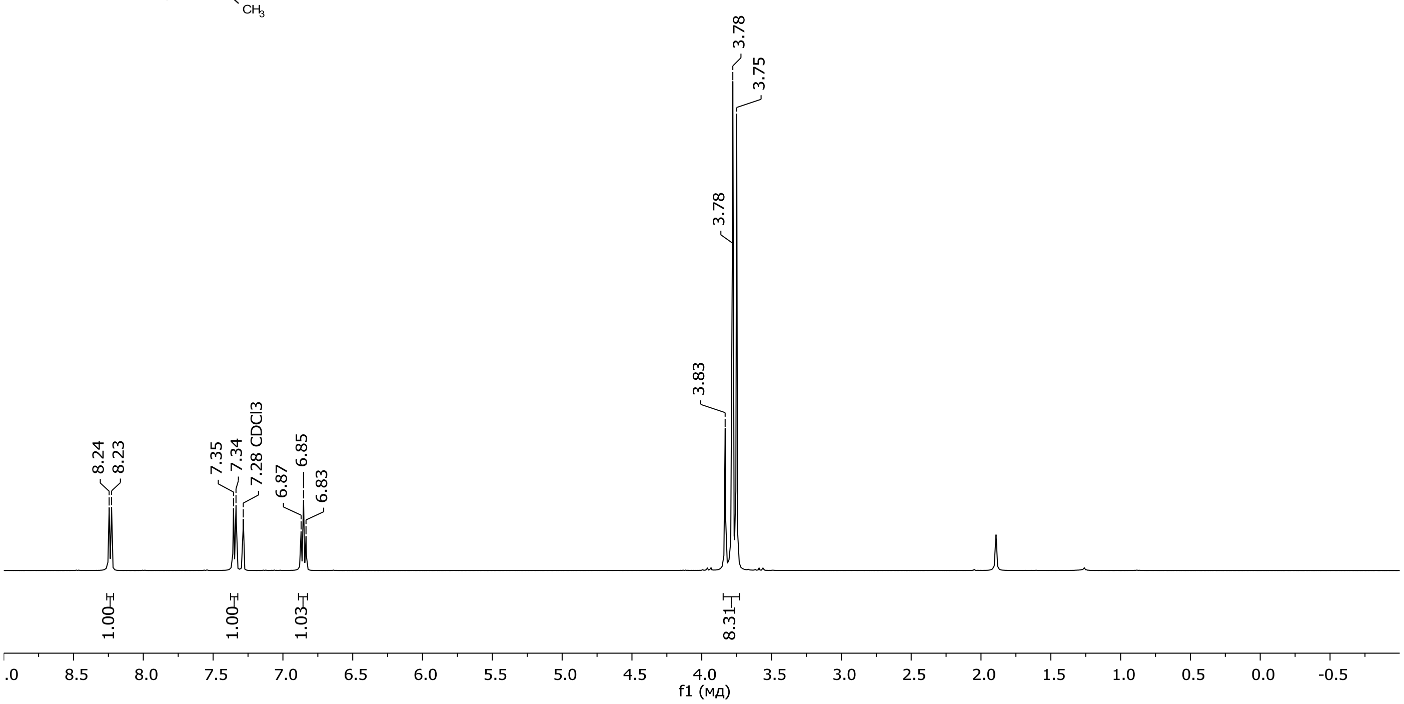

13C, 1D, 100.63 Hz
CDCl3

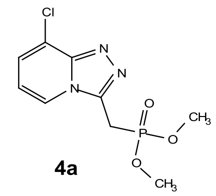

4a

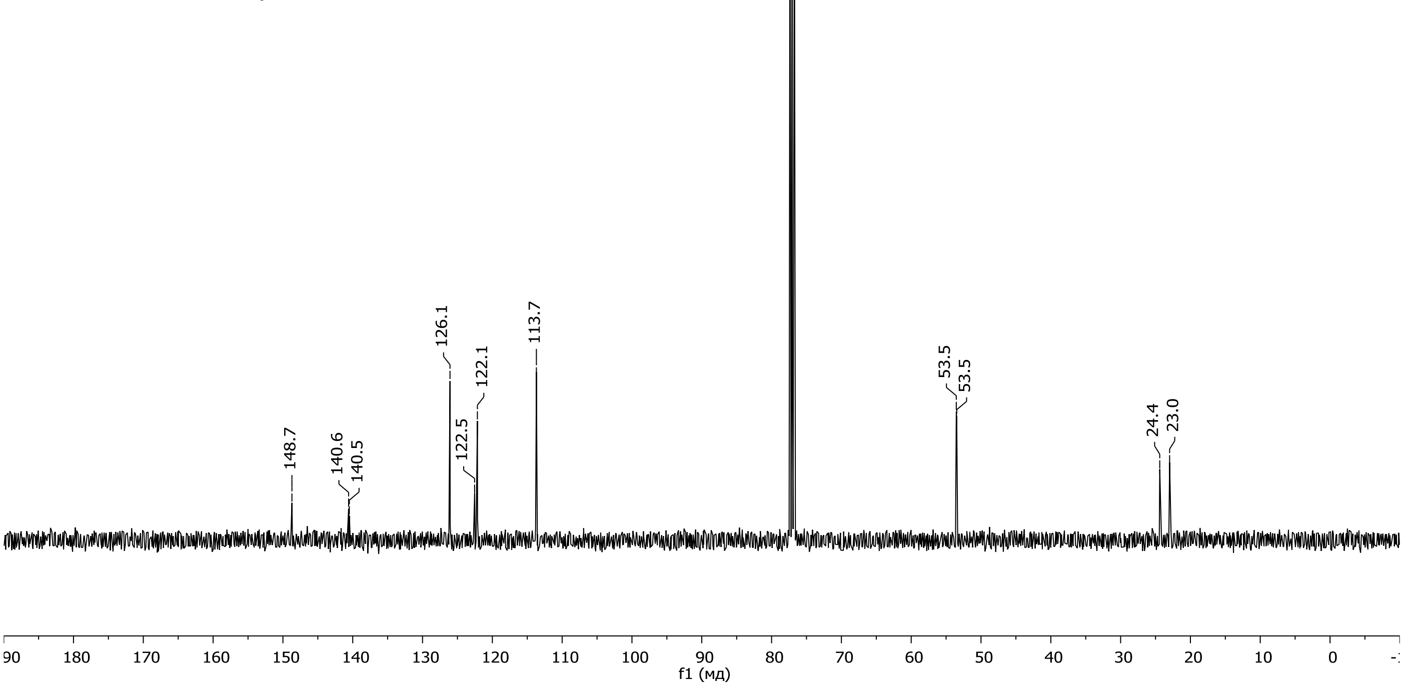

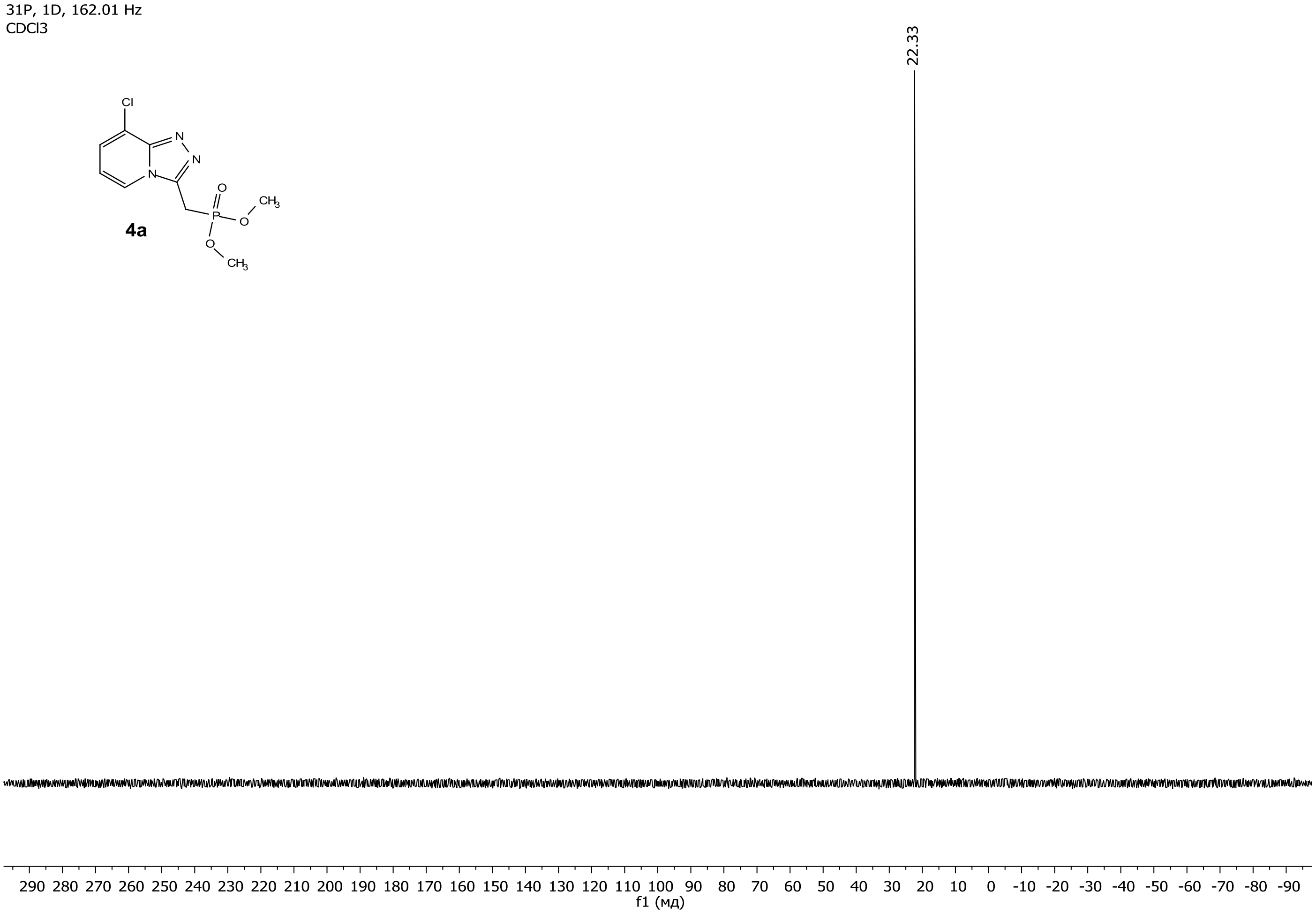
31P, 1D, 162.01 Hz
CDCl3
4a
22.33
f1 (мд)

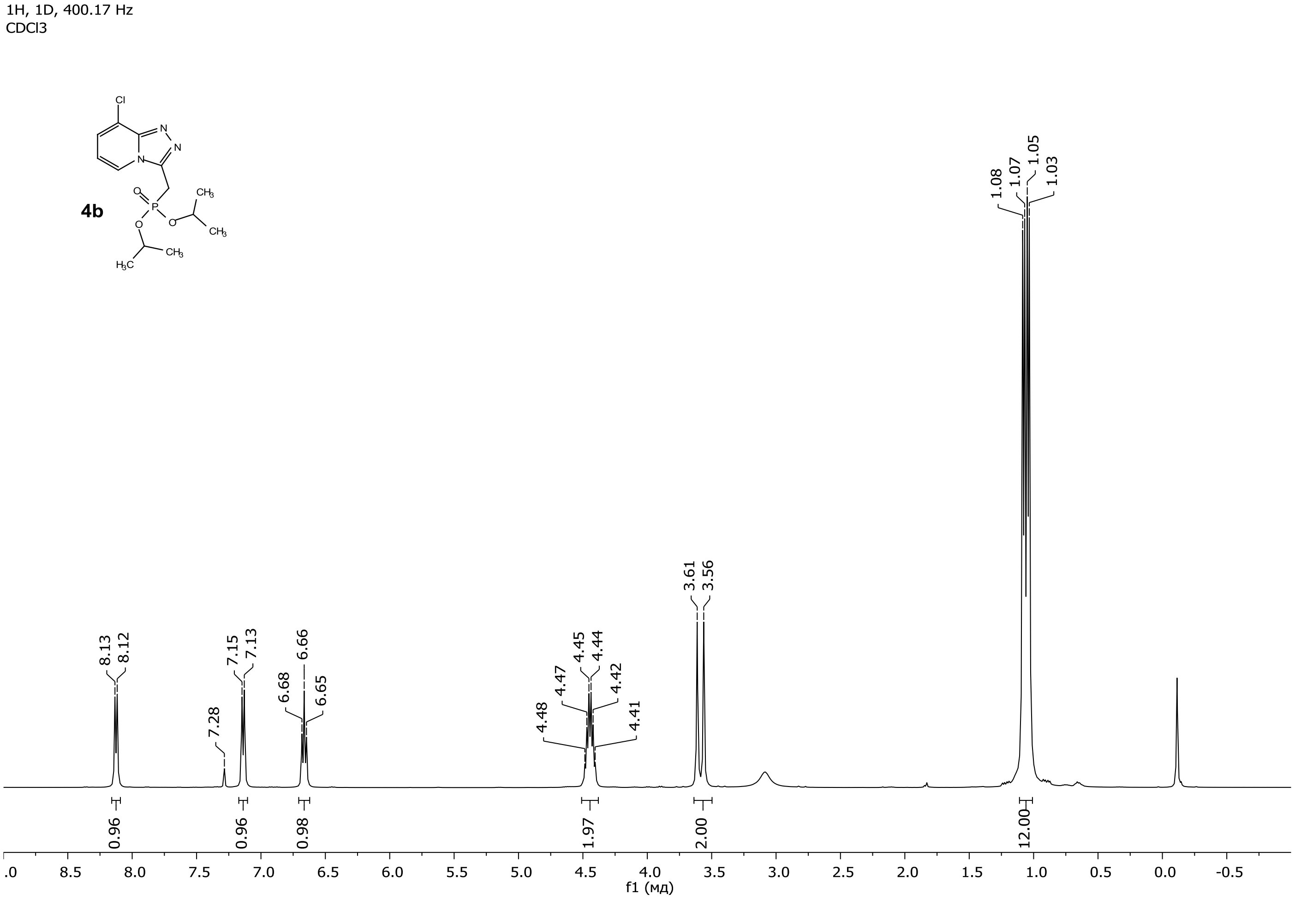
1H, 1D, 400.17 Hz
CDCl3
4b
8.13 8.12
7.28
7.15 7.13
6.68 6.66 6.65
4.48 4.47 4.45 4.44 4.42 4.41
3.61 3.56
1.08 1.07 1.05 1.03
0.96 0.96 0.98 1.97 2.00 12.00
f1 (мд)

13C, 1D, 100.63 Hz
CDCl3

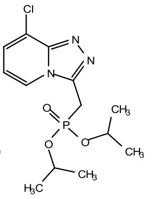

4
b

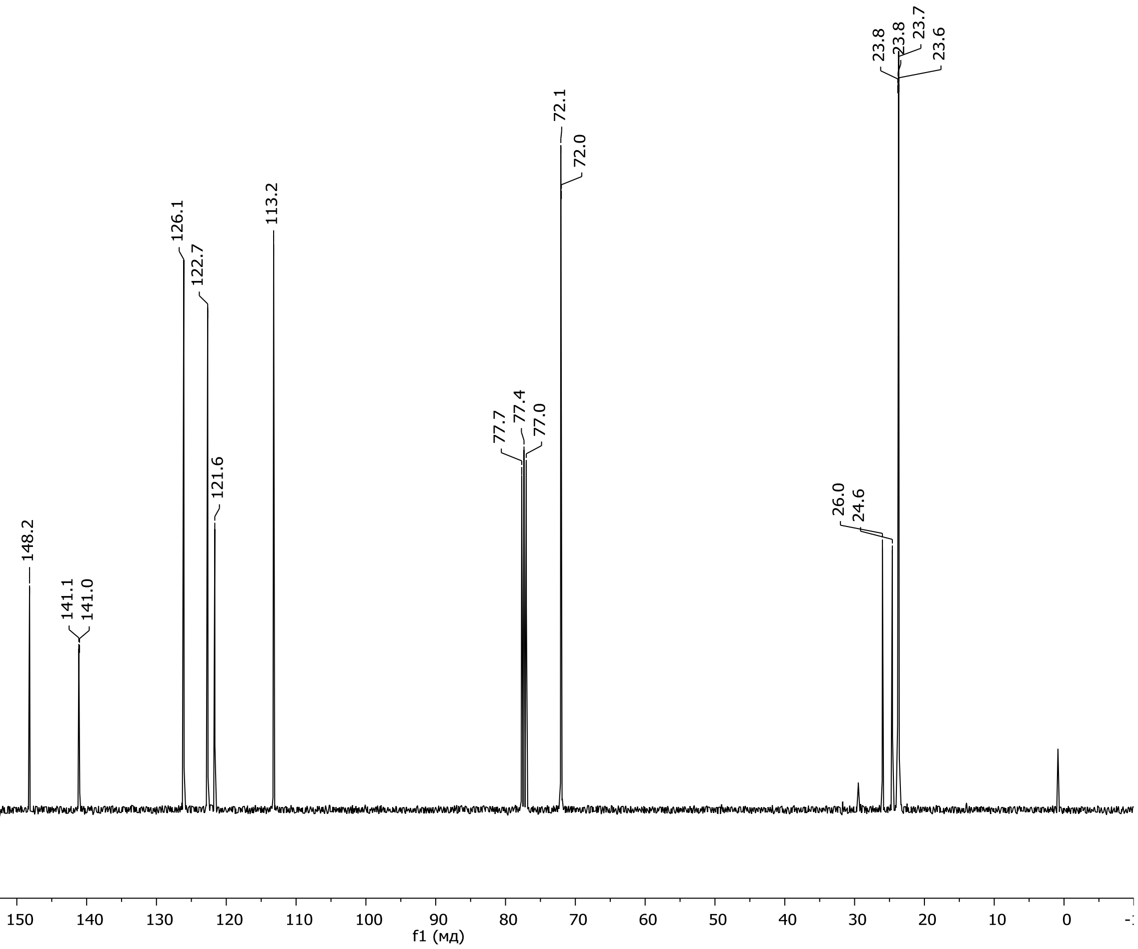

31P, 1D, 162.01 Hz
CDCl3

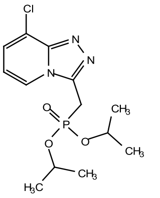

4b

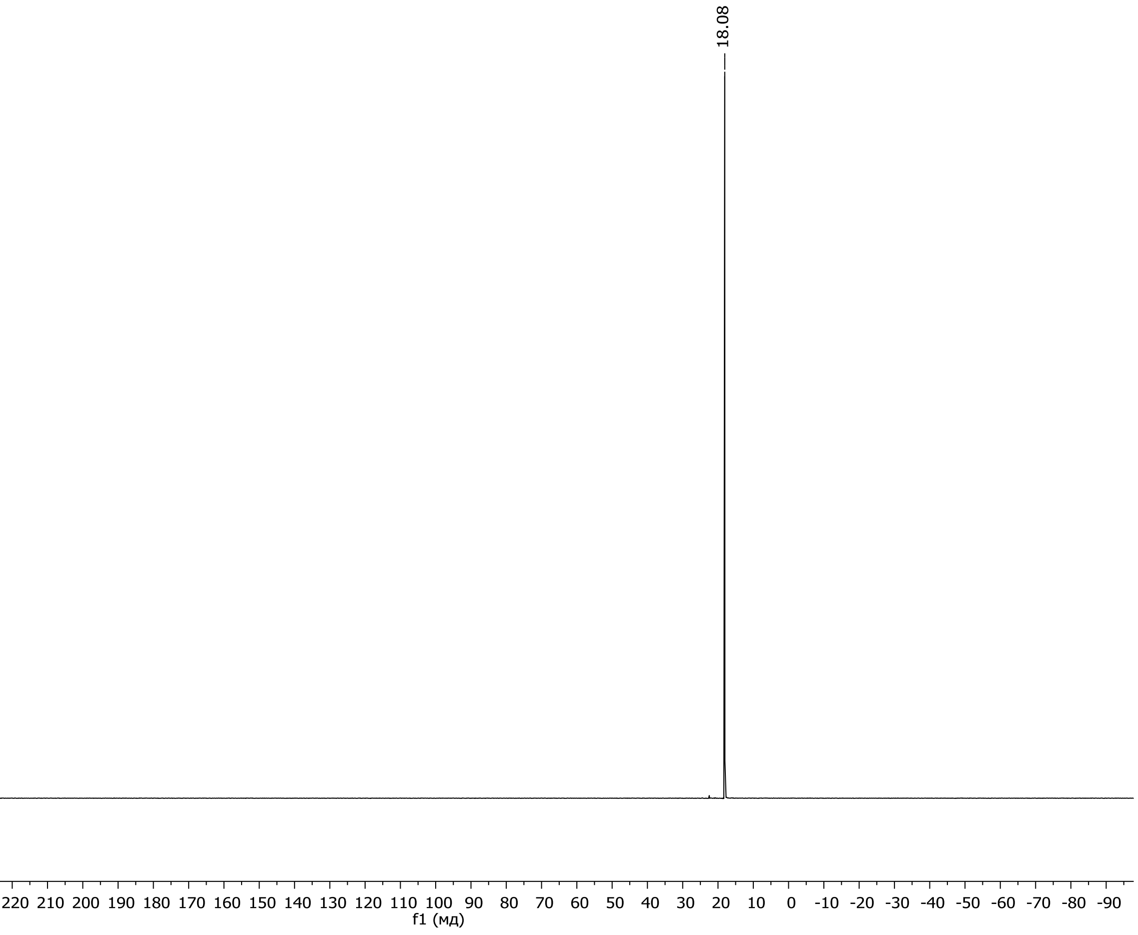

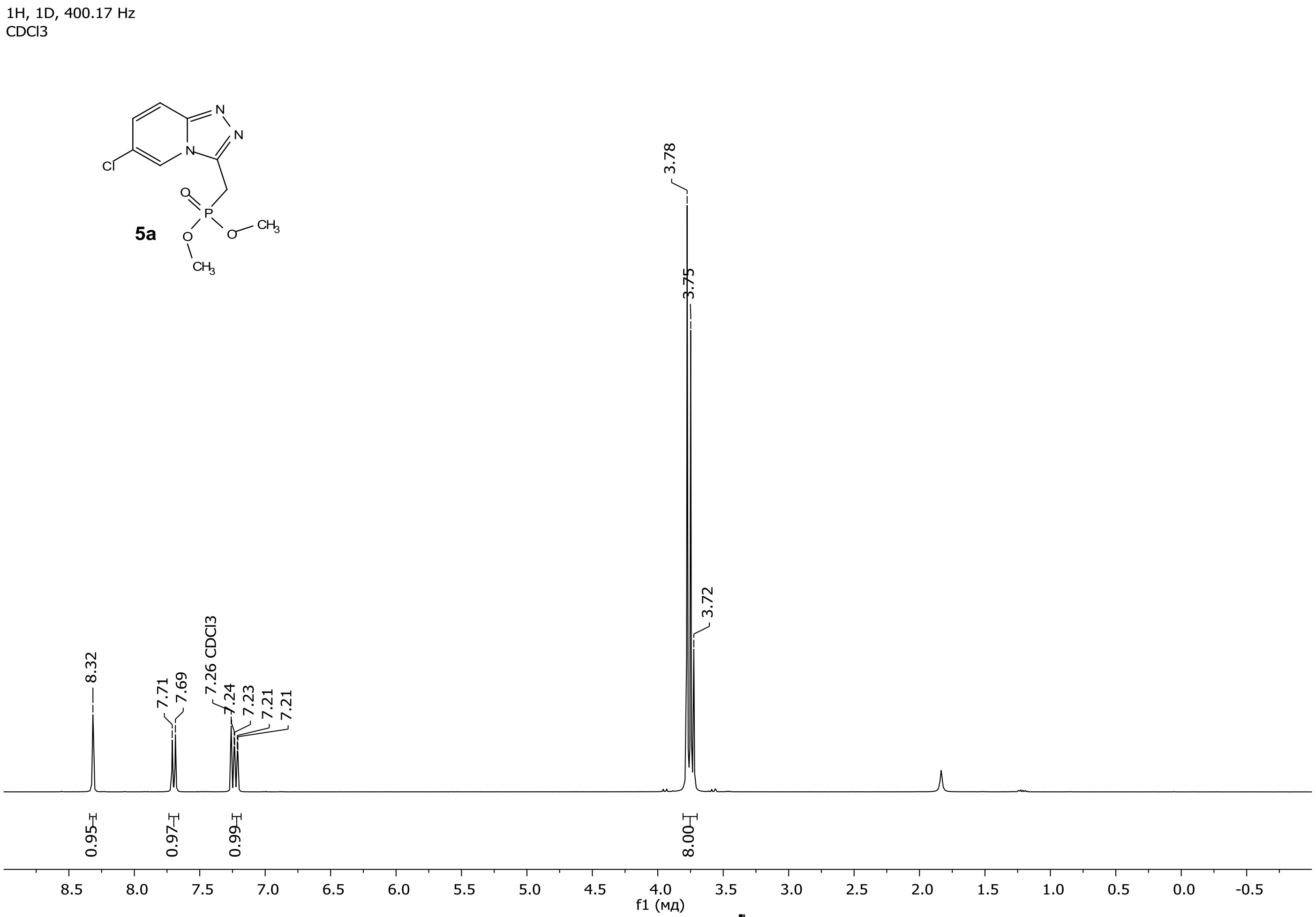
1H, 1D, 400.17 Hz
CDCl3
5a
Cl
N
CH3
3.78
3.75
3.72
8.32
7.71
7.69
7.26 CDCl3
7.24
7.23
7.21
7.21
0.95
0.97
0.99
8.00
8.5 8.0 7.5 7.0 6.5 6.0 5.5 5.0 4.5 4.0 3.5 3.0 2.5 2.0 1.5 1.0 0.5 0.0 -0.5
f1 (мд)

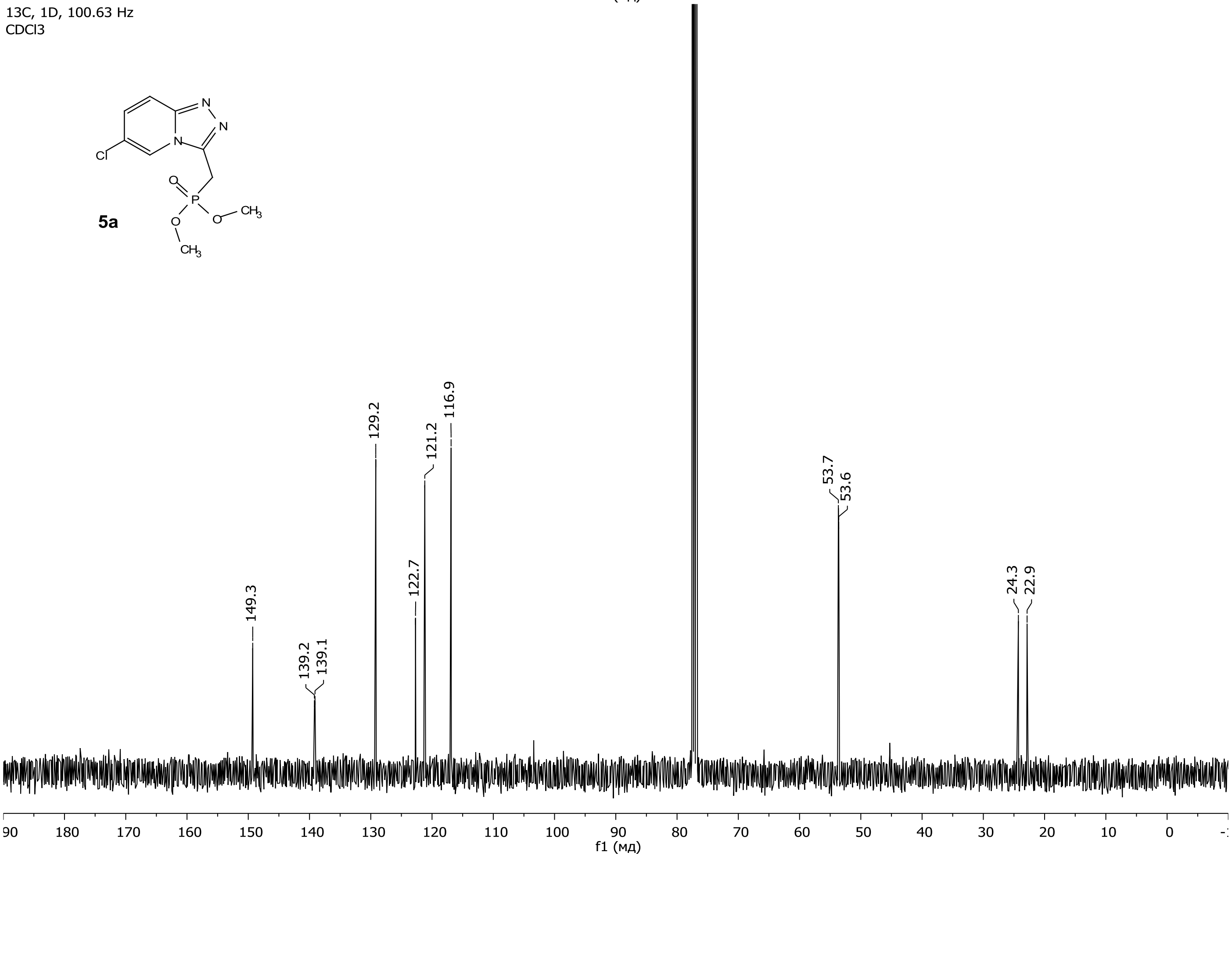
13C, 1D, 100.63 Hz
CDCl3
5a
Cl
N
CH3
149.3
139.2
139.1
129.2
122.7
121.2
116.9
53.7
53.6
24.3
22.9
180 170 160 150 140 130 120 110 100 90 80 70 60 50 40 30 20 10 0
f1 (мд)

31P, 1D, 162.01 Hz
CDCl3

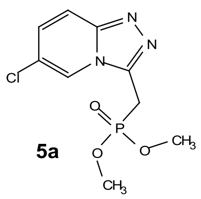

5a

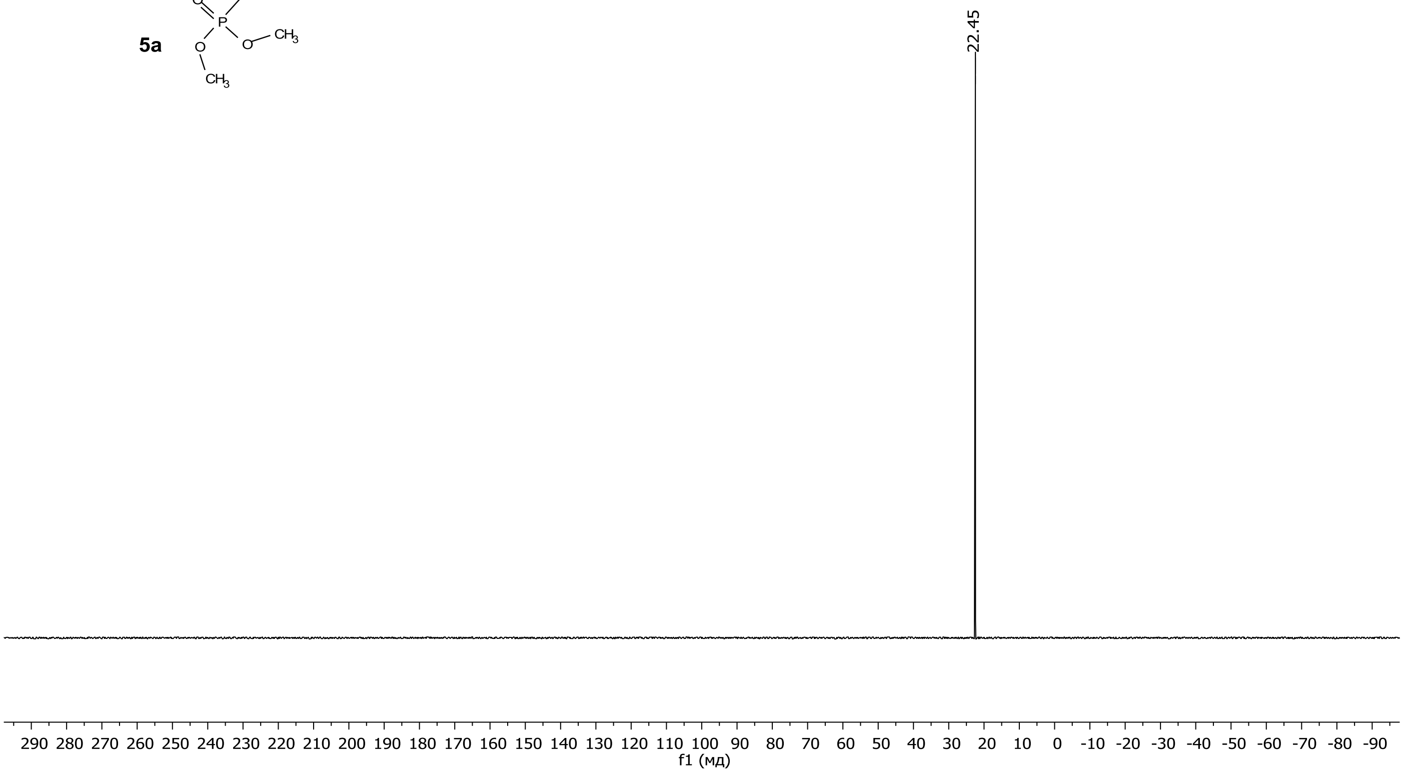

1H, 1D, 400.17 Hz
CDCl3

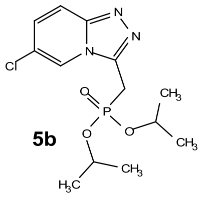

5b

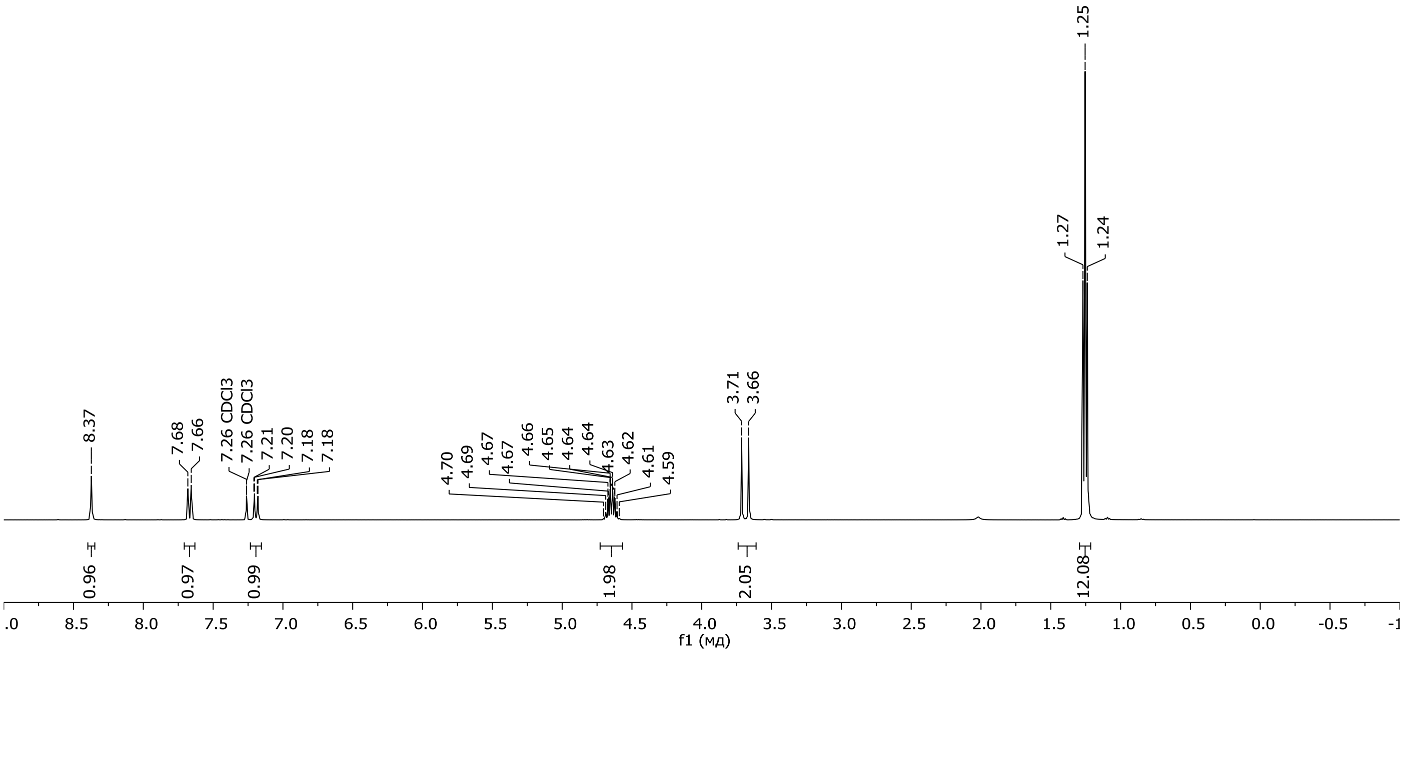

13C, 1D, 100.63 Hz
CDCl3

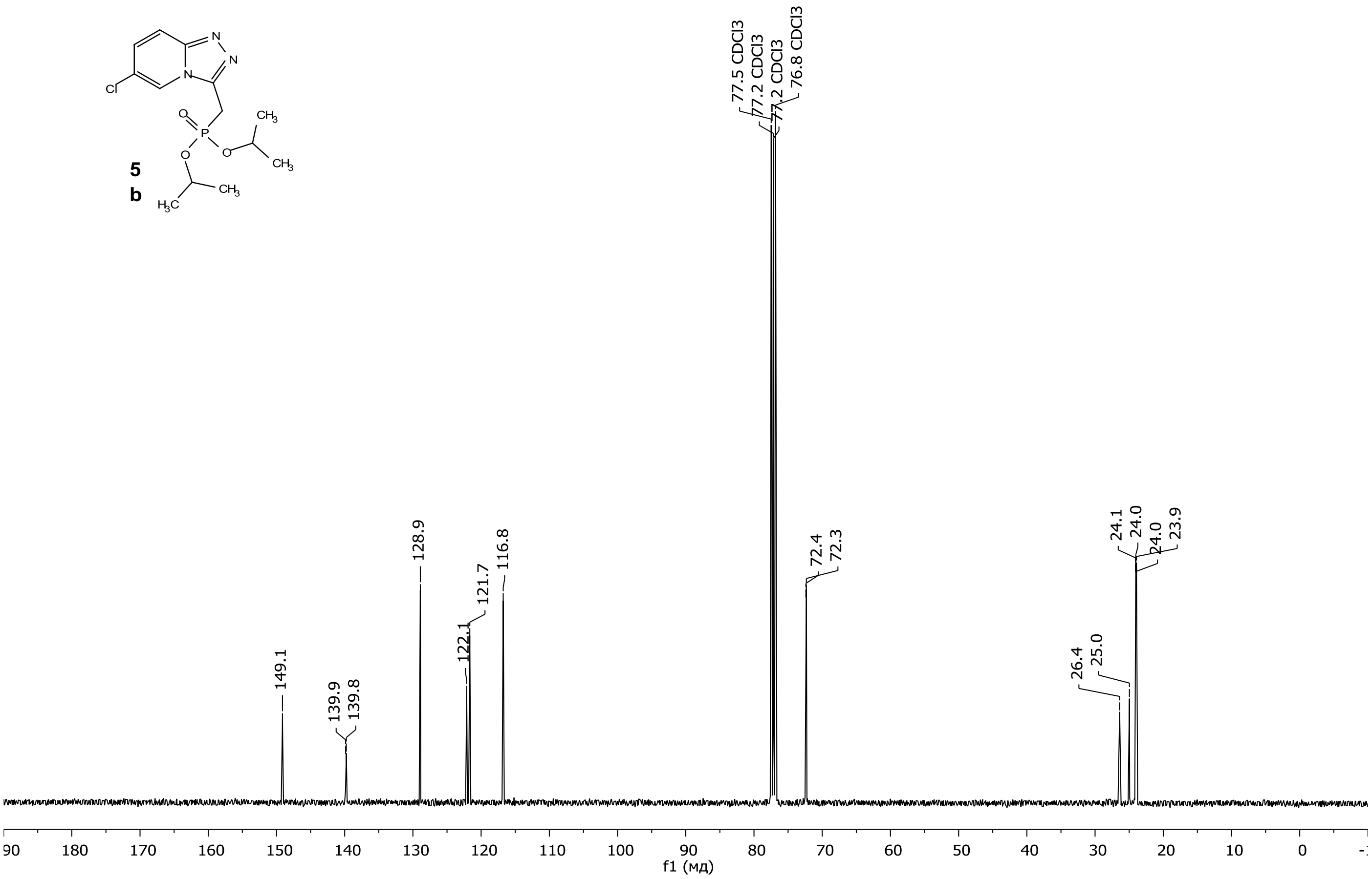

31P, 1D, 162.01 Hz
CDCl3

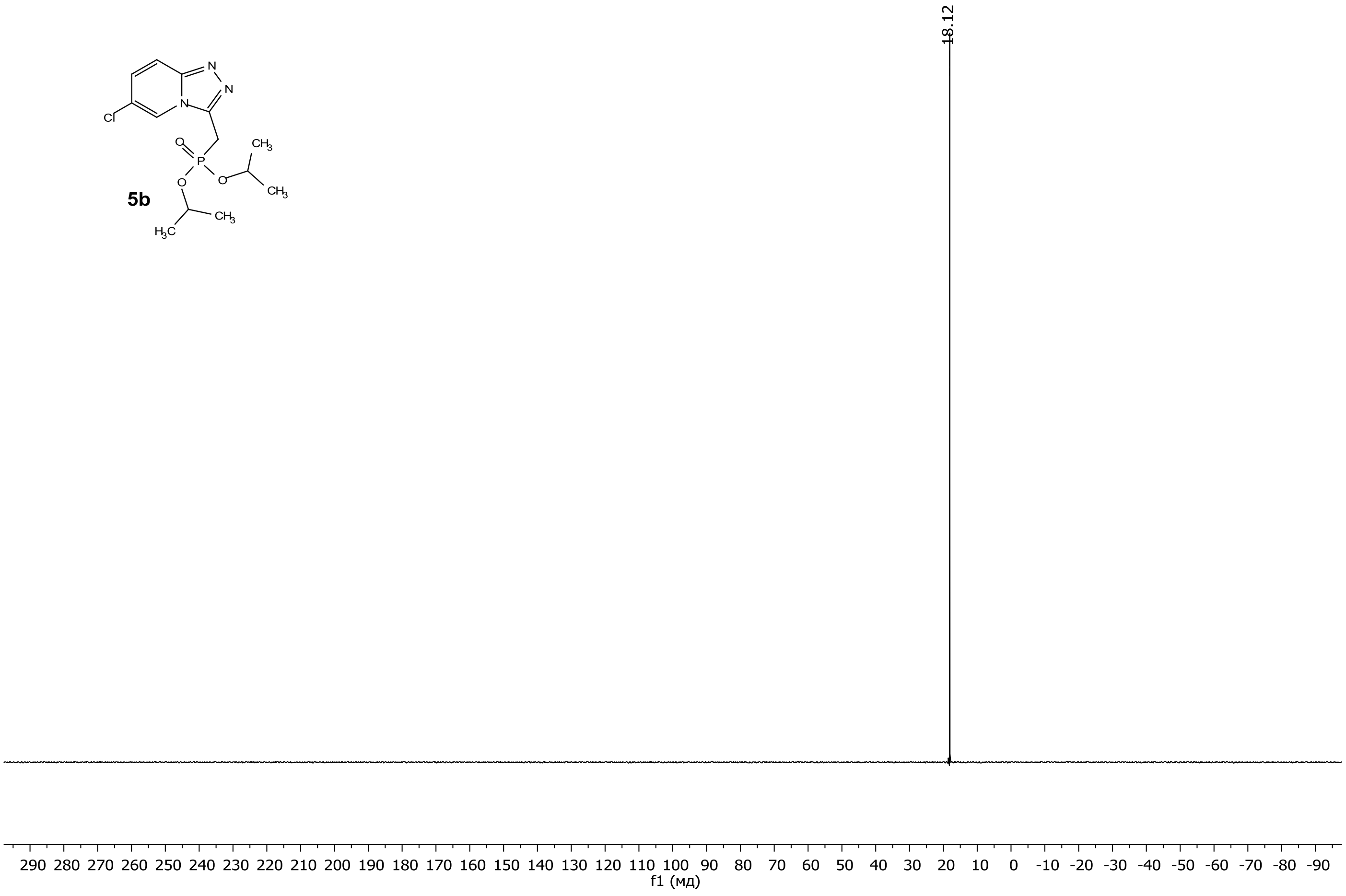

1H, 1D, 400.17 Hz
CDCl3

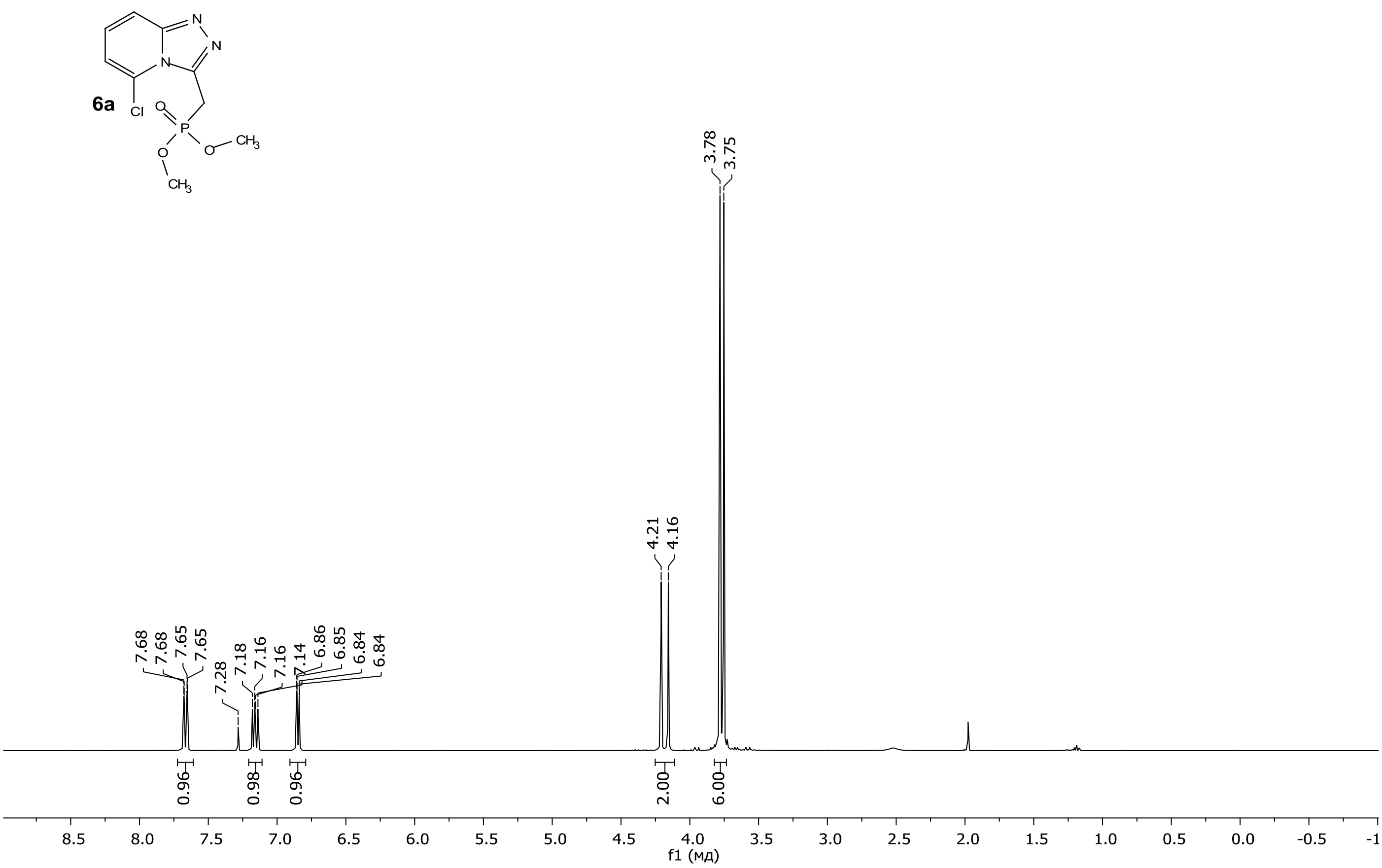

13C, 1D, 100.63 Hz
CDCl3

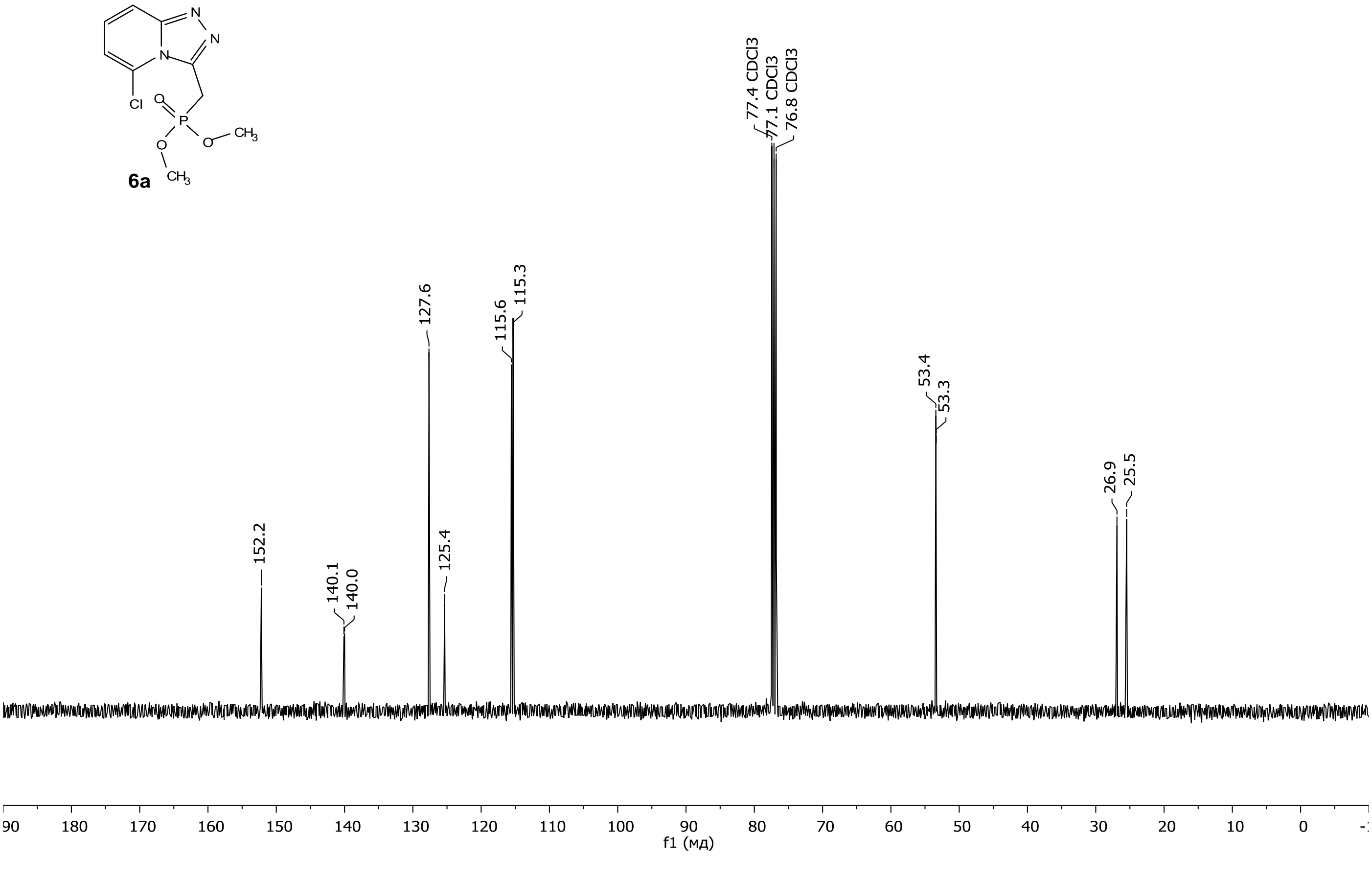

31P, 1D, 162.01 Hz
CDCl3

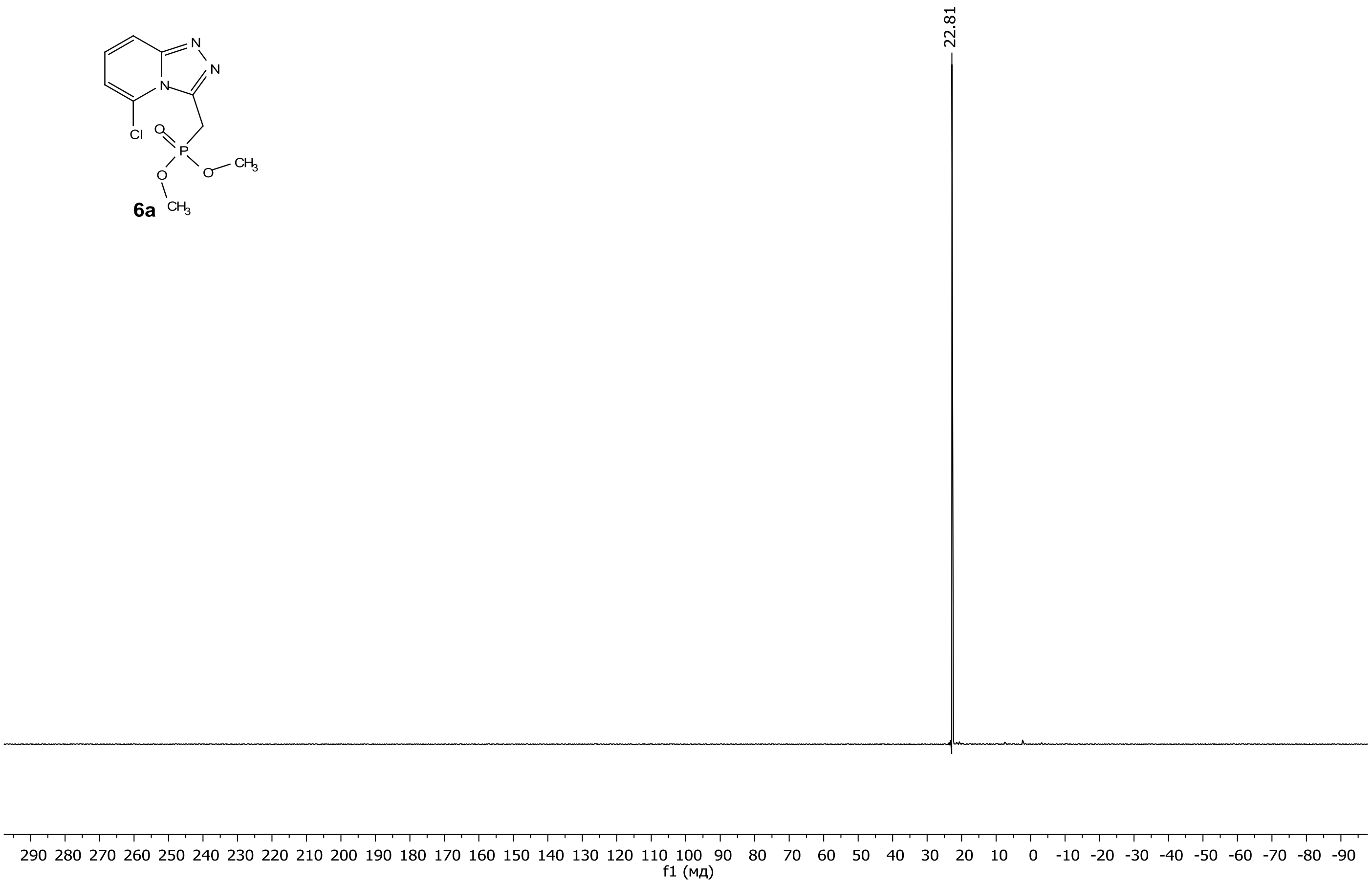

1H, 1D, 400.17 Hz
CDCl3

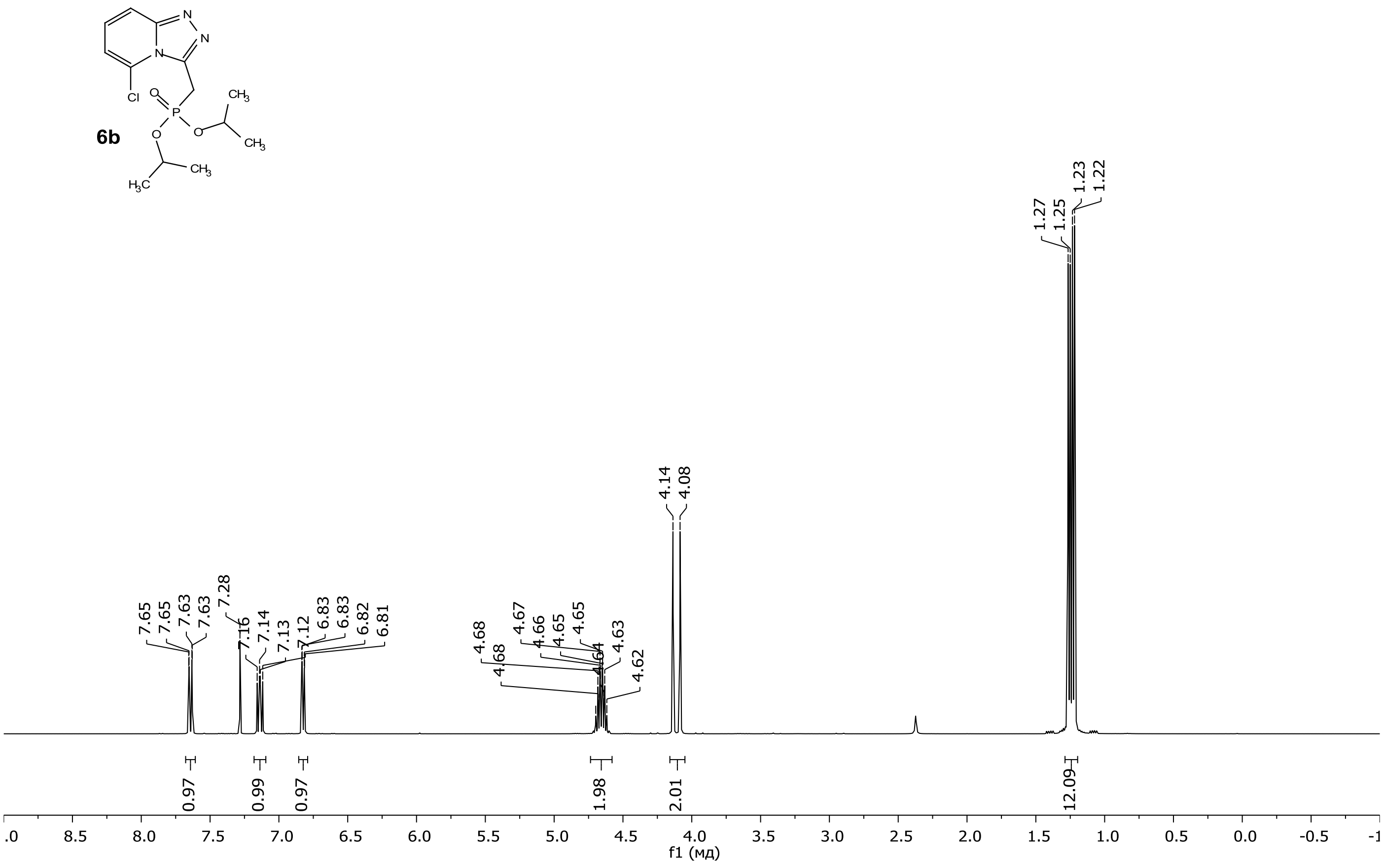

13C, 1D, 100.63 Hz
CDCl3

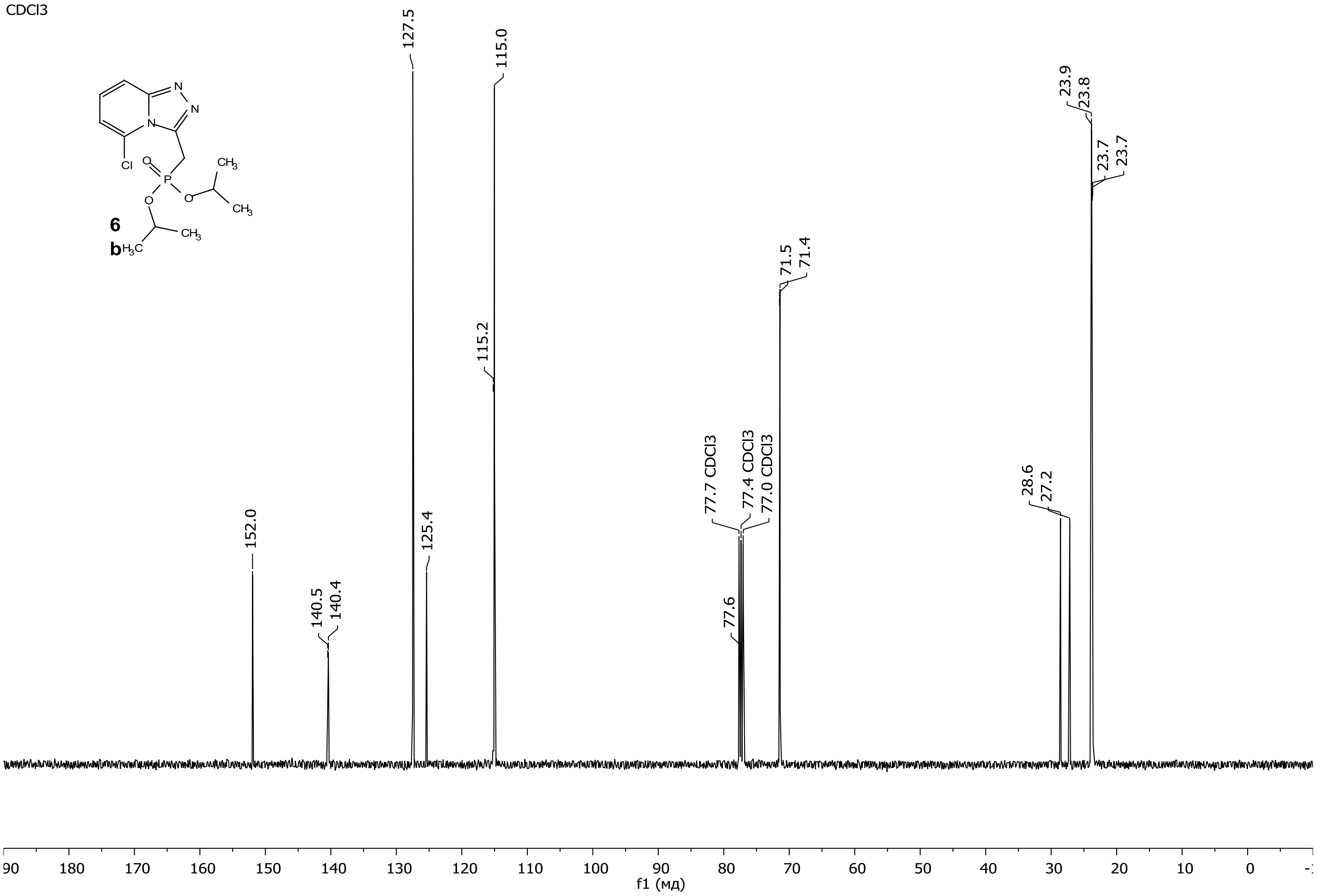

31P, 1D, 162.01 Hz
CDCl3

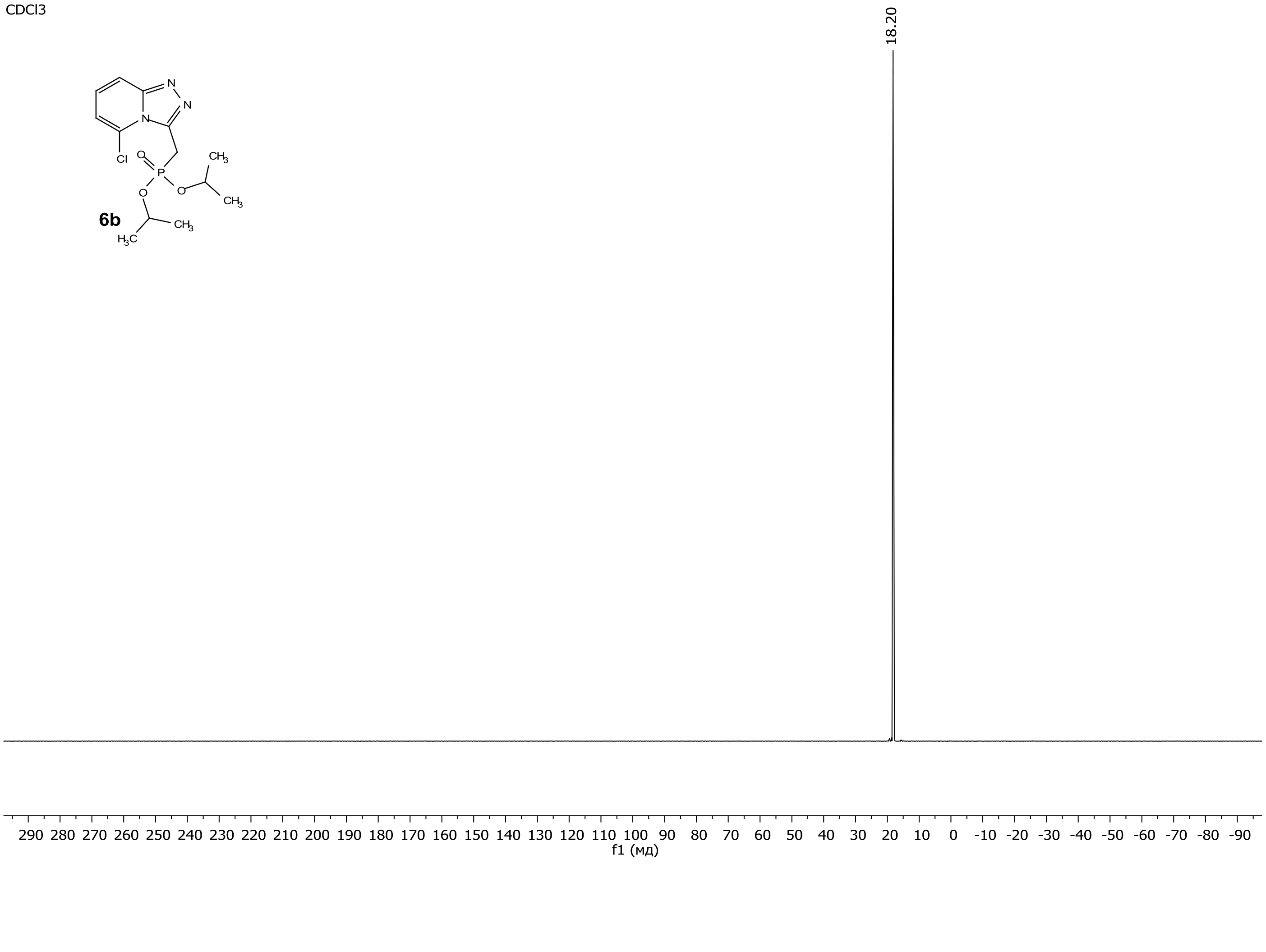

1H, 1D, 400.17 Hz
CDCl3

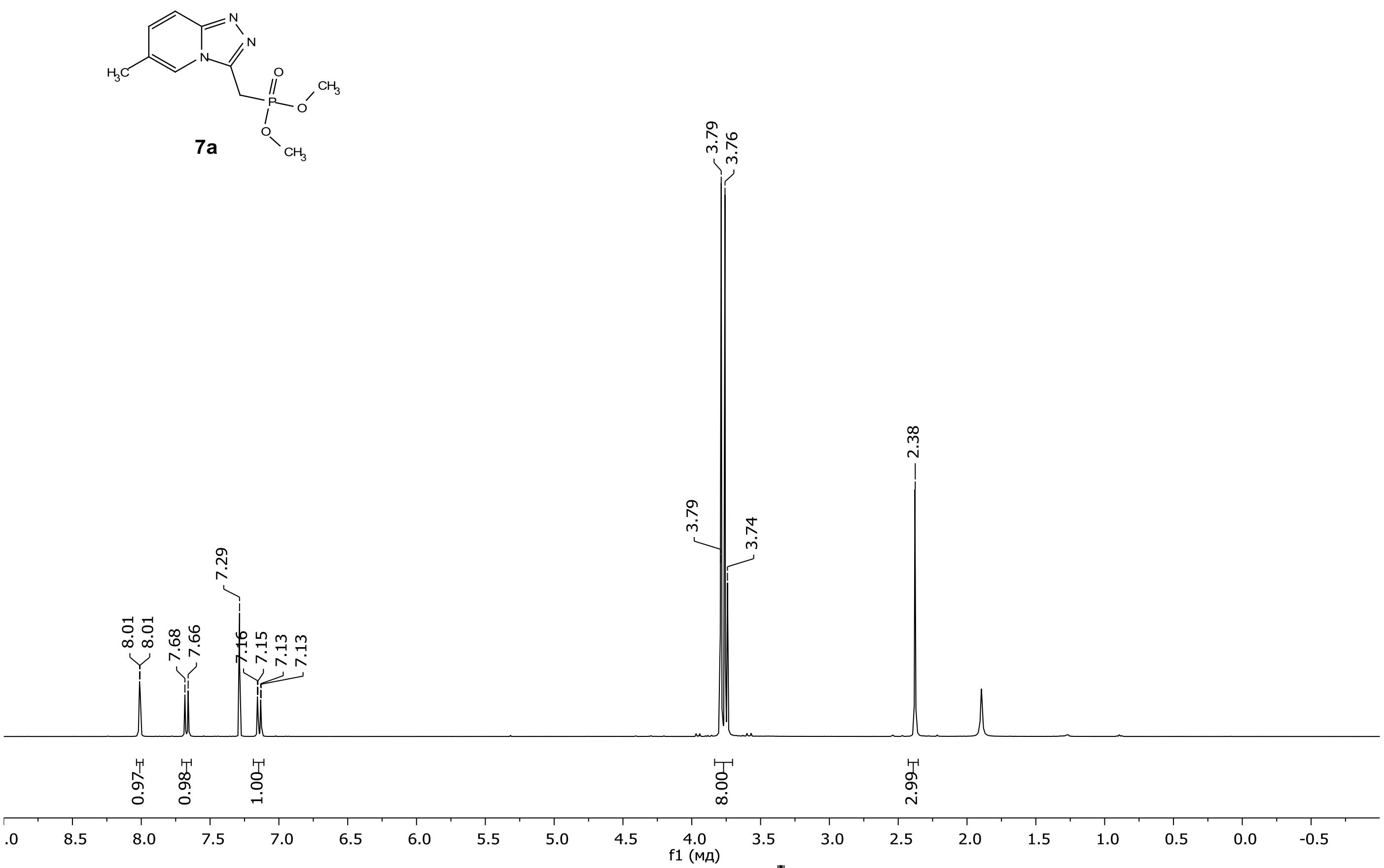

13C, 1D, 100.63 Hz
CDCl3

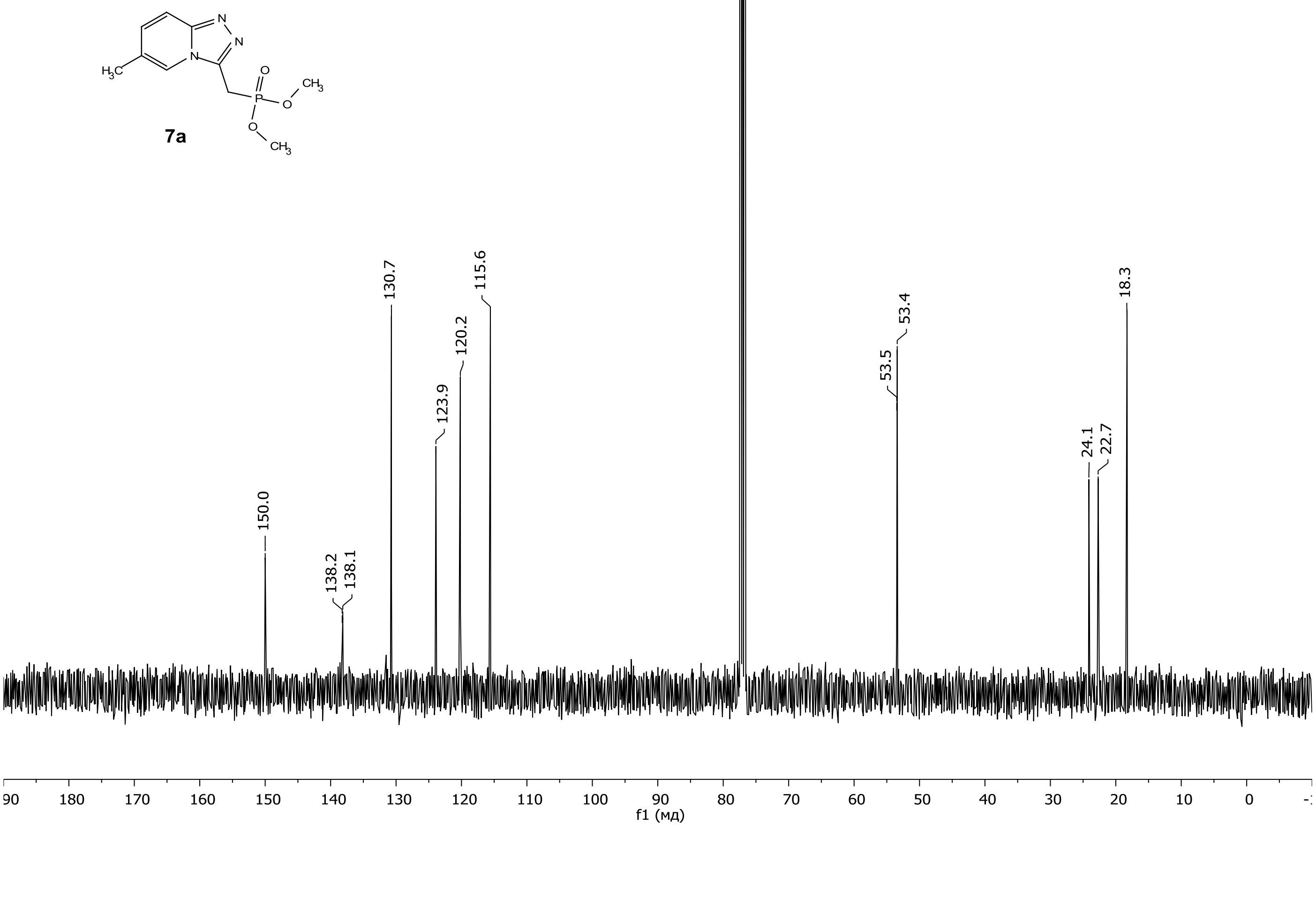

31P, 1D, 162.01 Hz
CDCl3

7a

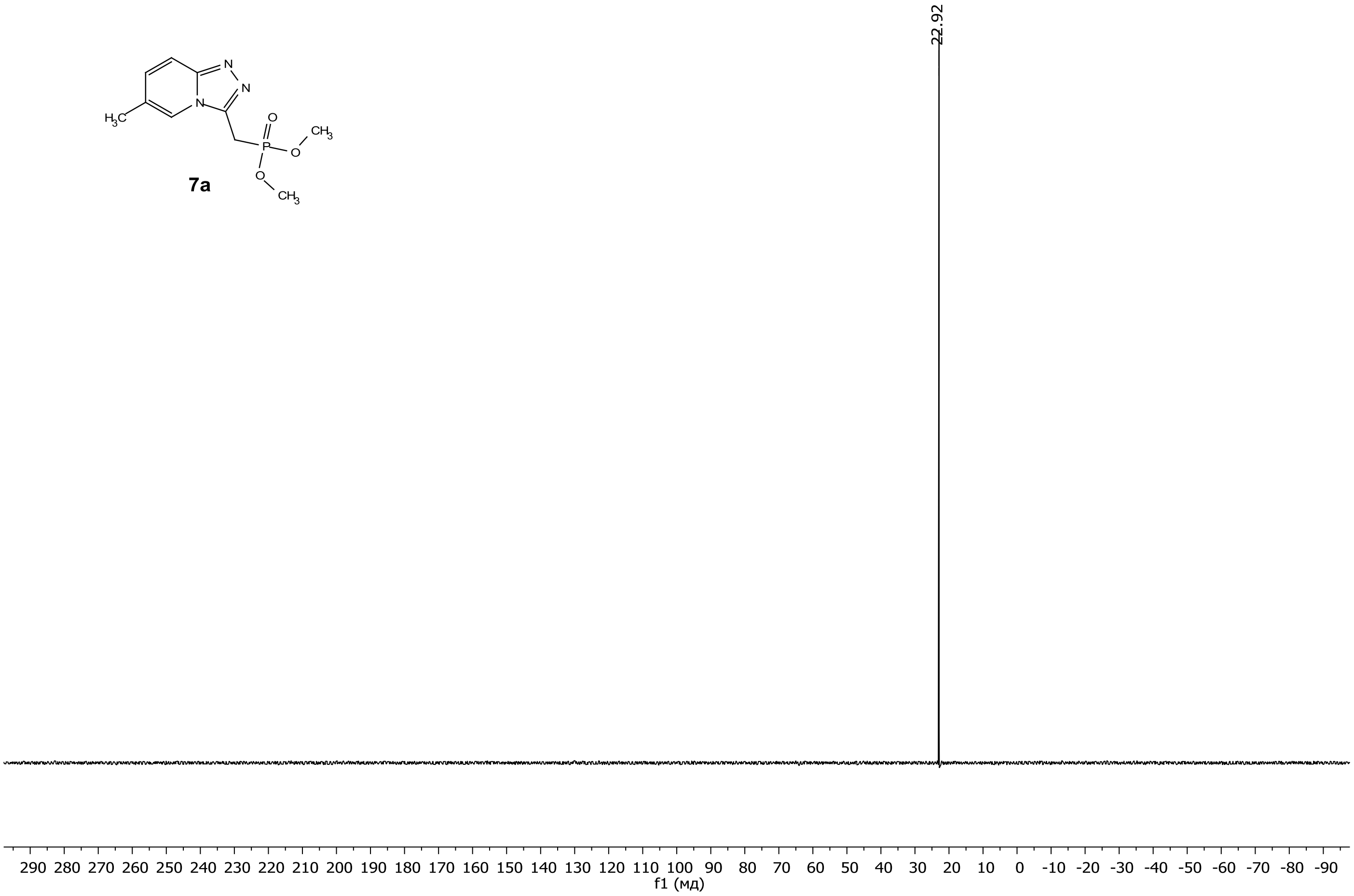

1H, 1D, 400.17 Hz
CDCl3

7b

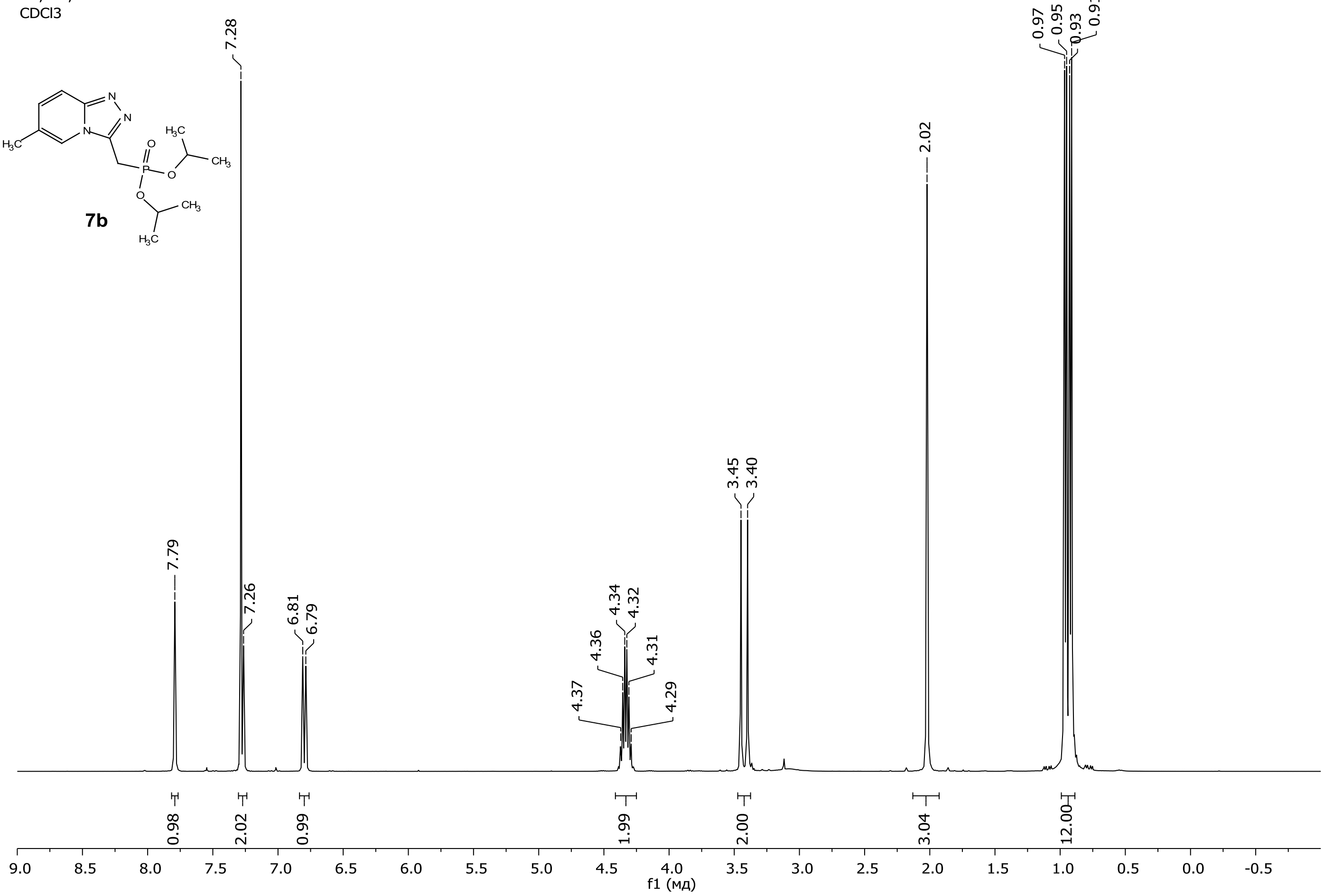

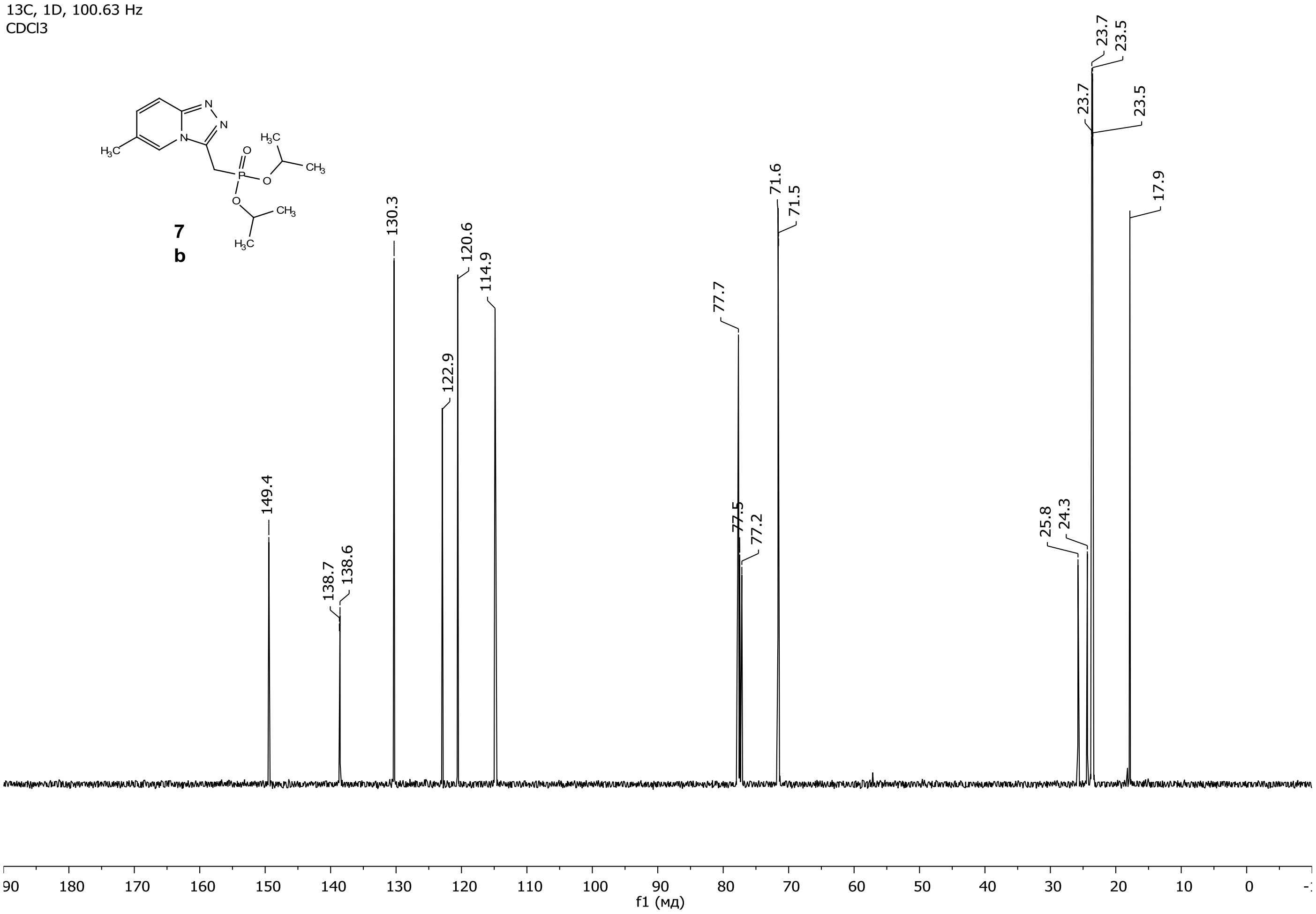
13C, 1D, 100.63 Hz
CDCl3
7
b
149.4
138.7
138.6
130.3
122.9
120.6
114.9
77.7
77.5
77.2
71.6
71.5
25.8
24.3
23.7
23.5
23.7
23.5
17.9
f1 (мд)

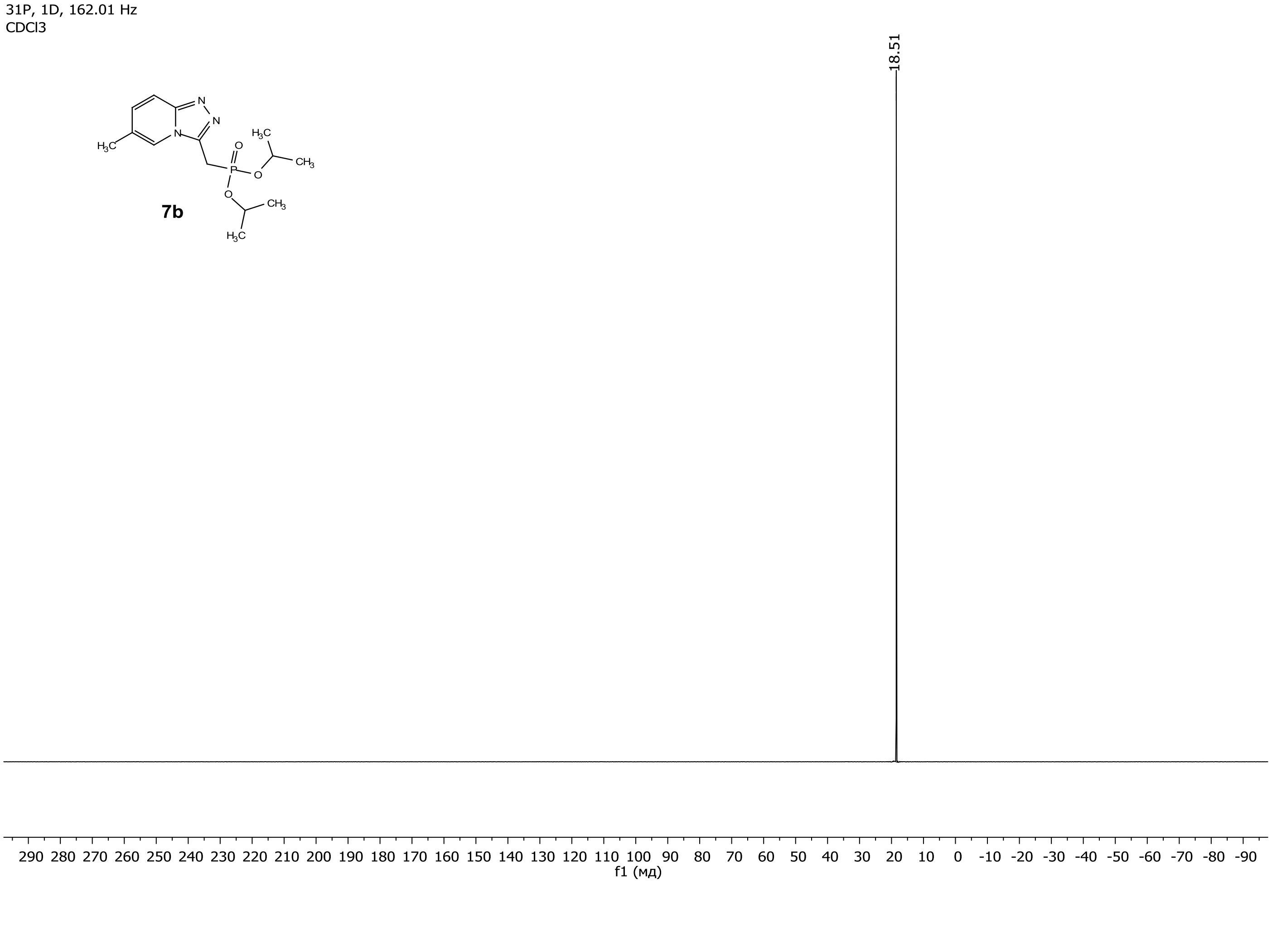
31P, 1D, 162.01 Hz
CDCl3
7b
18.51
f1 (мд)

1H, 1D, 400.17 Hz
CDCl3

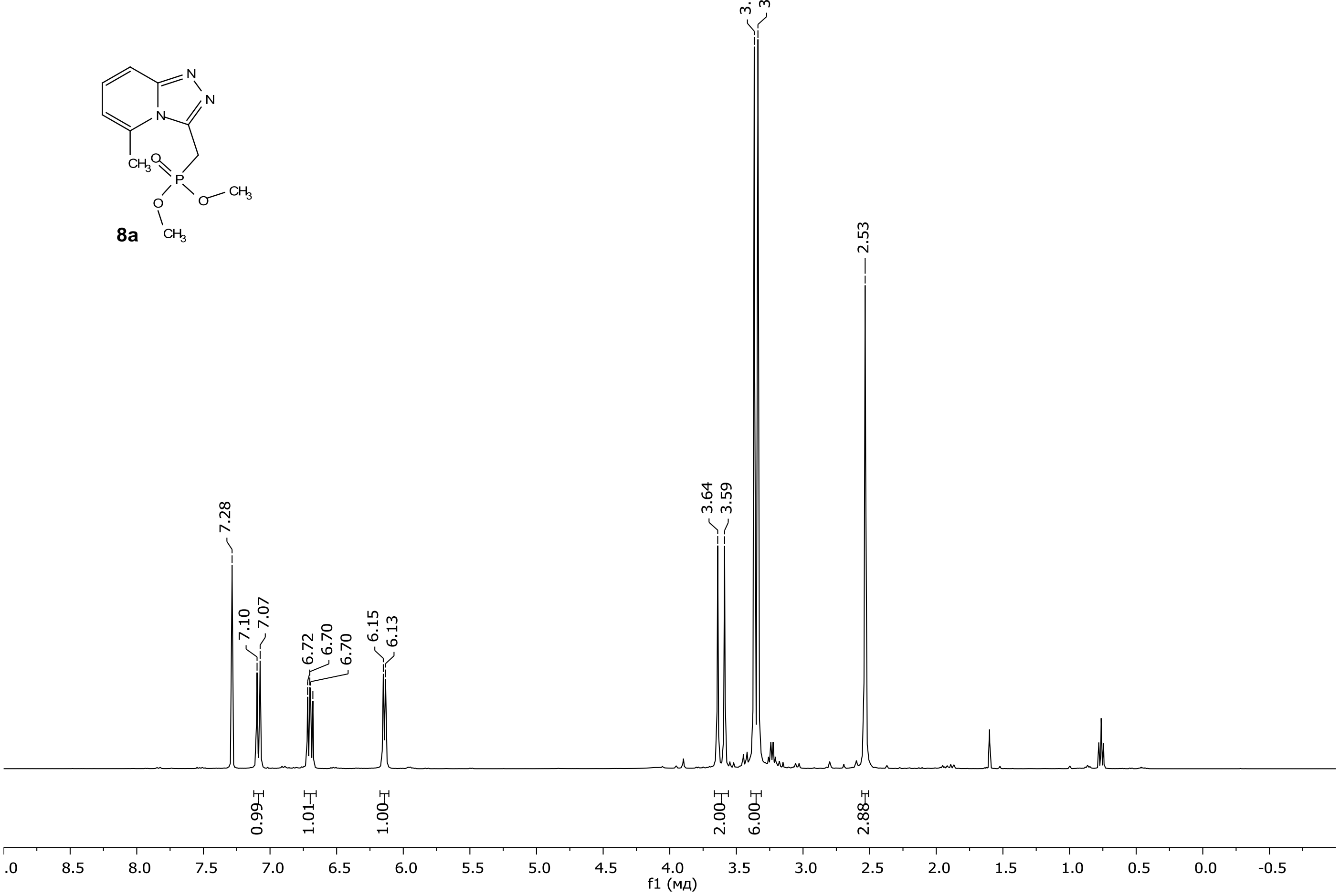

13C, 1D, 100.63 Hz
CDCl3

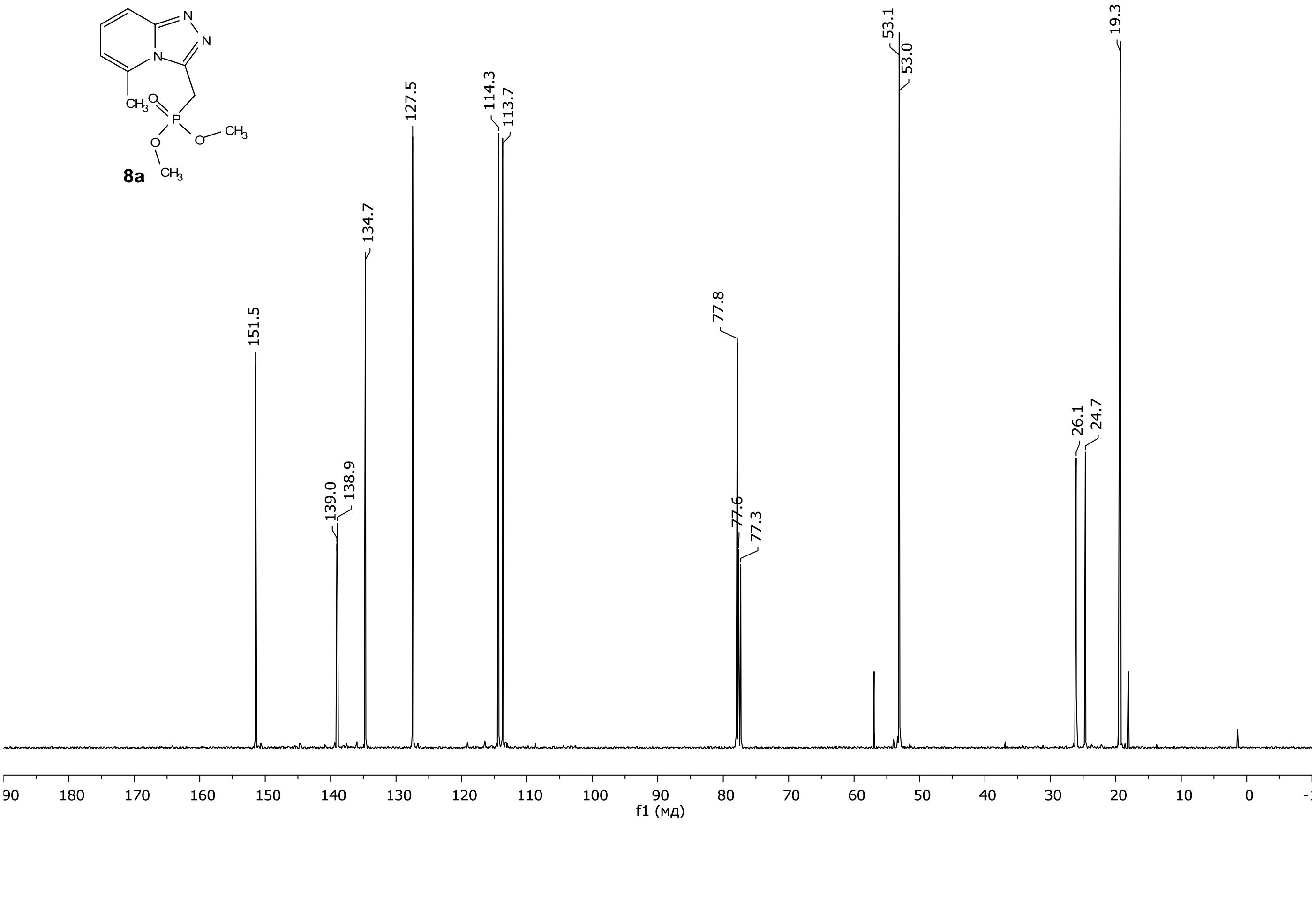

31P, 1D, 162.01 Hz
CDCl3

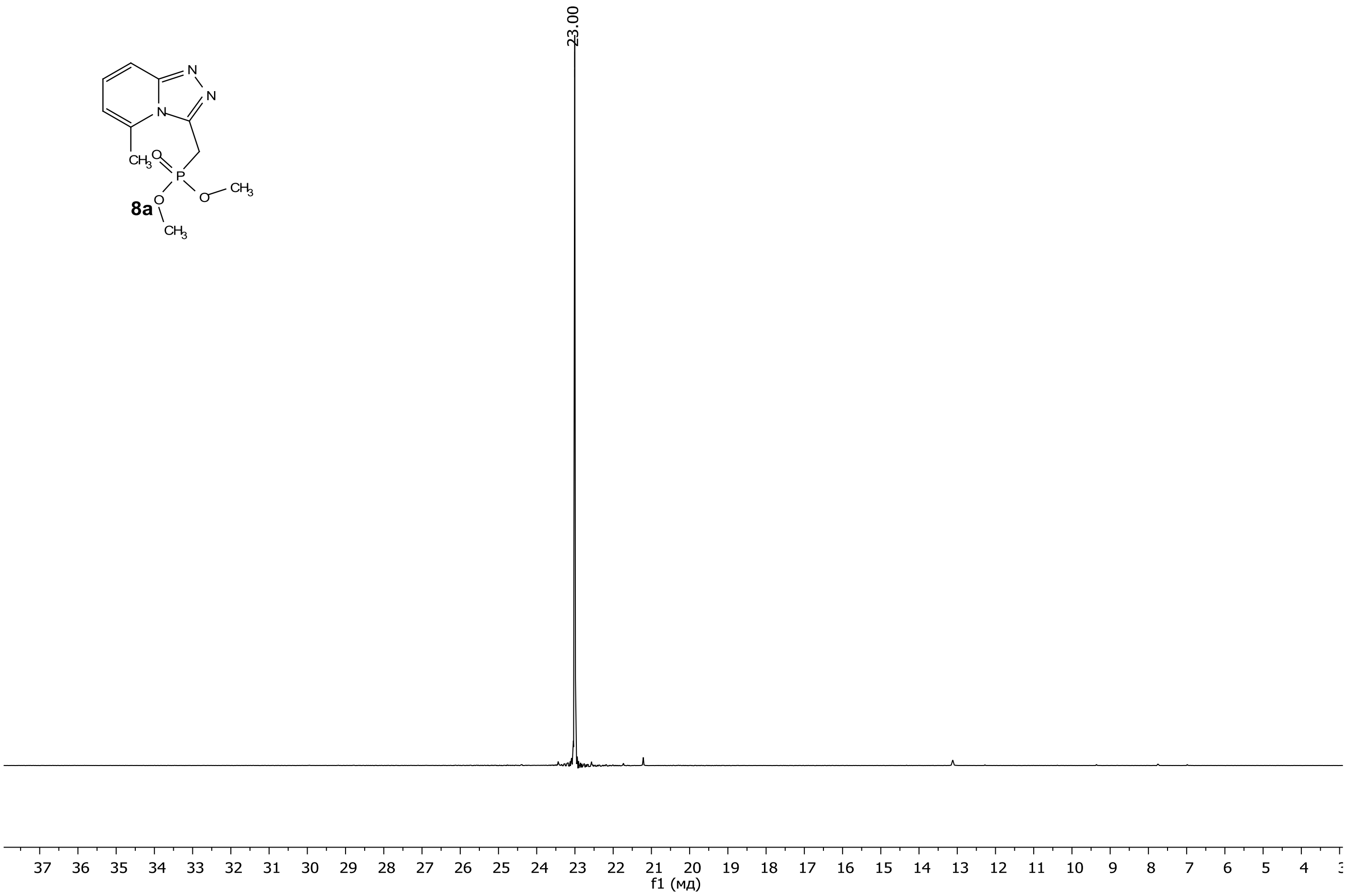

1H, 1D, 400.17 Hz
CDCl3

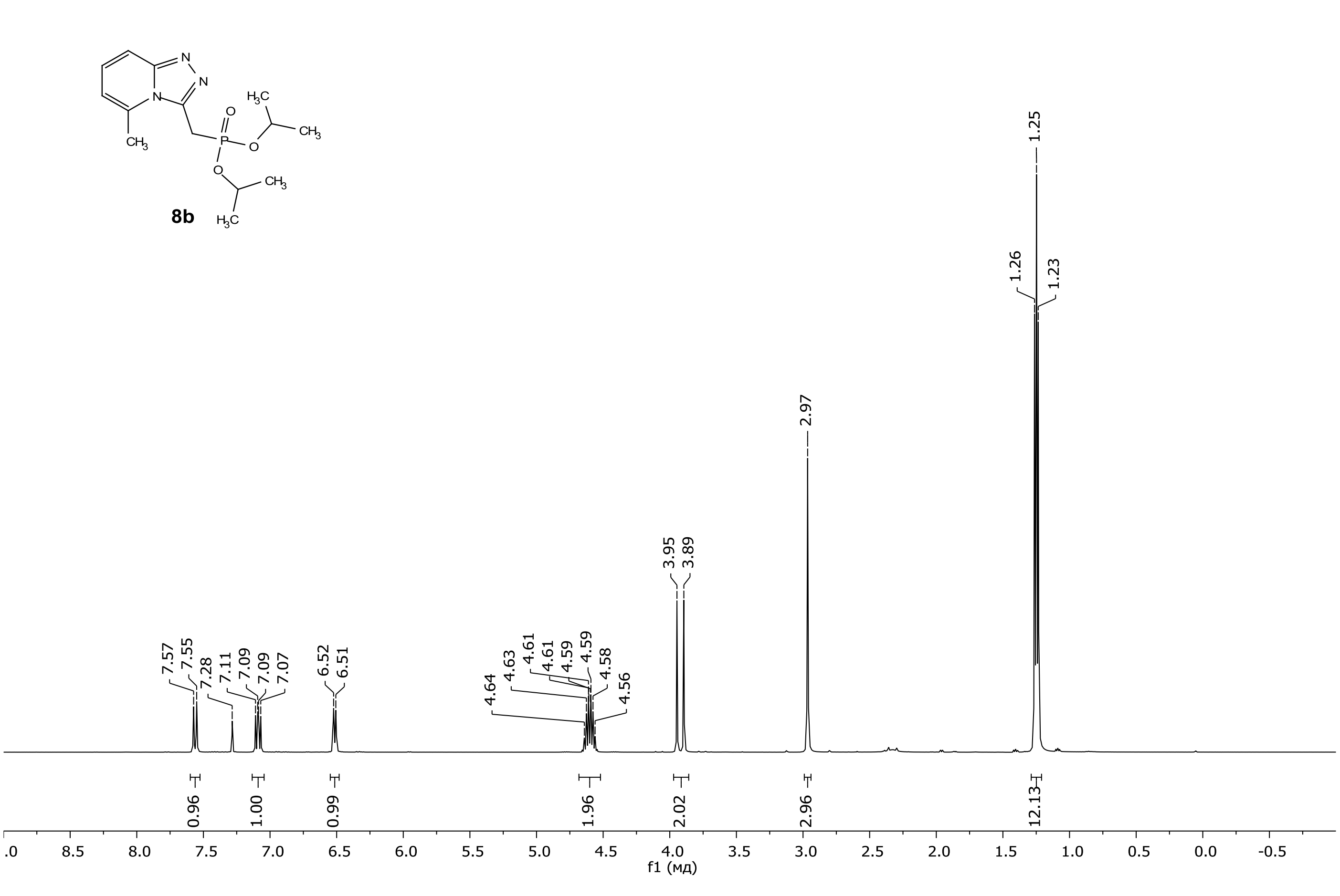

13C, 1D, 100.63 Hz
CDCl3

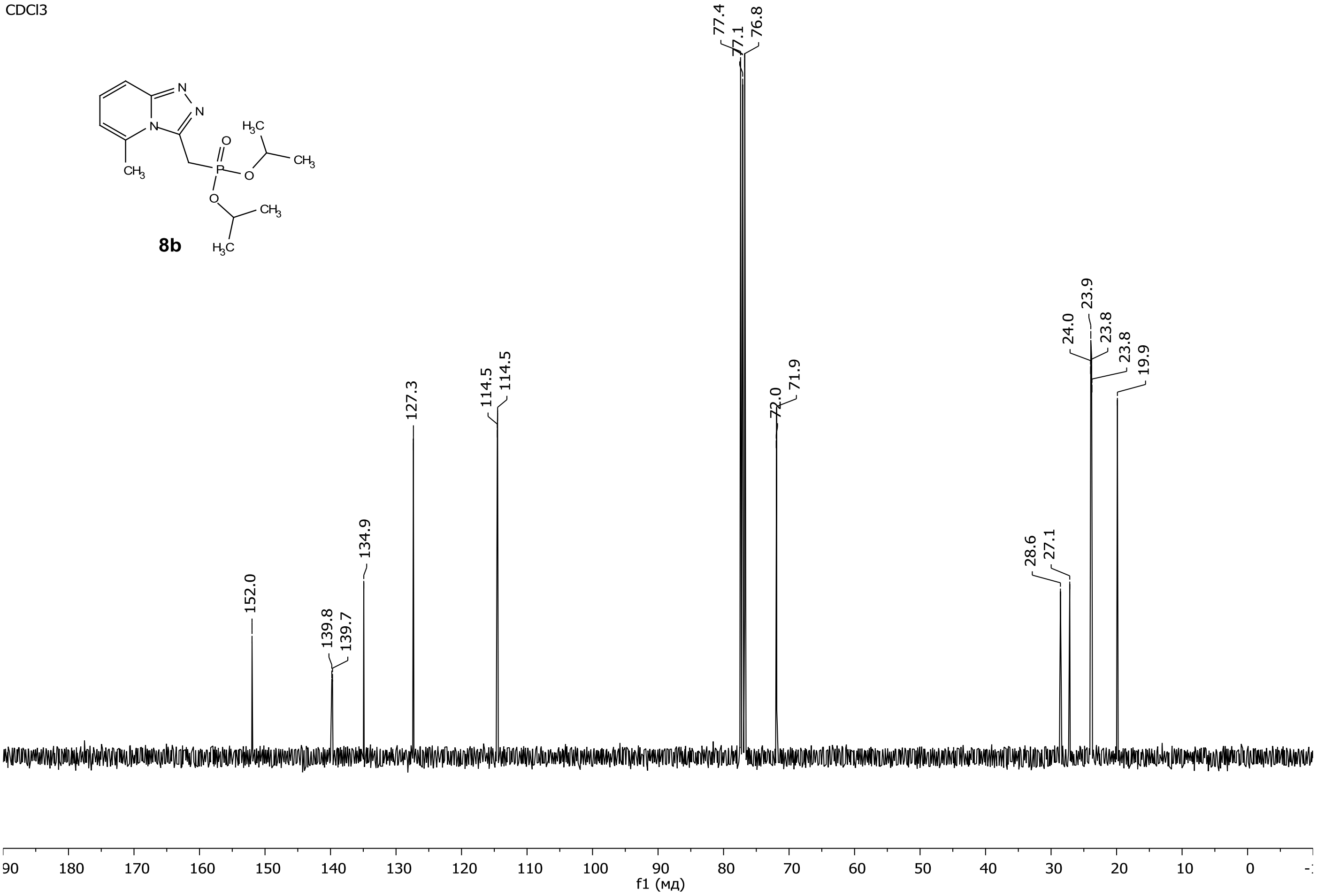

31P, 1D, 162.01 Hz
CDCl3

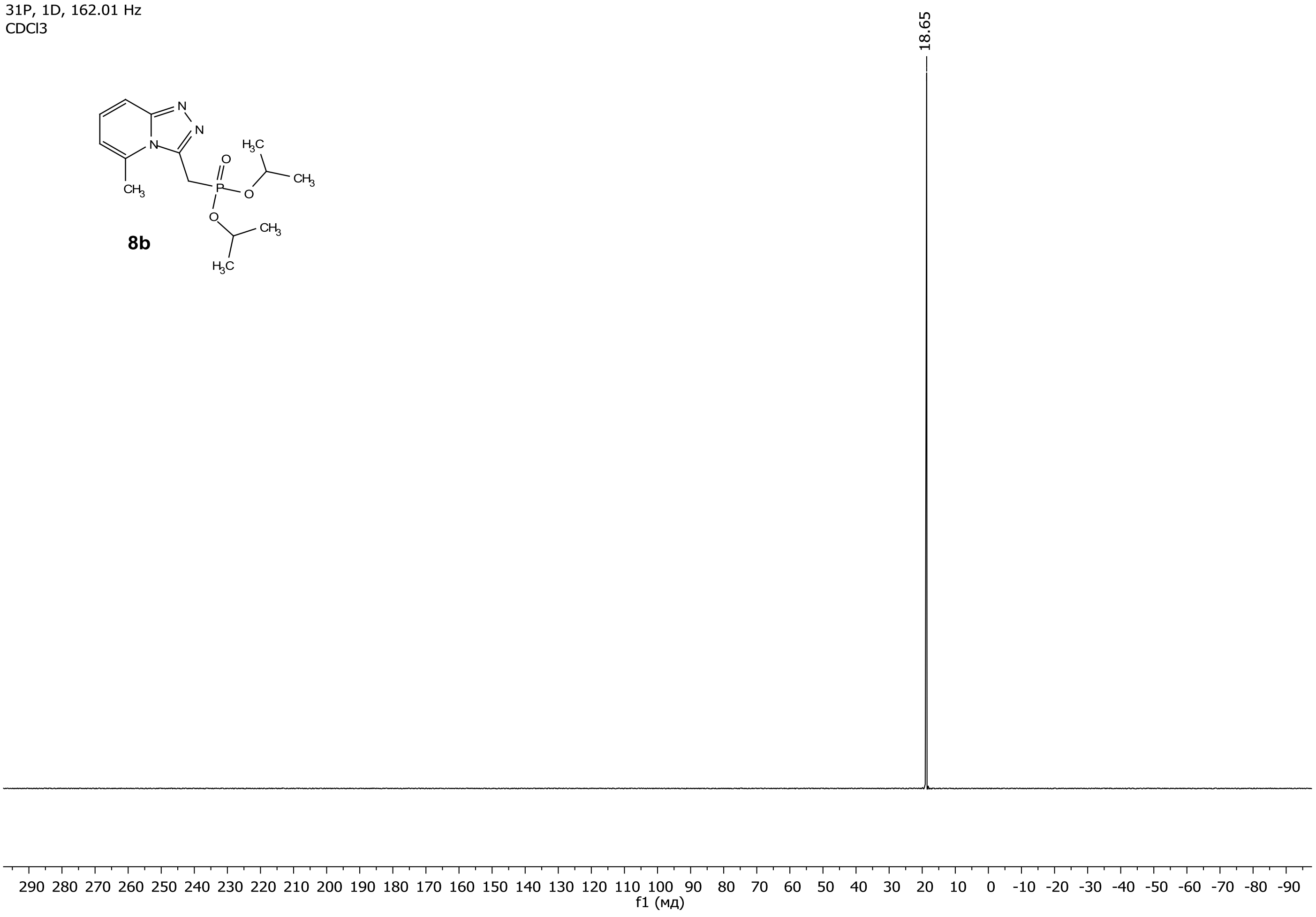

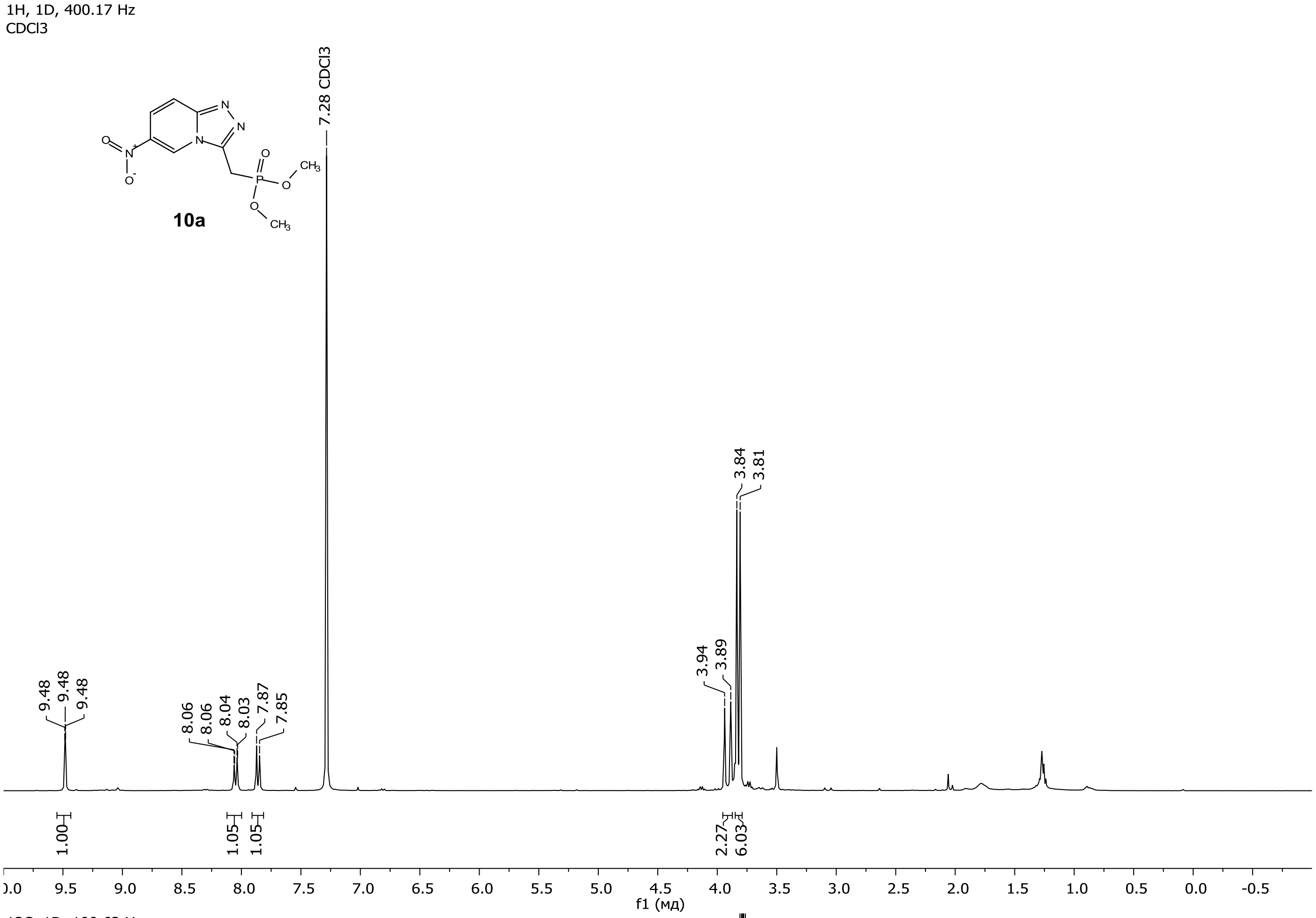
1H, 1D, 400.17 Hz
CDCl3
10a
7.28 CDCl3
9.48 9.48 9.48
8.06 8.06 8.04 8.03 7.87 7.85
3.94 3.89
3.84 3.81
1.00
1.05 1.05
2.27 6.03
f1 (мд)

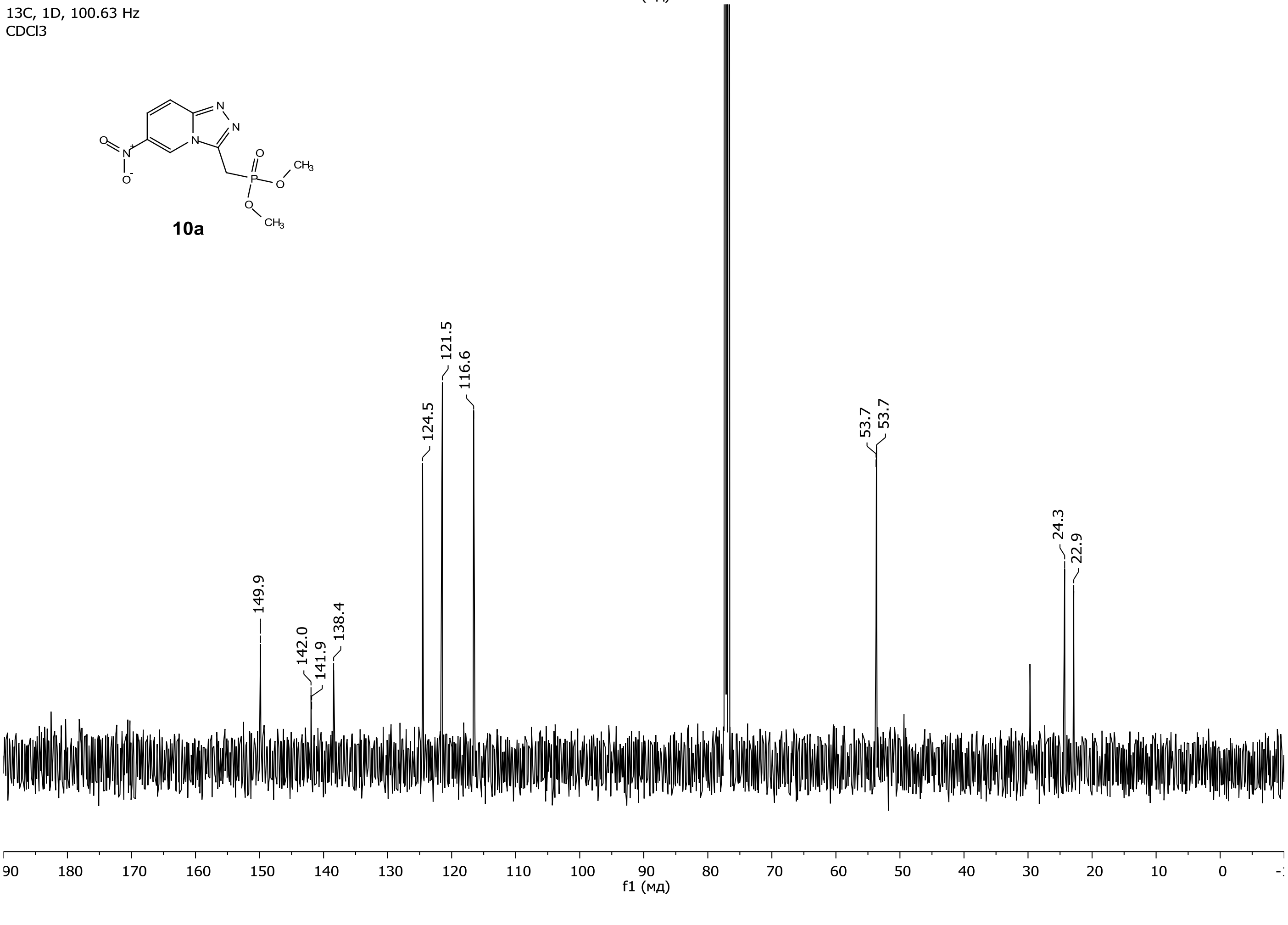
13C, 1D, 100.63 Hz
CDCl3
10a
149.9
142.0 141.9 138.4
124.5
121.5 116.6
53.7 53.7
24.3 22.9
f1 (мд)

31P, 1D, 162.01 Hz
CDCl3

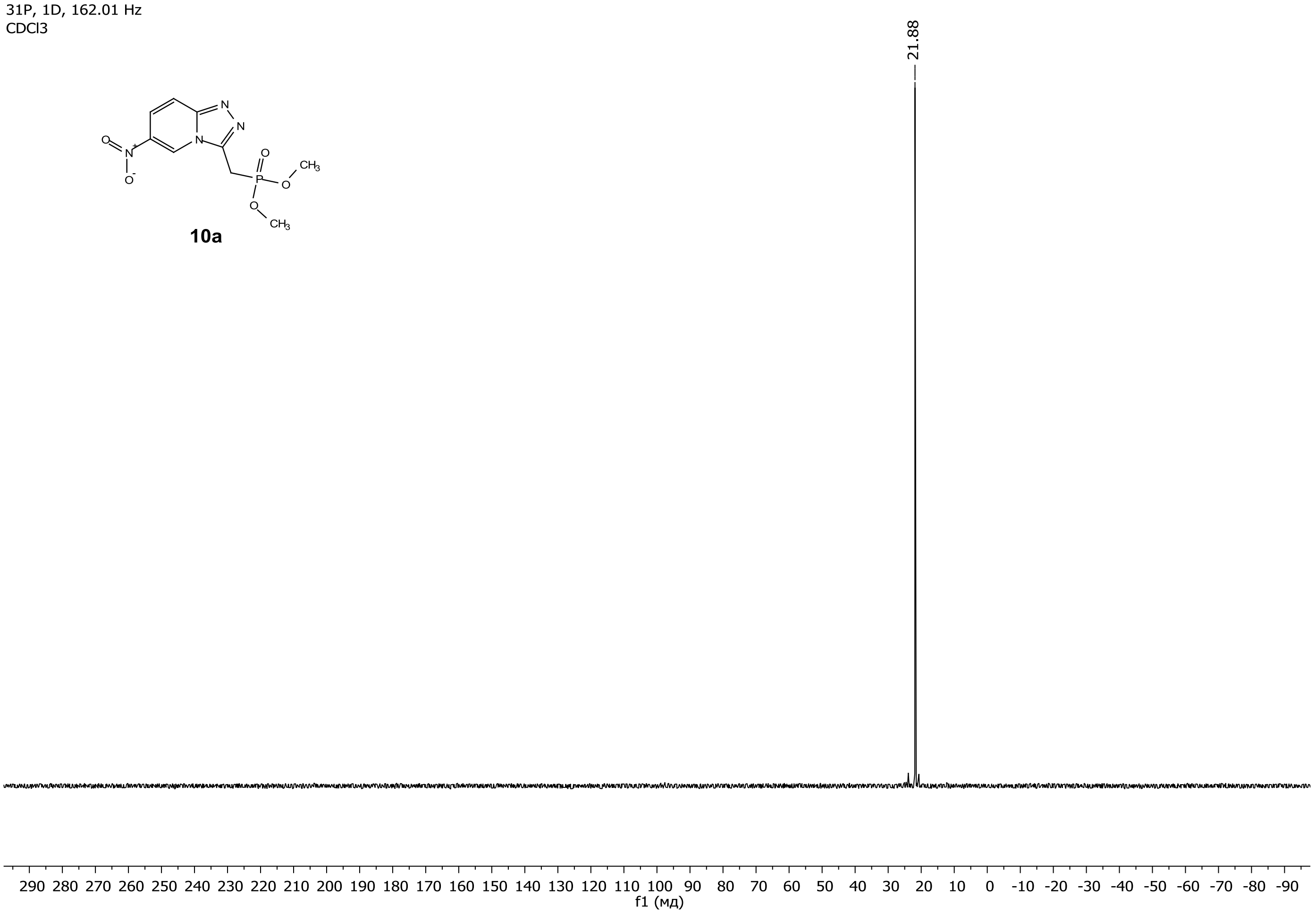

1H, 1D, 400.17 Hz
CDCl3

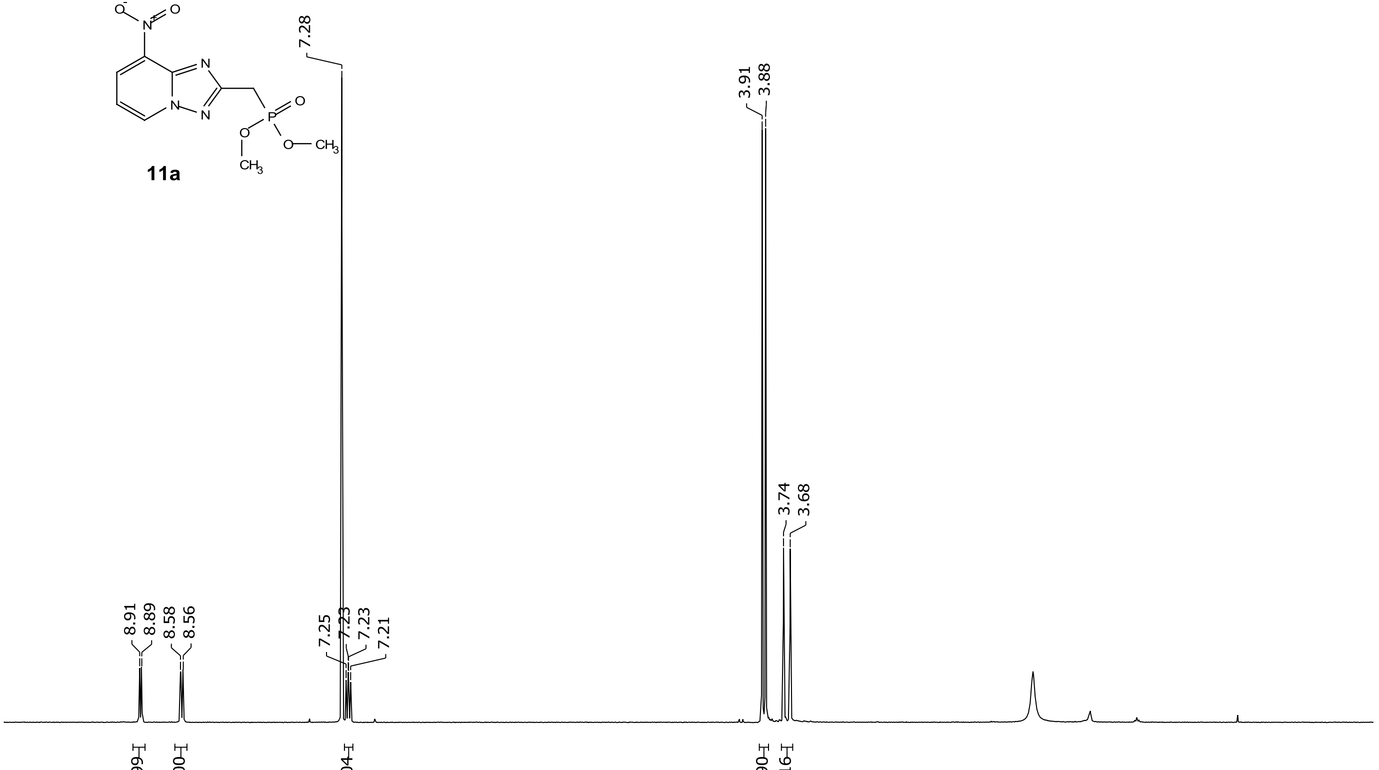

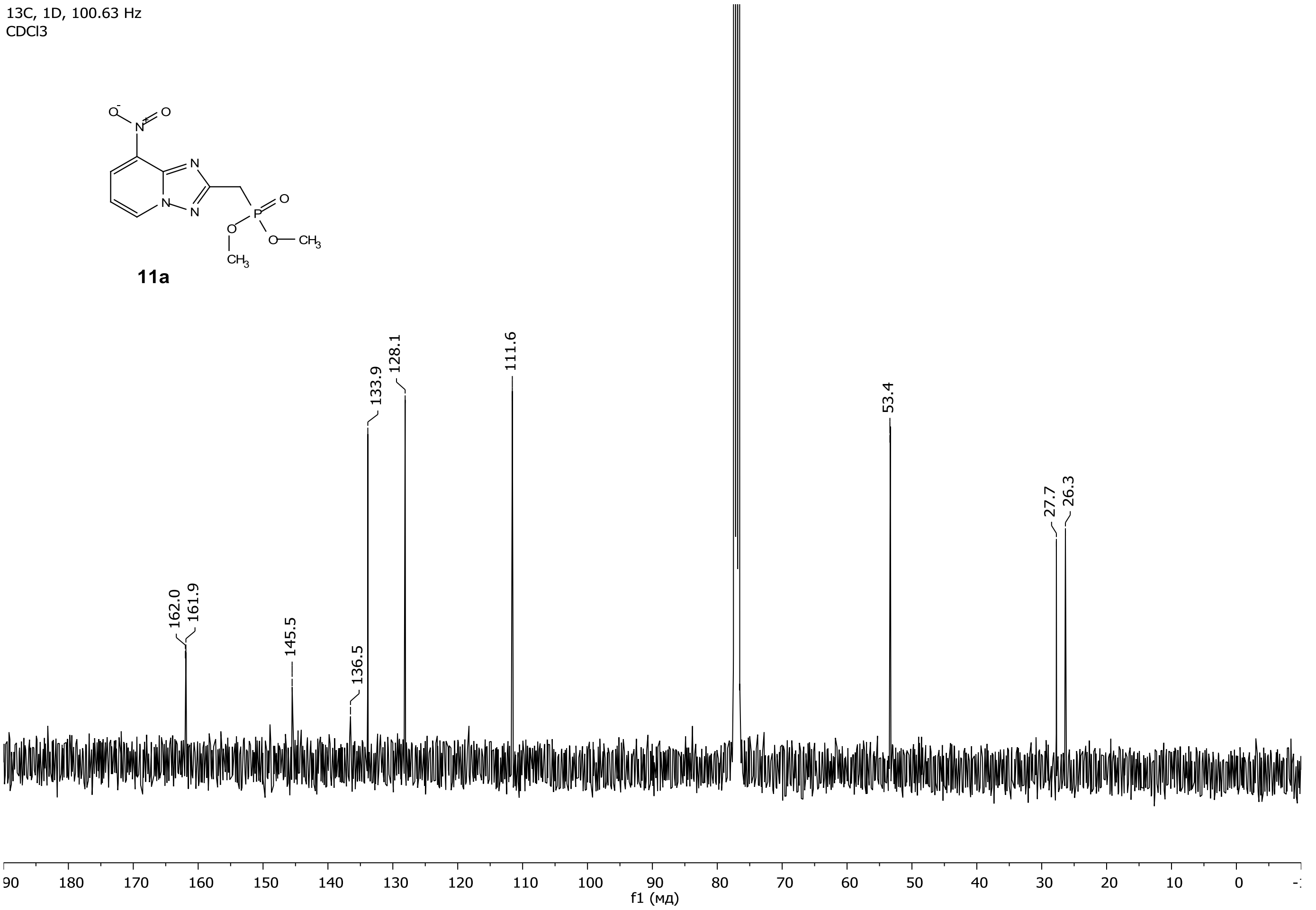
13C, 1D, 100.63 Hz
CDCl3
11a
162.0
161.9
145.5
136.5
133.9
128.1
111.6
53.4
27.7
26.3
f1 (мд)

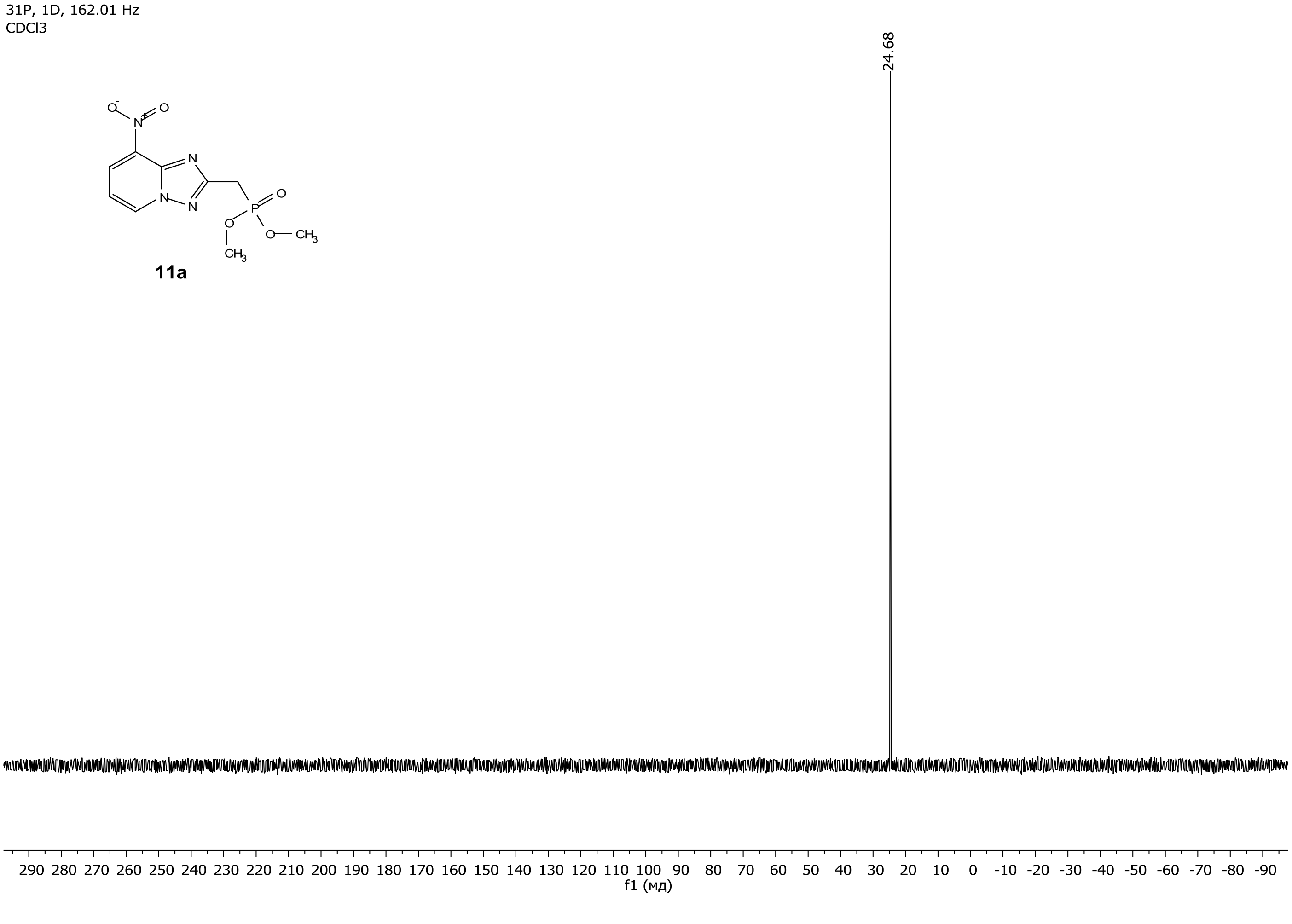
31P, 1D, 162.01 Hz
CDCl3
11a
24.68
f1 (мд)

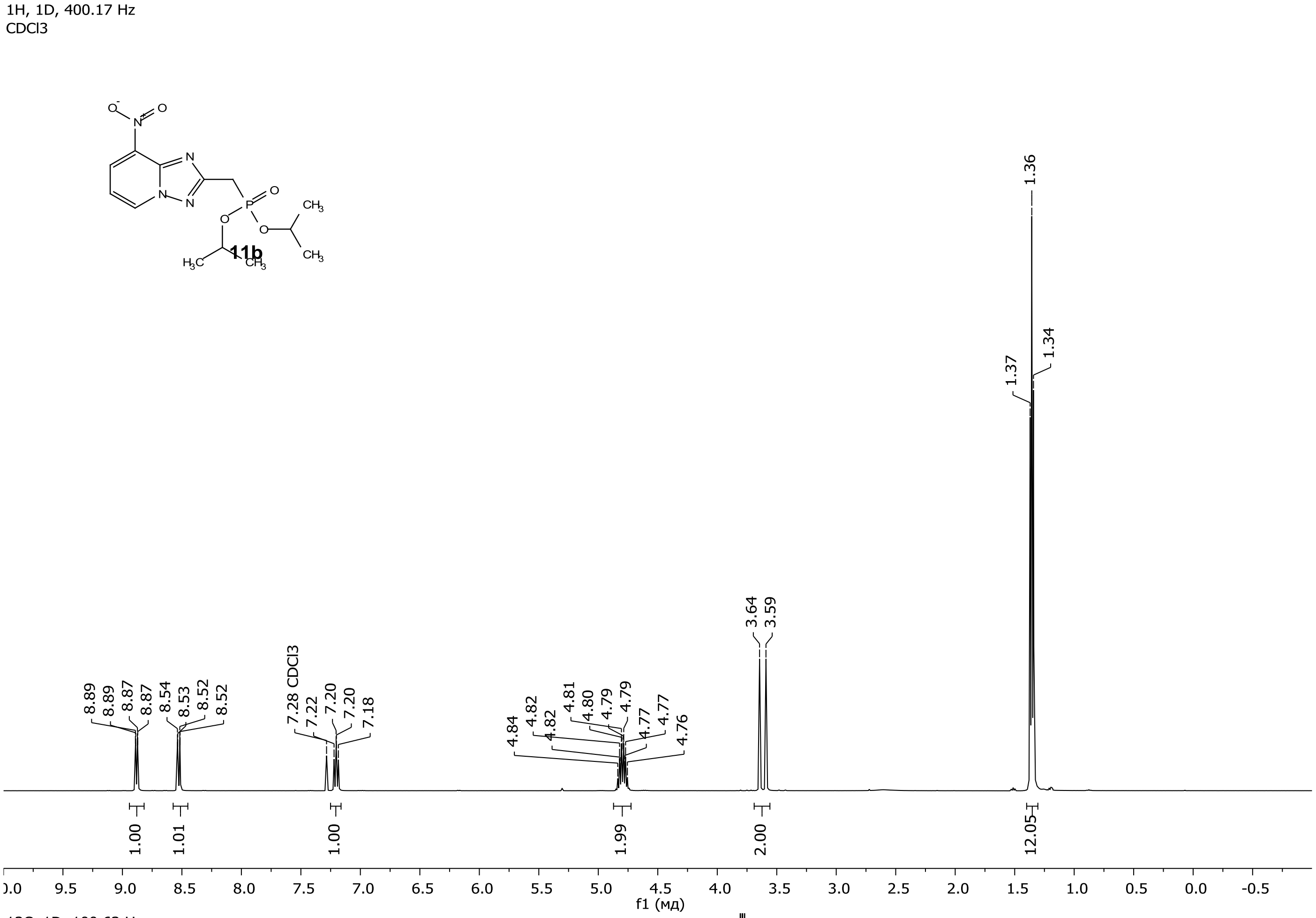
1H, 1D, 400.17 Hz
CDCl3
11b
8.89 8.89 8.87 8.87 8.54 8.53 8.52 8.52
7.28 CDCl3 7.22 7.20 7.20 7.18
4.84 4.82 4.82 4.81 4.80 4.79 4.79 4.77 4.77 4.76
3.64 3.59
1.37 1.36 1.34
1.00 1.01 1.00 1.99 2.00 12.05
f1 (мд)

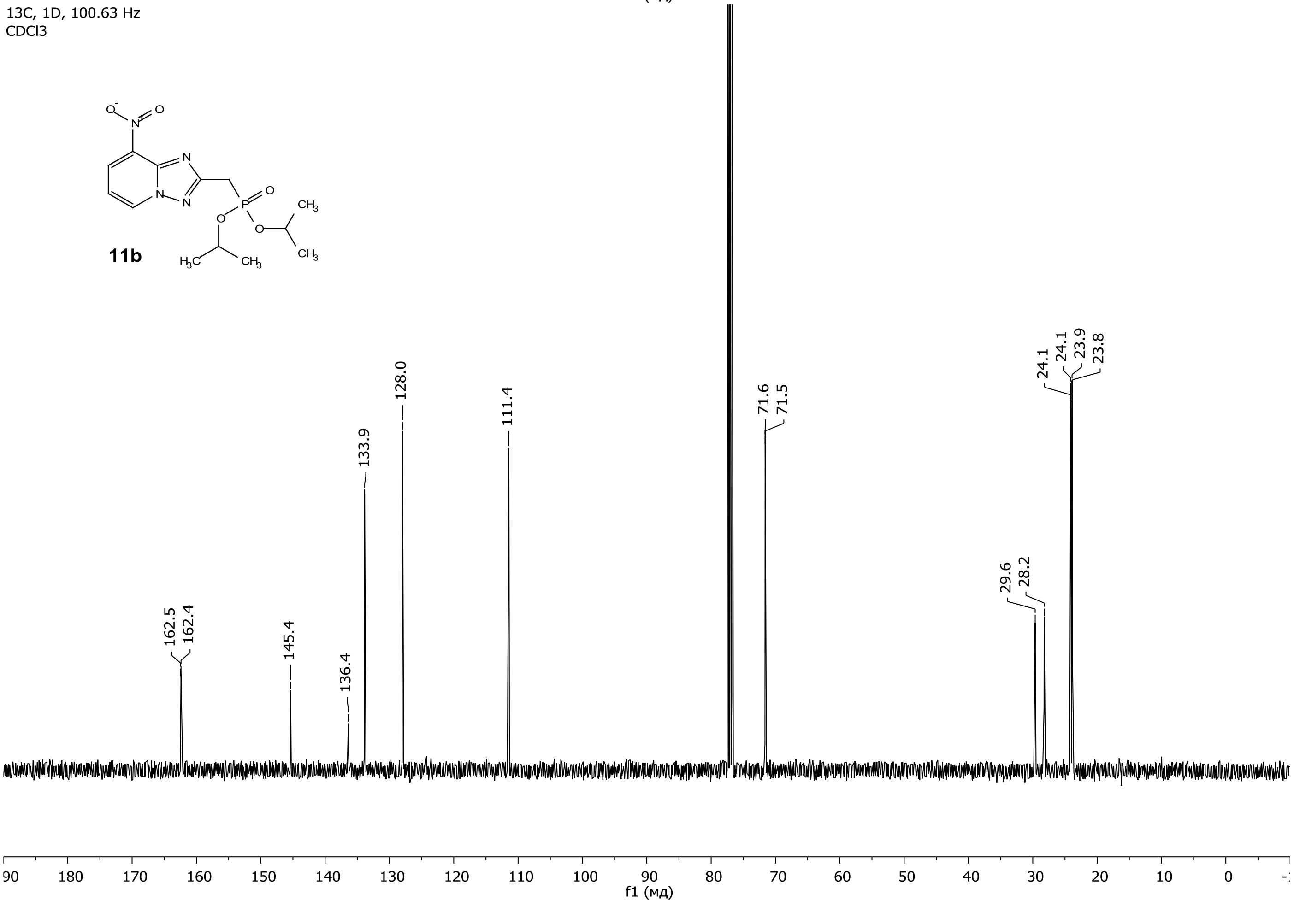
13C, 1D, 100.63 Hz
CDCl3
11b
162.5 162.4
145.4
136.4
133.9
128.0
111.4
71.6 71.5
29.6 28.2
24.1 24.1 23.9 23.8
f1 (мд)

31P, 1D, 162.01 Hz
CDCl3

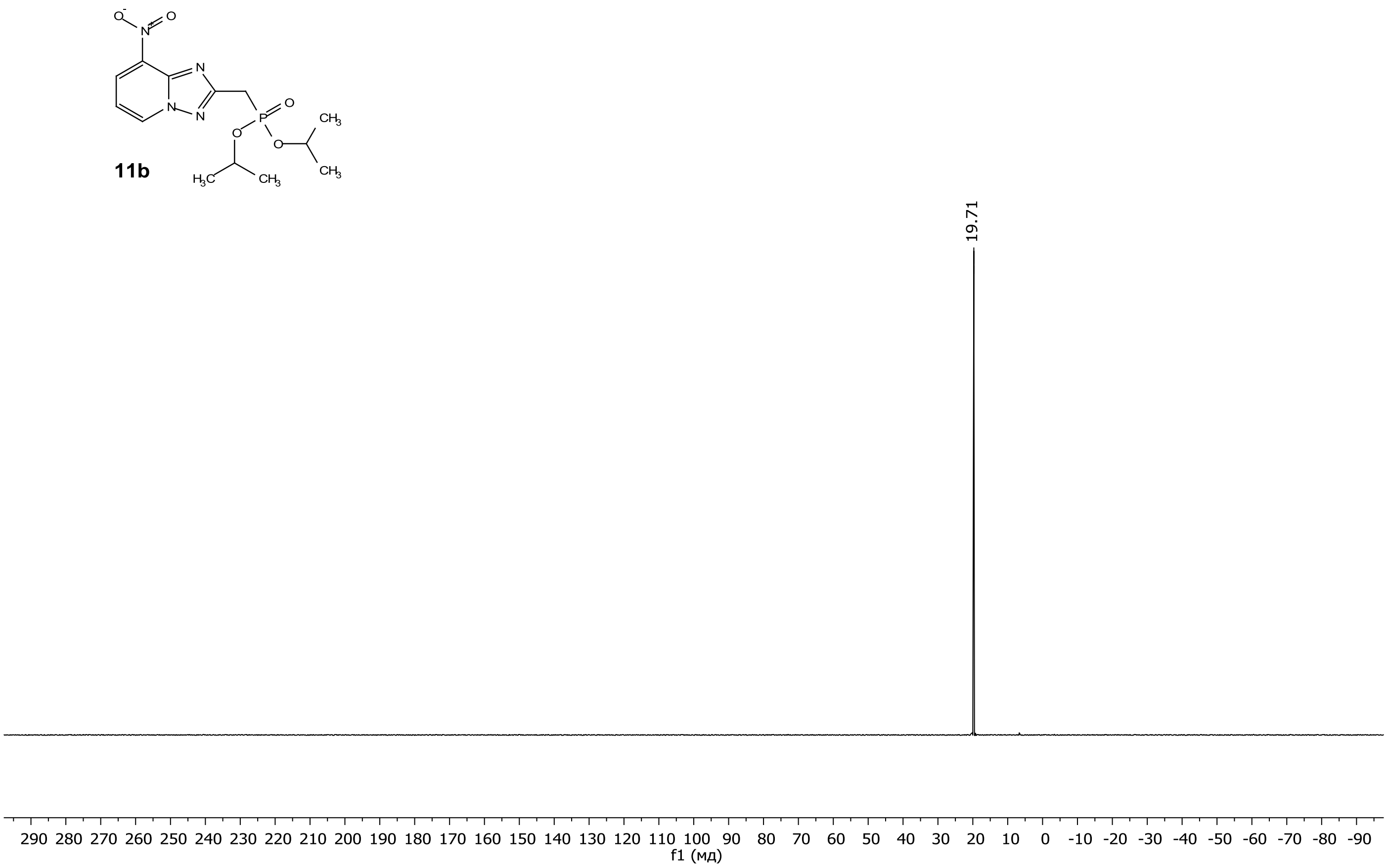

11b

1H, 1D, 400.17 Hz
CDCl3

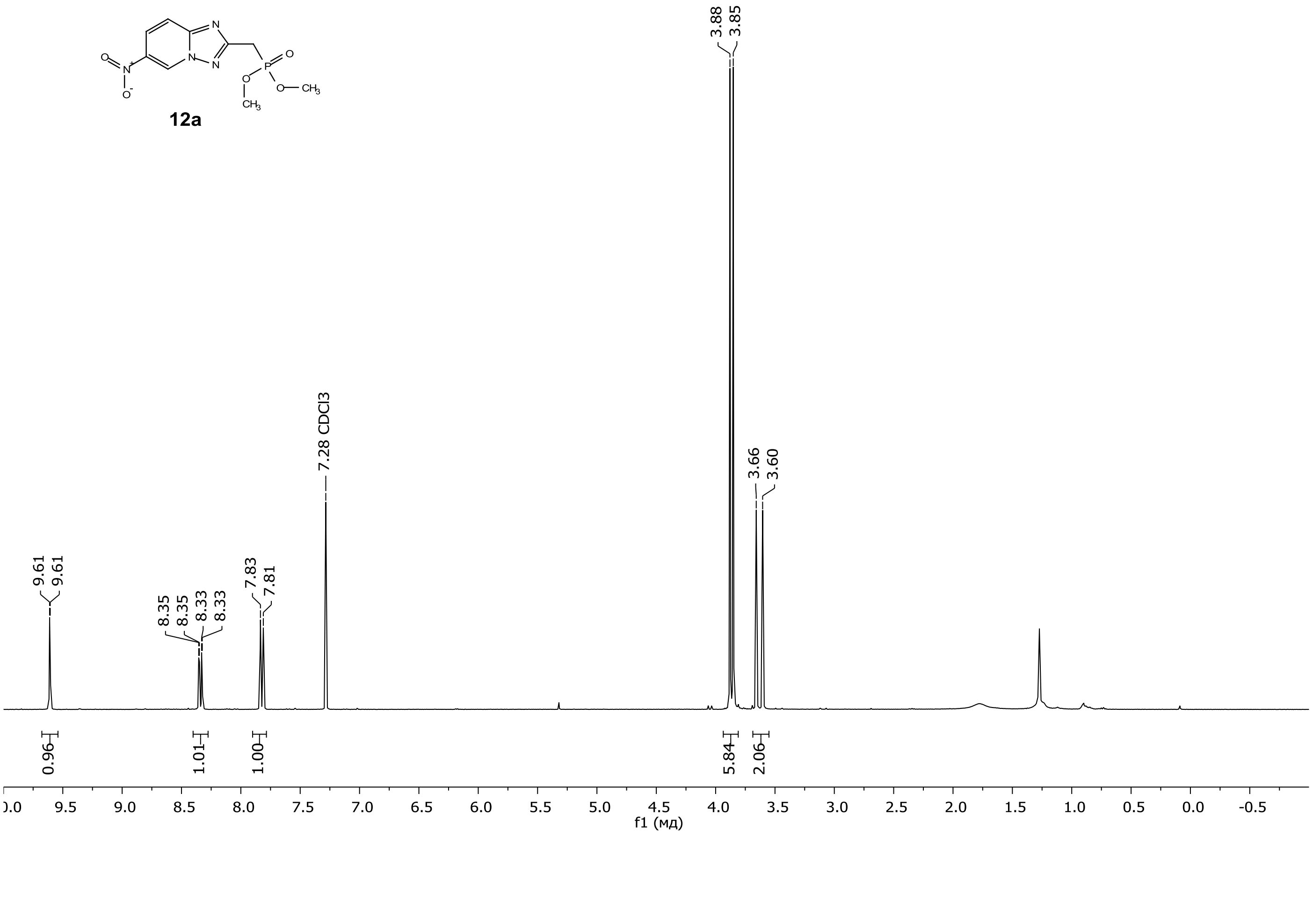

12a

13C, 1D, 100.63 Hz
CDCl3

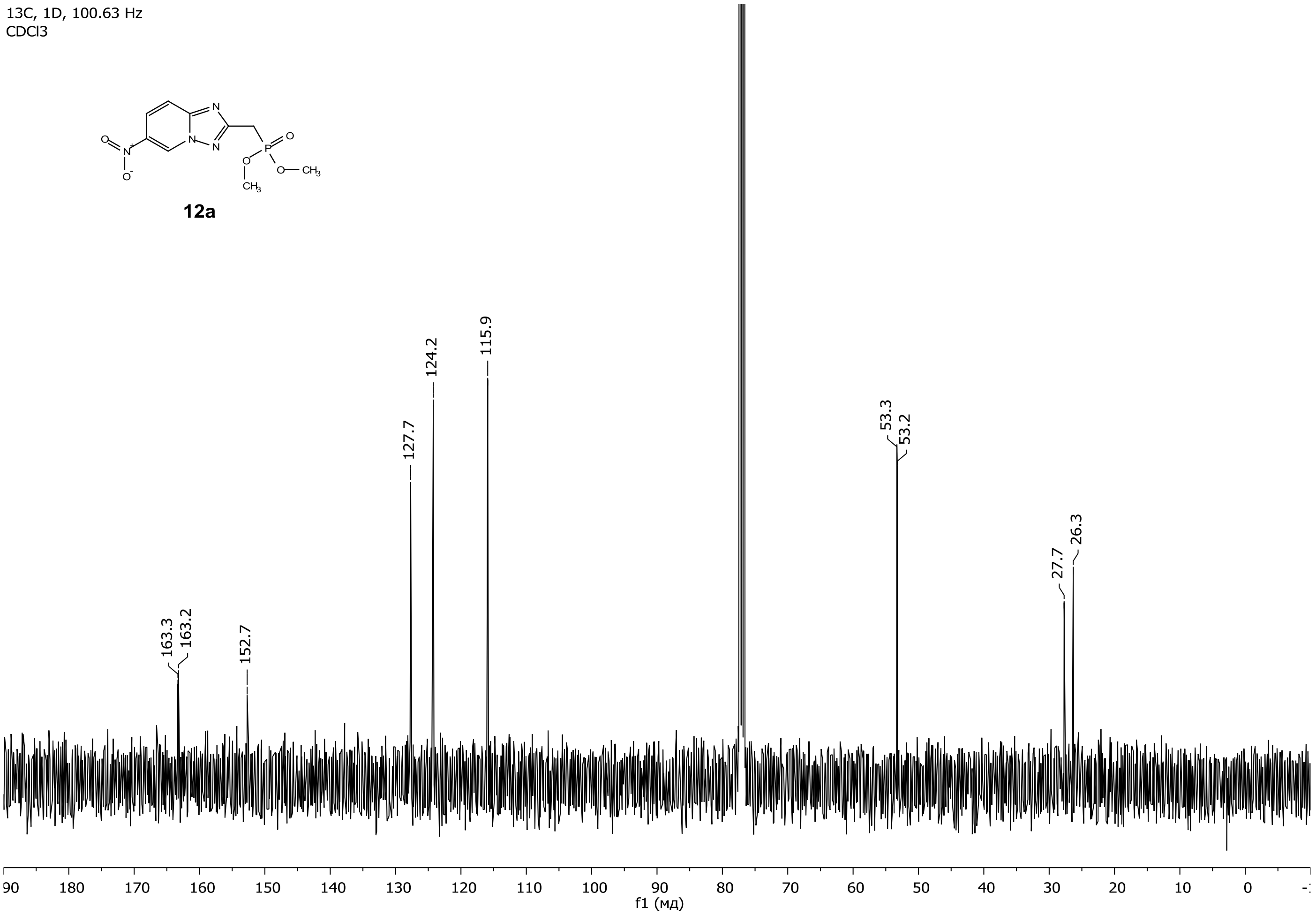

31P, 1D, 162.01 Hz
CDCl3

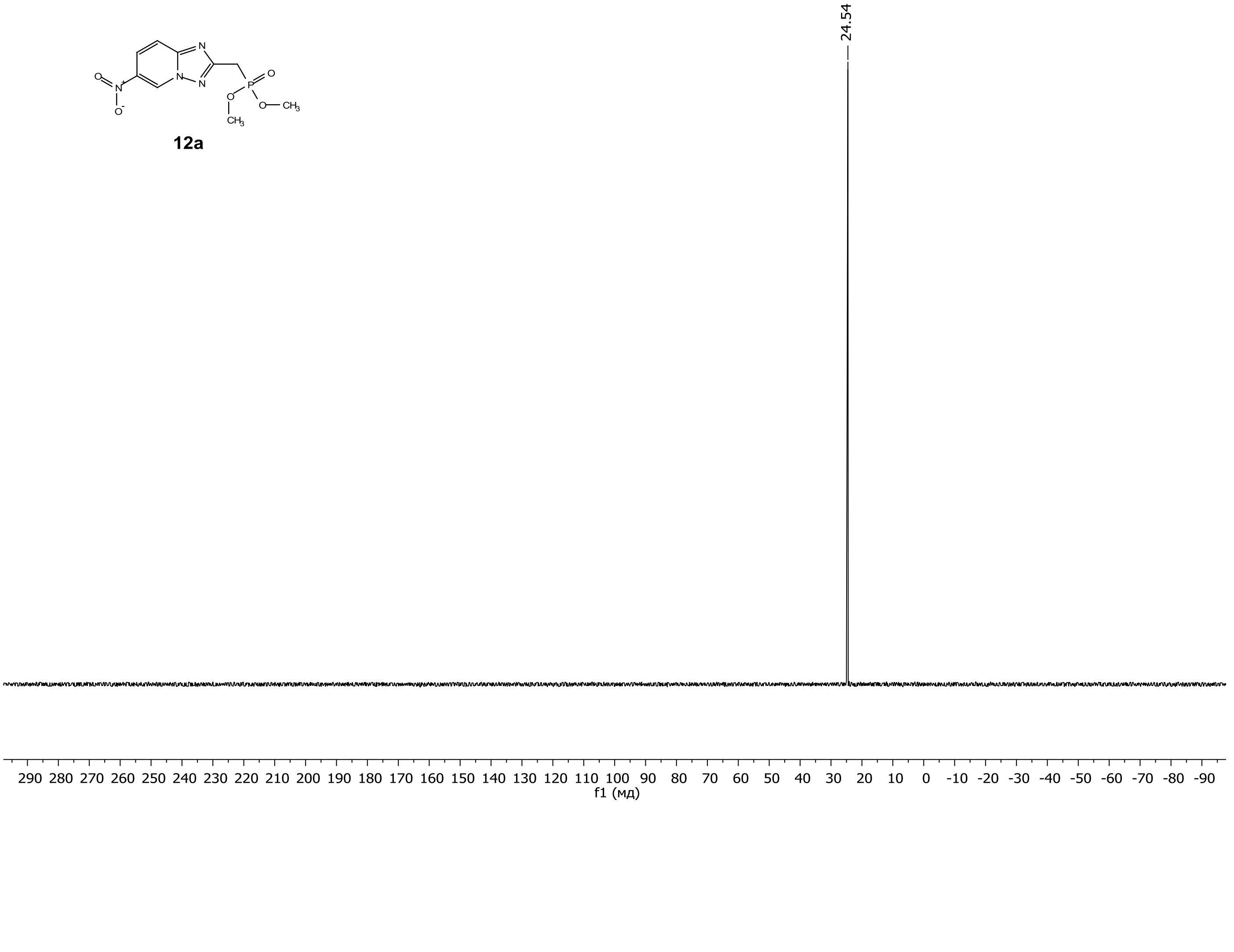

1H, 1D, 400.17 Hz
CDCl3

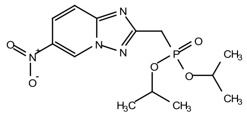

12b

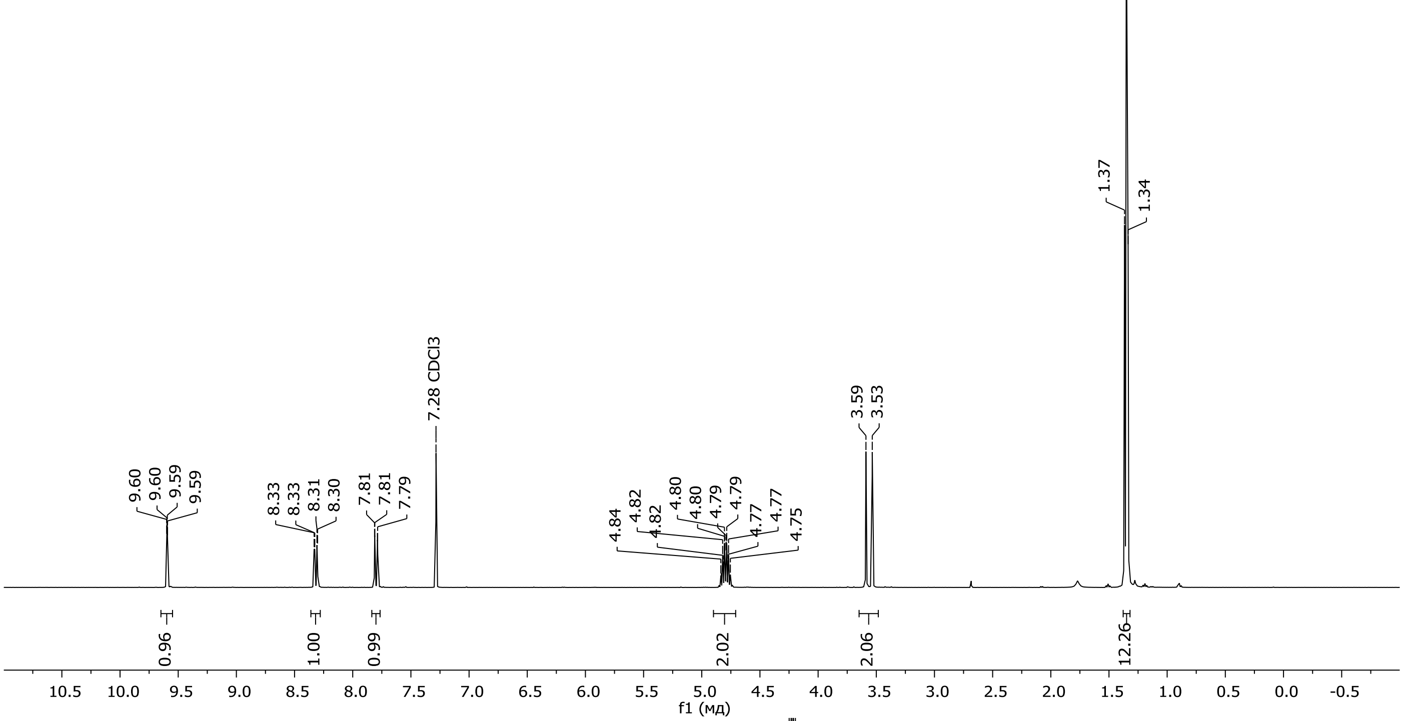

13C, 1D, 100.63 Hz
CDCl3

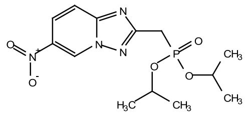

12b

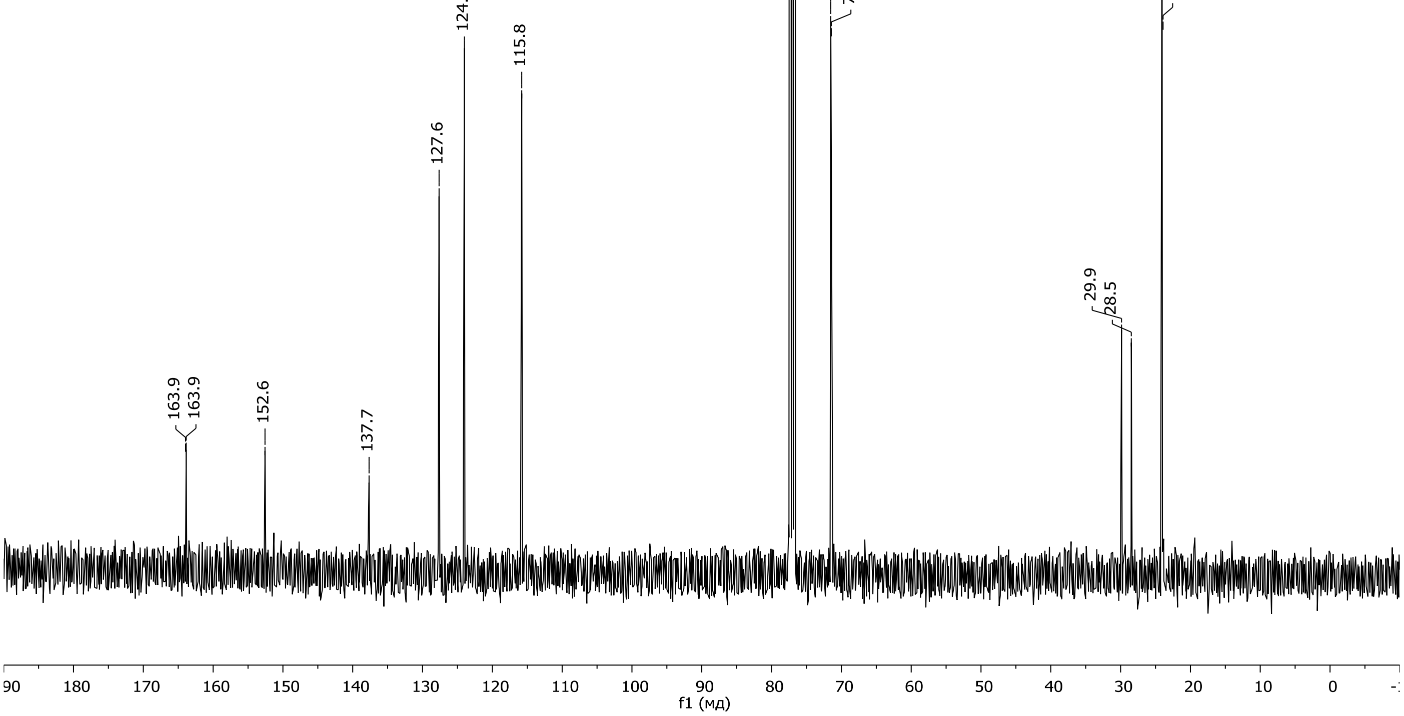

31P, 1D, 162.01 Hz
CDCl3

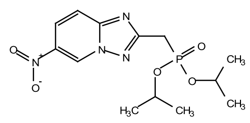

12b

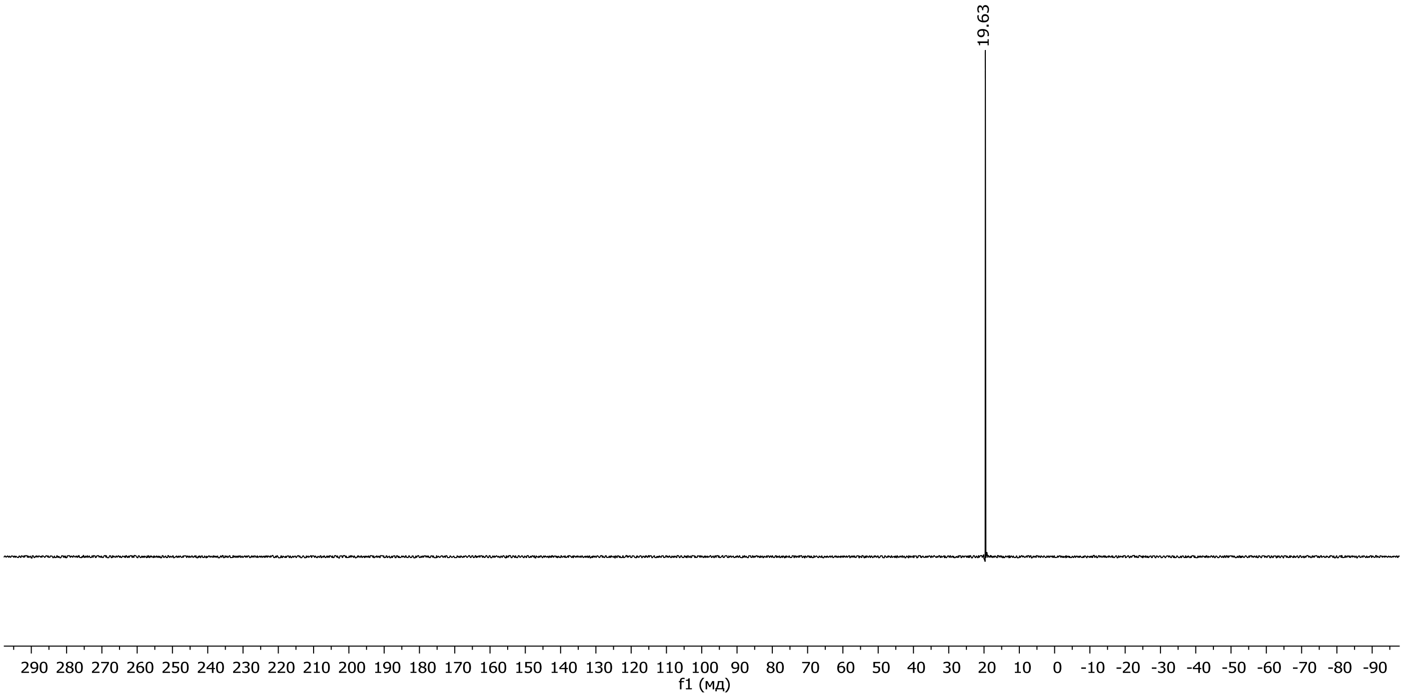

1H, 1D, 400.17 Hz
CDCl3

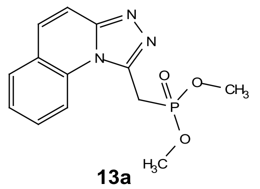

13a

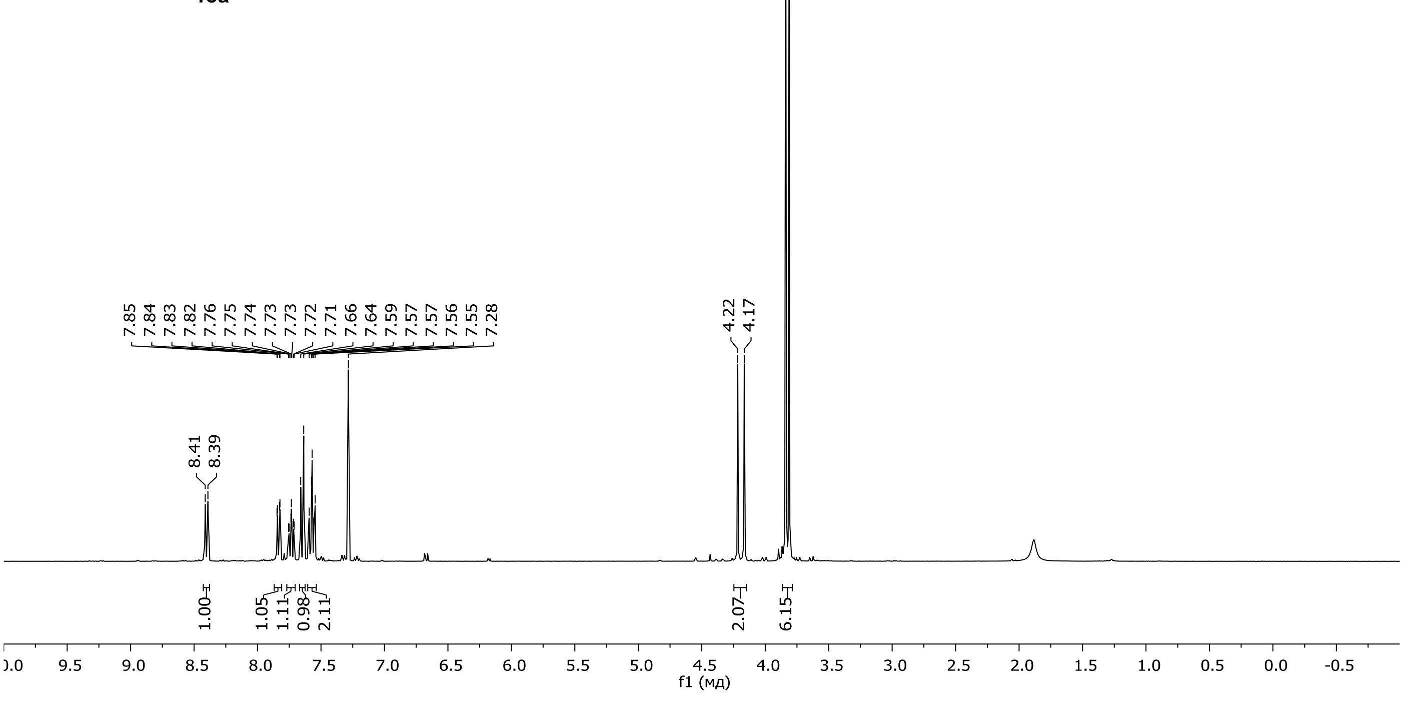

13C, 1D, 100.63 Hz
CDCl3

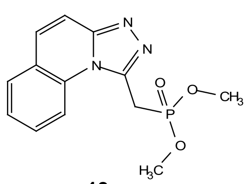

13a

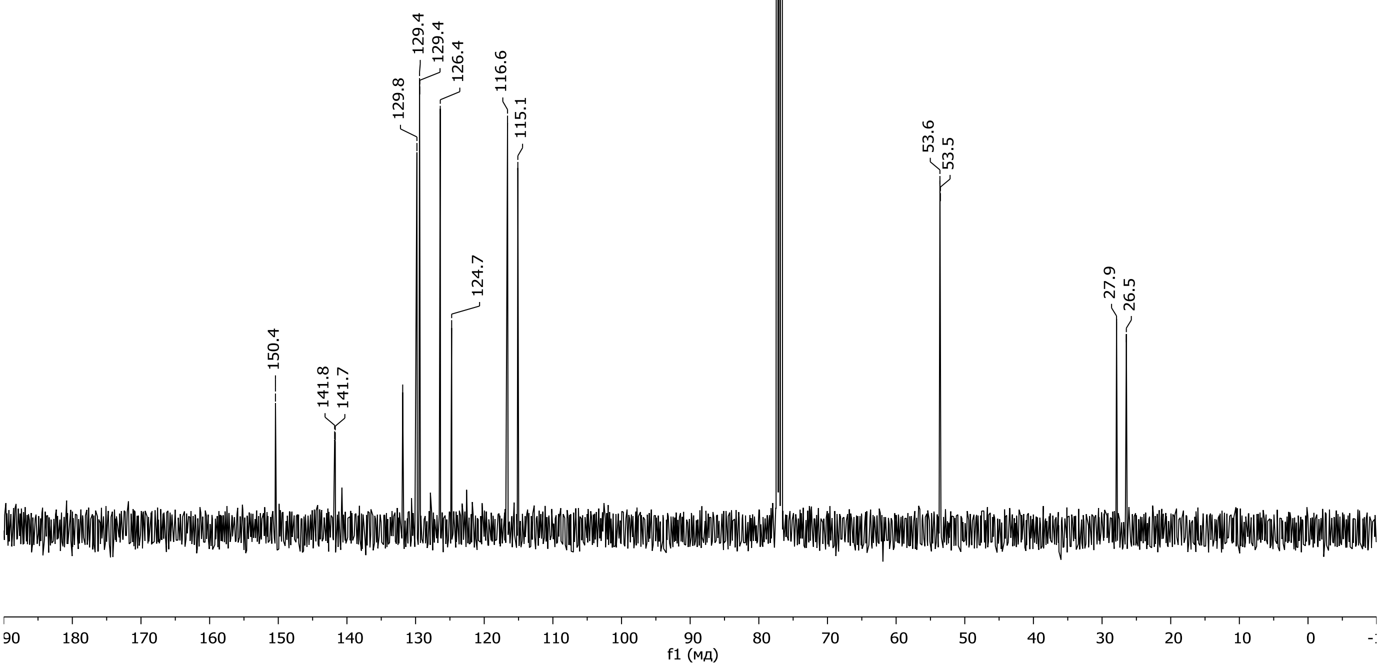

31P, 1D, 162.01 Hz
CDCl3

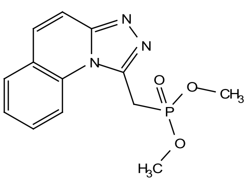

13a

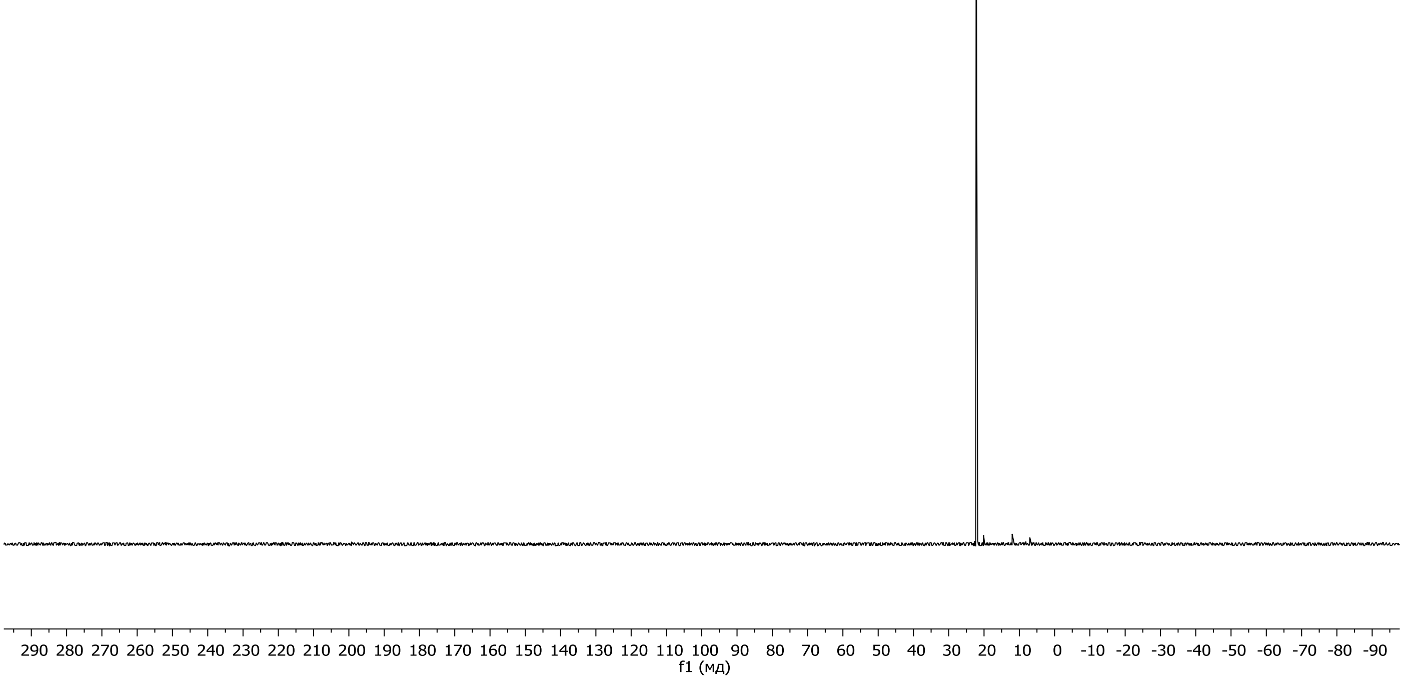

1H, 1D, 400.17 Hz
CDCl3

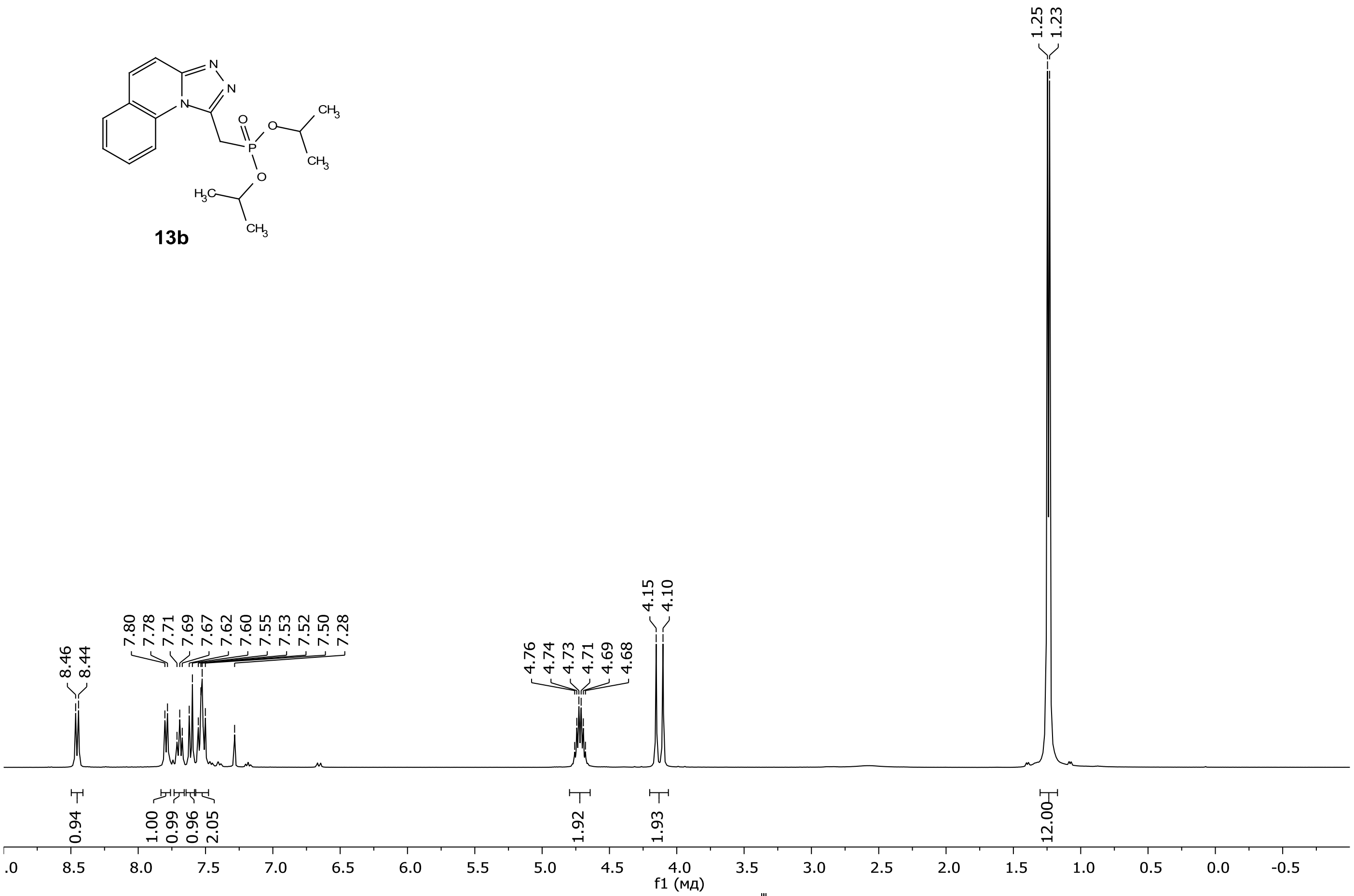

13C, 1D, 100.63 Hz
CDCl3

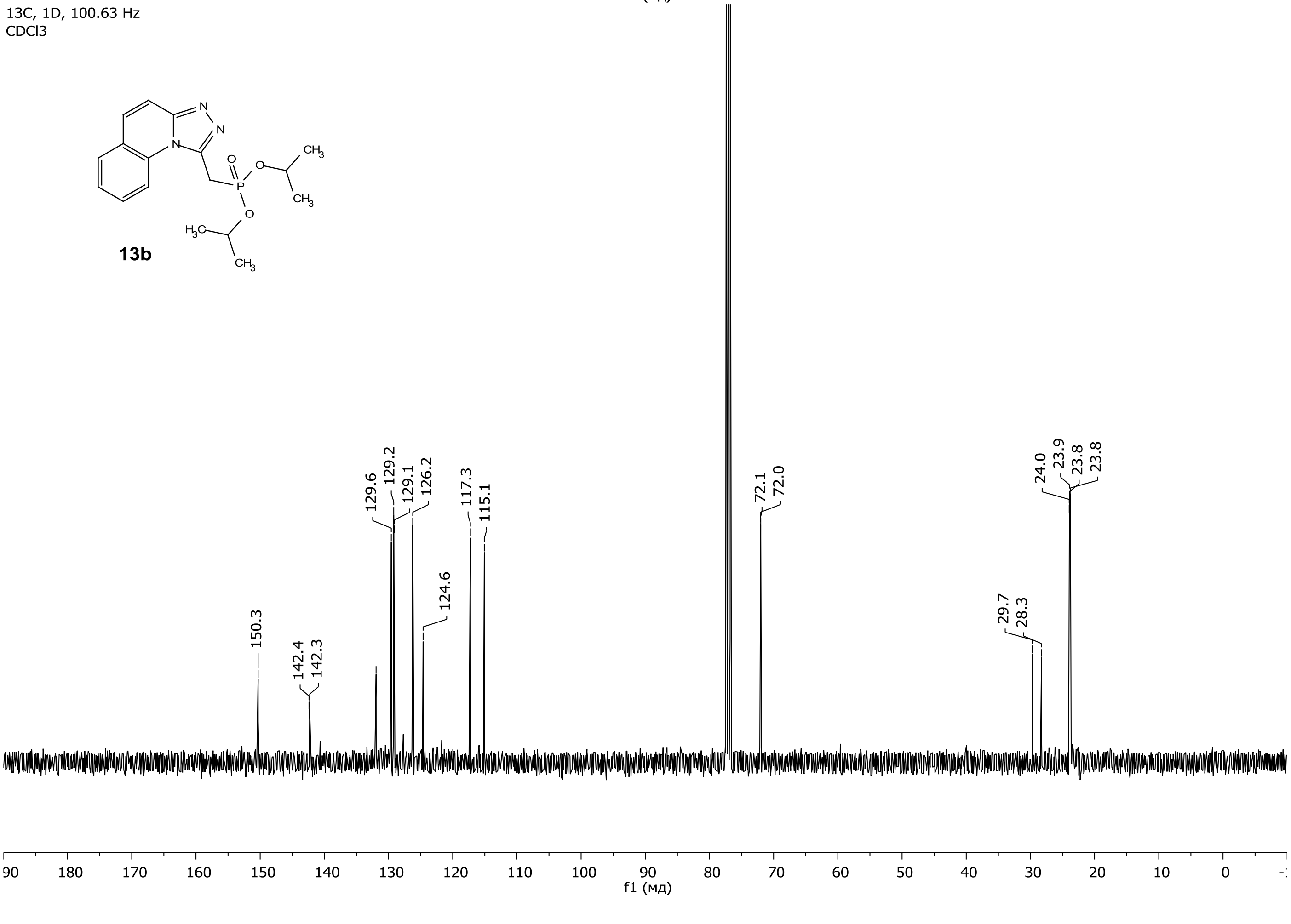

31P, 1D, 162.01 Hz
CDCl3

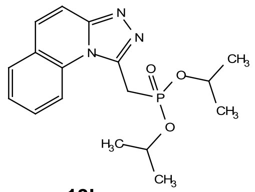

13b

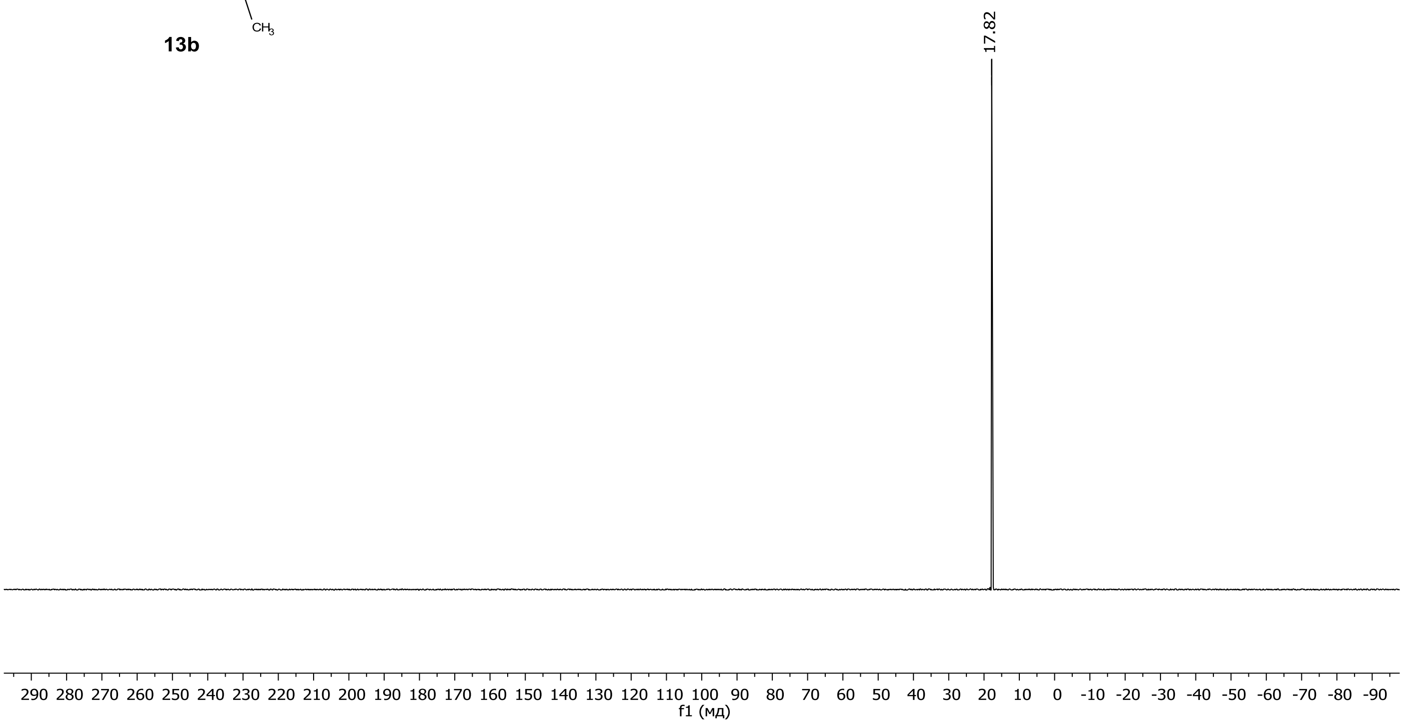

1H, 1D, 400.17 Hz
CDCl3

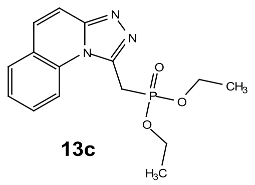

13c

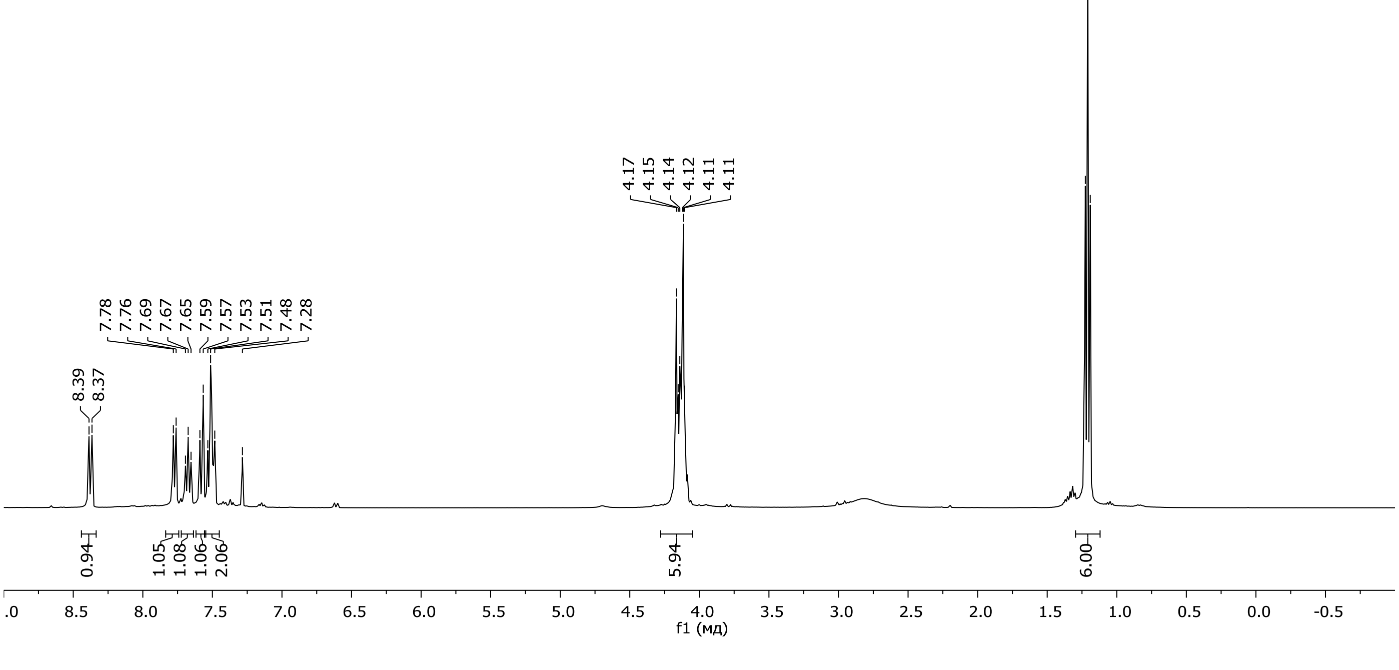

13C, 1D, 100.63 Hz
CDCl3

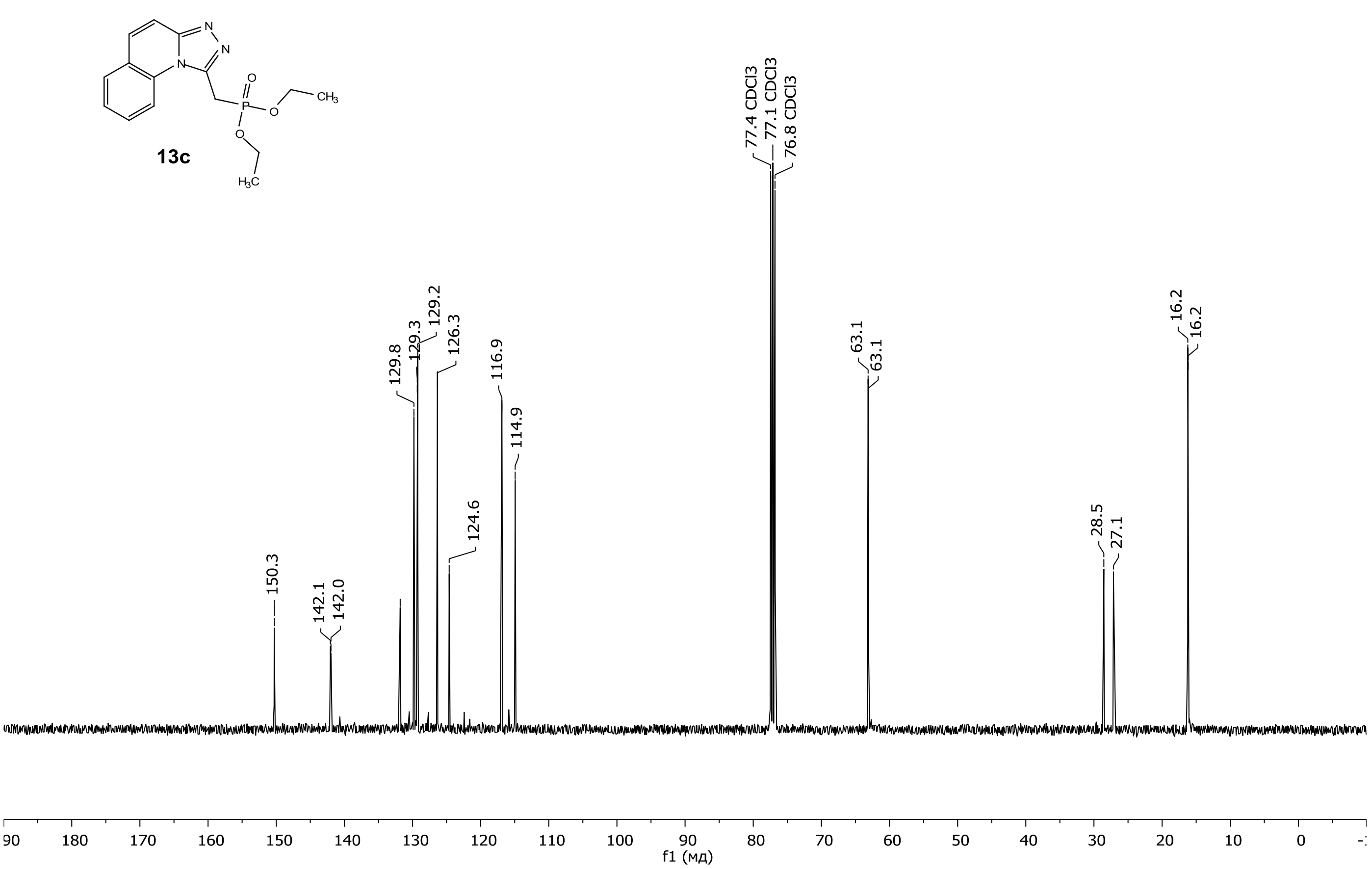

31P, 1D, 162.01 Hz
CDCl3

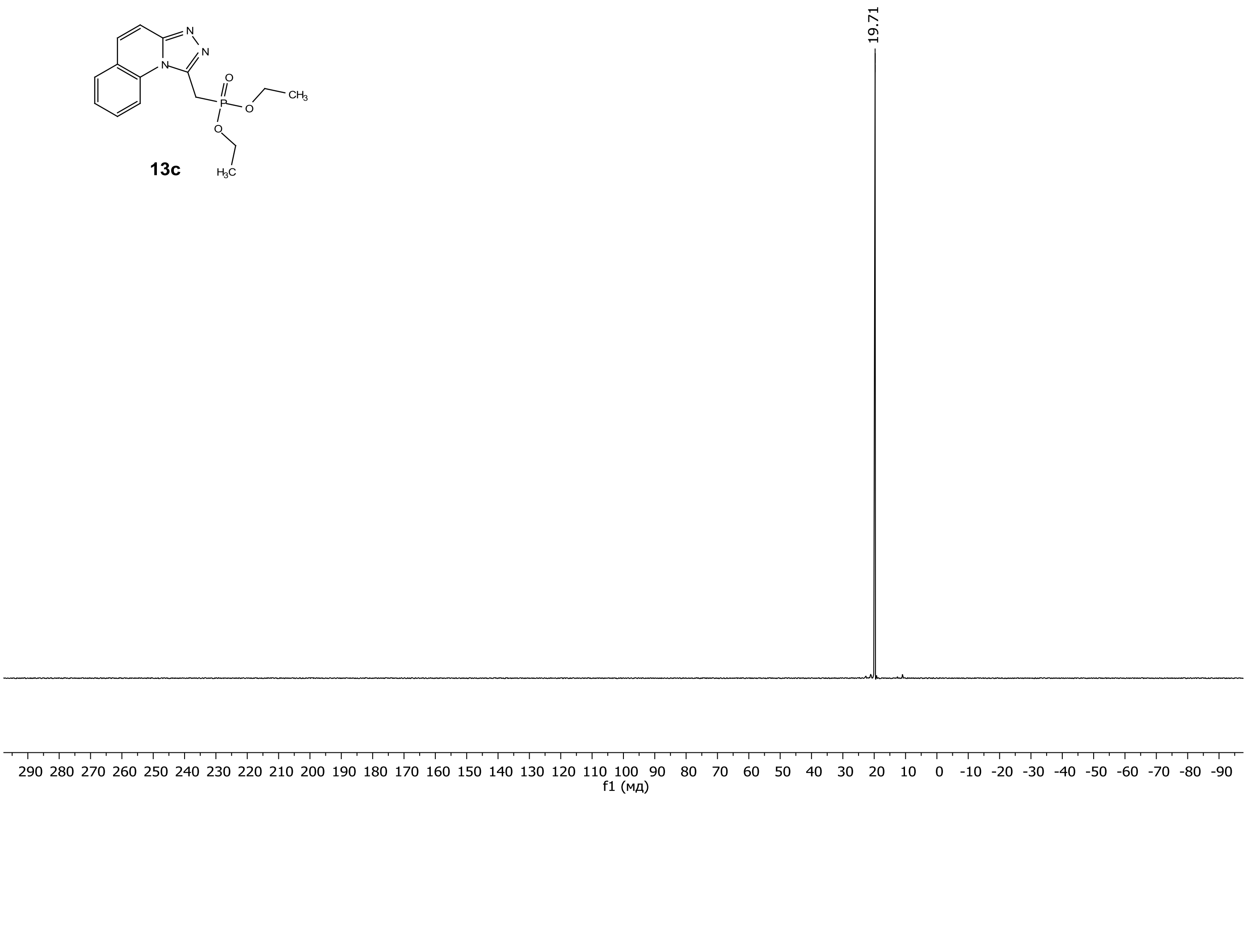

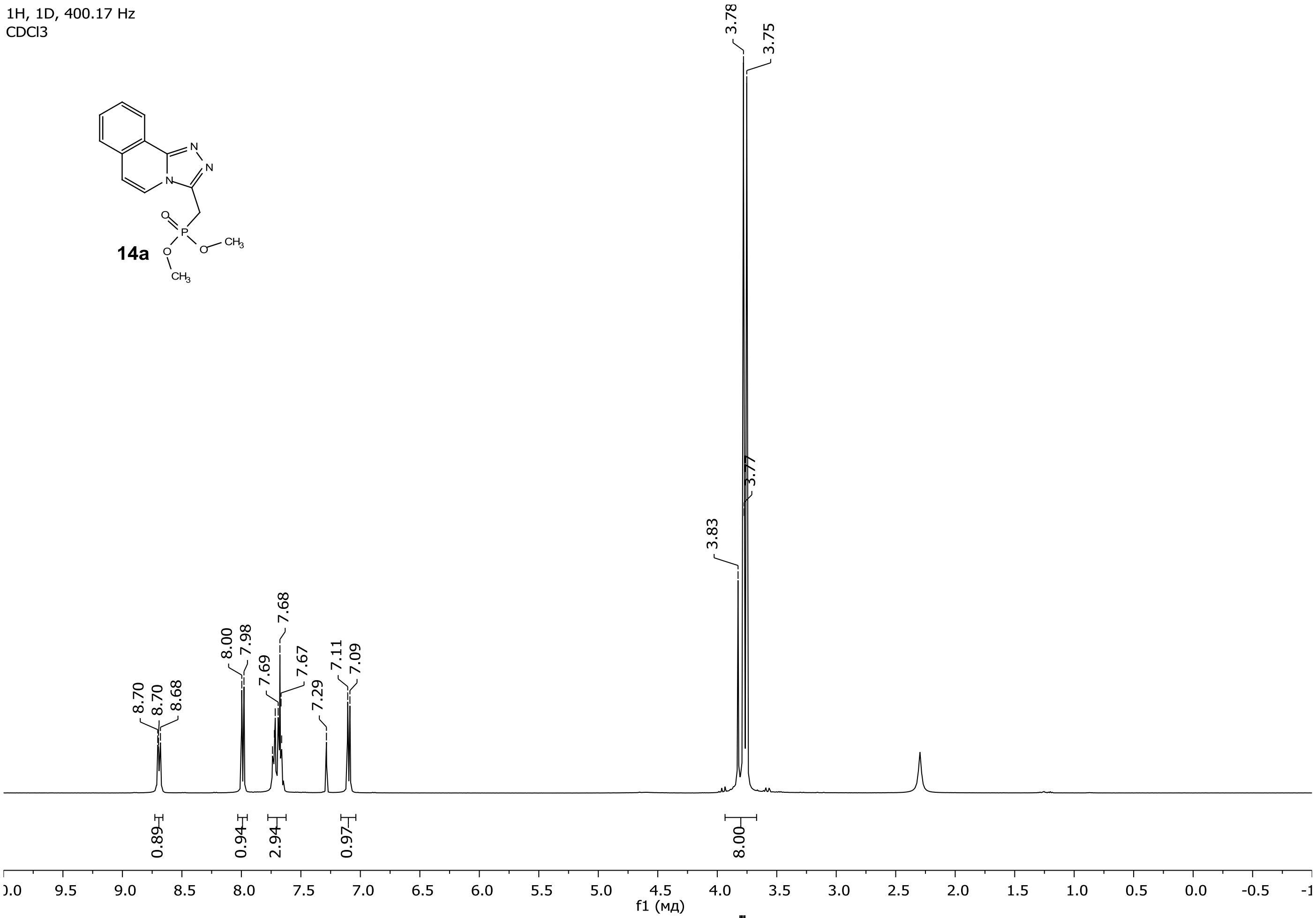

1H, 1D, 400.17 Hz
CDCl3
14a
3.78
3.75
3.77
3.83
8.70
8.70
8.68
8.00
7.98
7.69
7.68
7.67
7.29
7.11
7.09
0.89
0.94
2.94
0.97
8.00
f1 (мд)

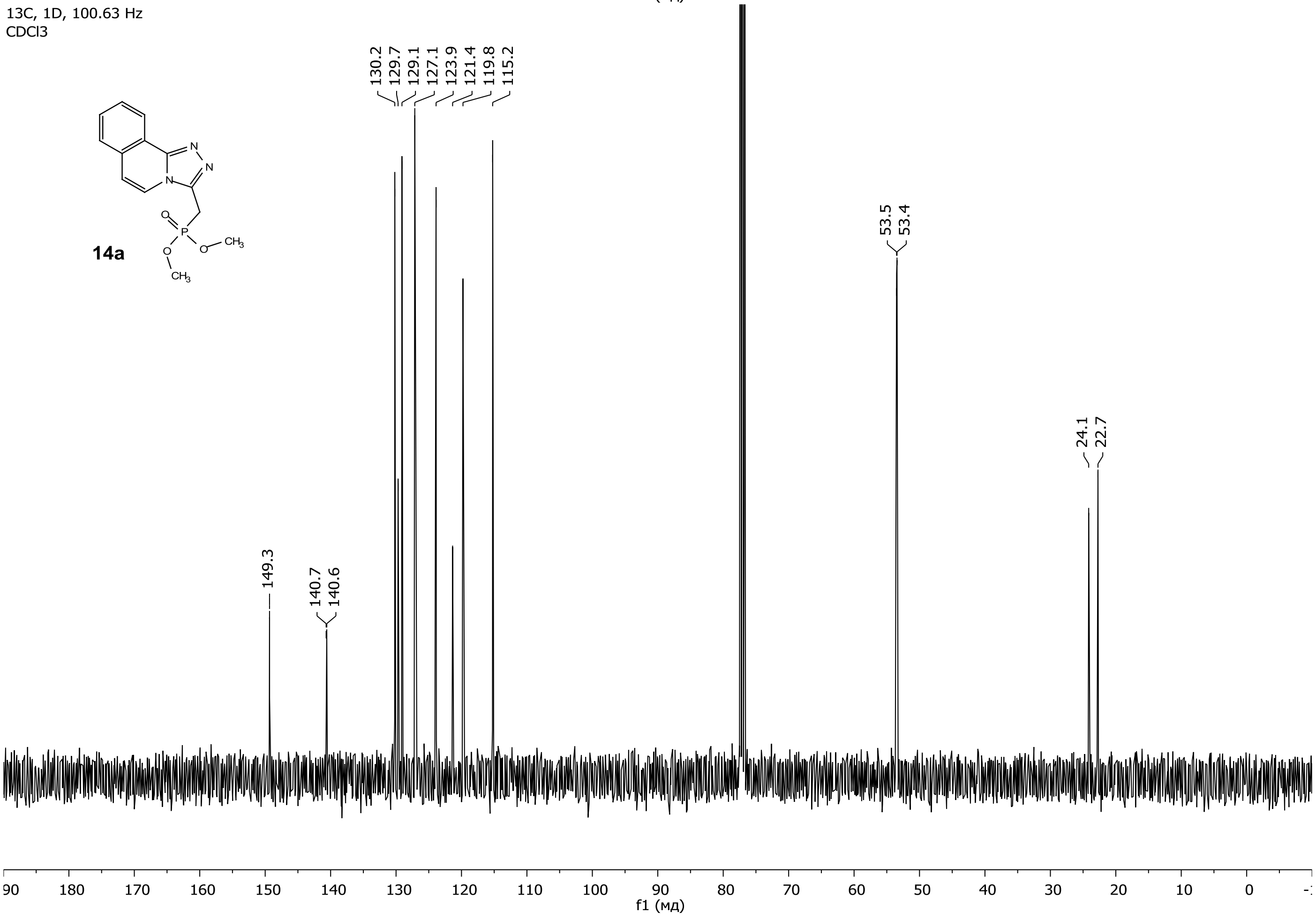

13C, 1D, 100.63 Hz
CDCl3
14a
130.2
129.7
129.1
127.1
123.9
121.4
119.8
115.2
53.5
53.4
24.1
22.7
149.3
140.7
140.6
f1 (мд)

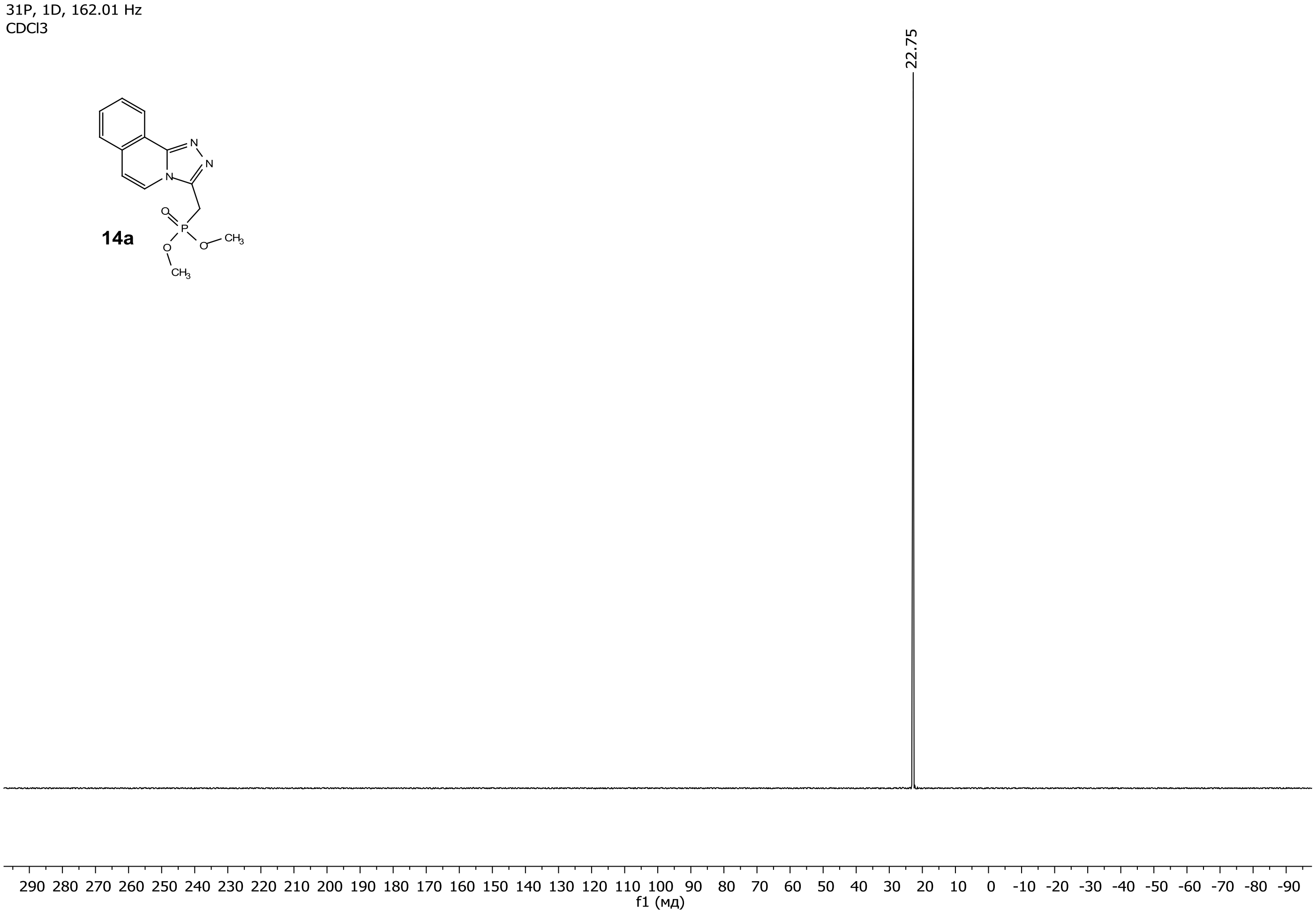
31P, 1D, 162.01 Hz
CDCl3
14a
22.75
290 280 270 260 250 240 230 220 210 200 190 180 170 160 150 140 130 120 110 100 90 80 70 60 50 40 30 20 10 0 -10 -20 -30 -40 -50 -60 -70 -80 -90
f1 (мд)

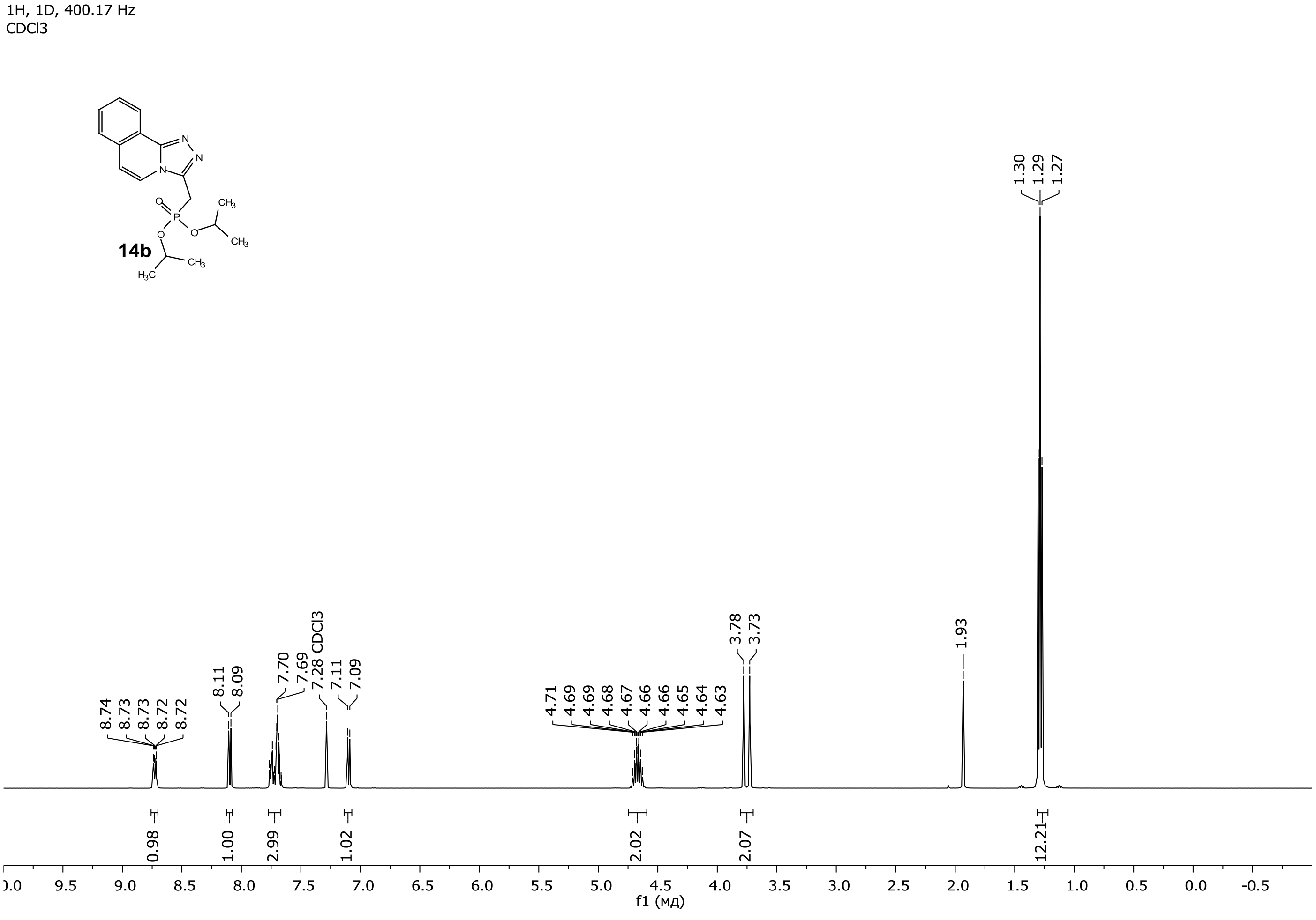
1H, 1D, 400.17 Hz
CDCl3
14b
8.74 8.73 8.73 8.72 8.72
8.11 8.09
7.70 7.69
7.28 CDCl3
7.11 7.09
4.71 4.69 4.69 4.68 4.67 4.66 4.66 4.65 4.64 4.63
3.78 3.73
1.93
1.30 1.29 1.27
0.98 1.00 2.99 1.02 2.02 2.07 12.21
9.5 9.0 8.5 8.0 7.5 7.0 6.5 6.0 5.5 5.0 4.5 4.0 3.5 3.0 2.5 2.0 1.5 1.0 0.5 0.0 -0.5
f1 (мд)

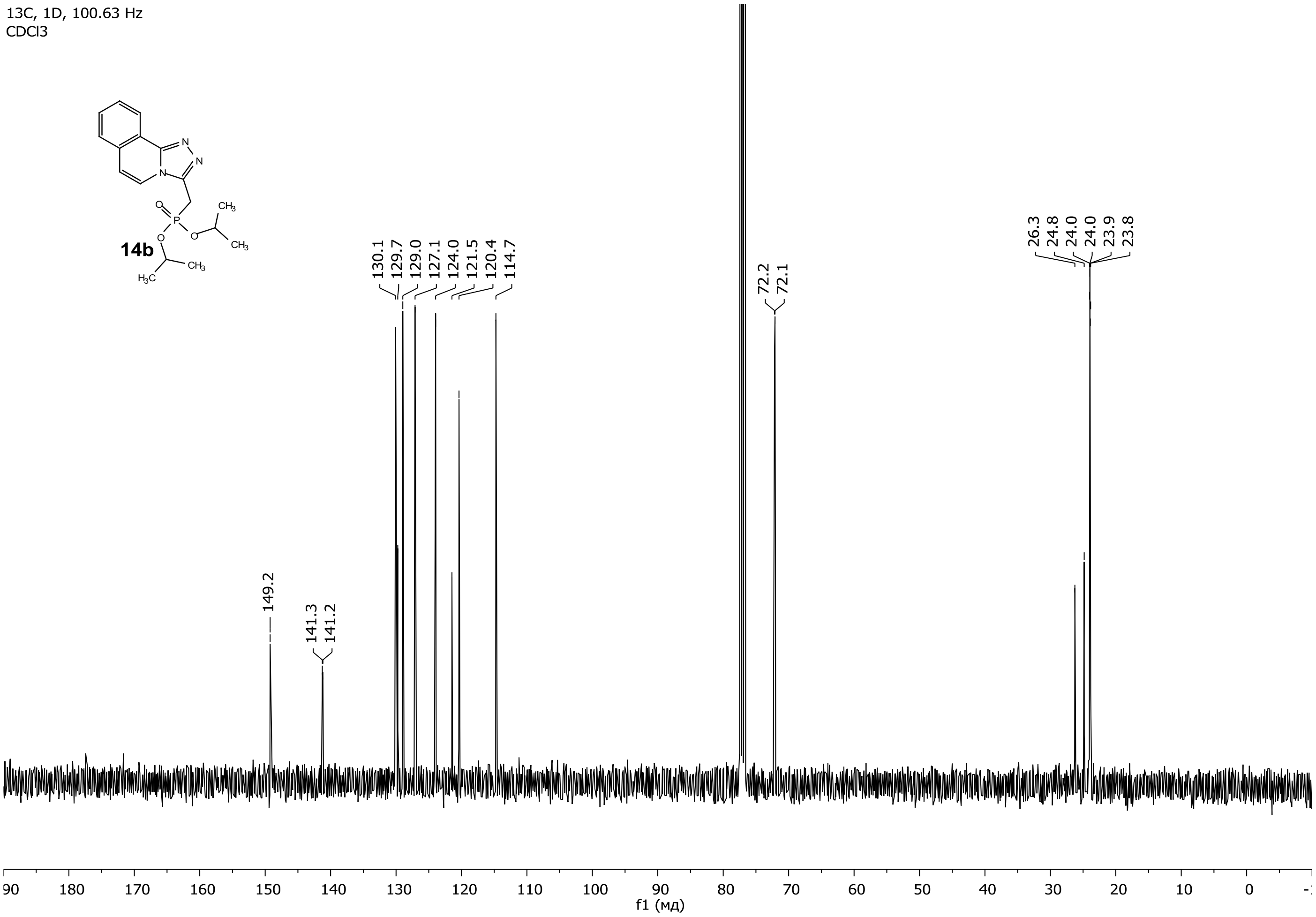
13C, 1D, 100.63 Hz
CDCl3
14b
149.2
141.3
141.2
130.1
129.7
129.0
127.1
124.0
121.5
120.4
114.7
72.2
72.1
26.3
24.8
24.0
24.0
23.9
23.8
f1 (мд)

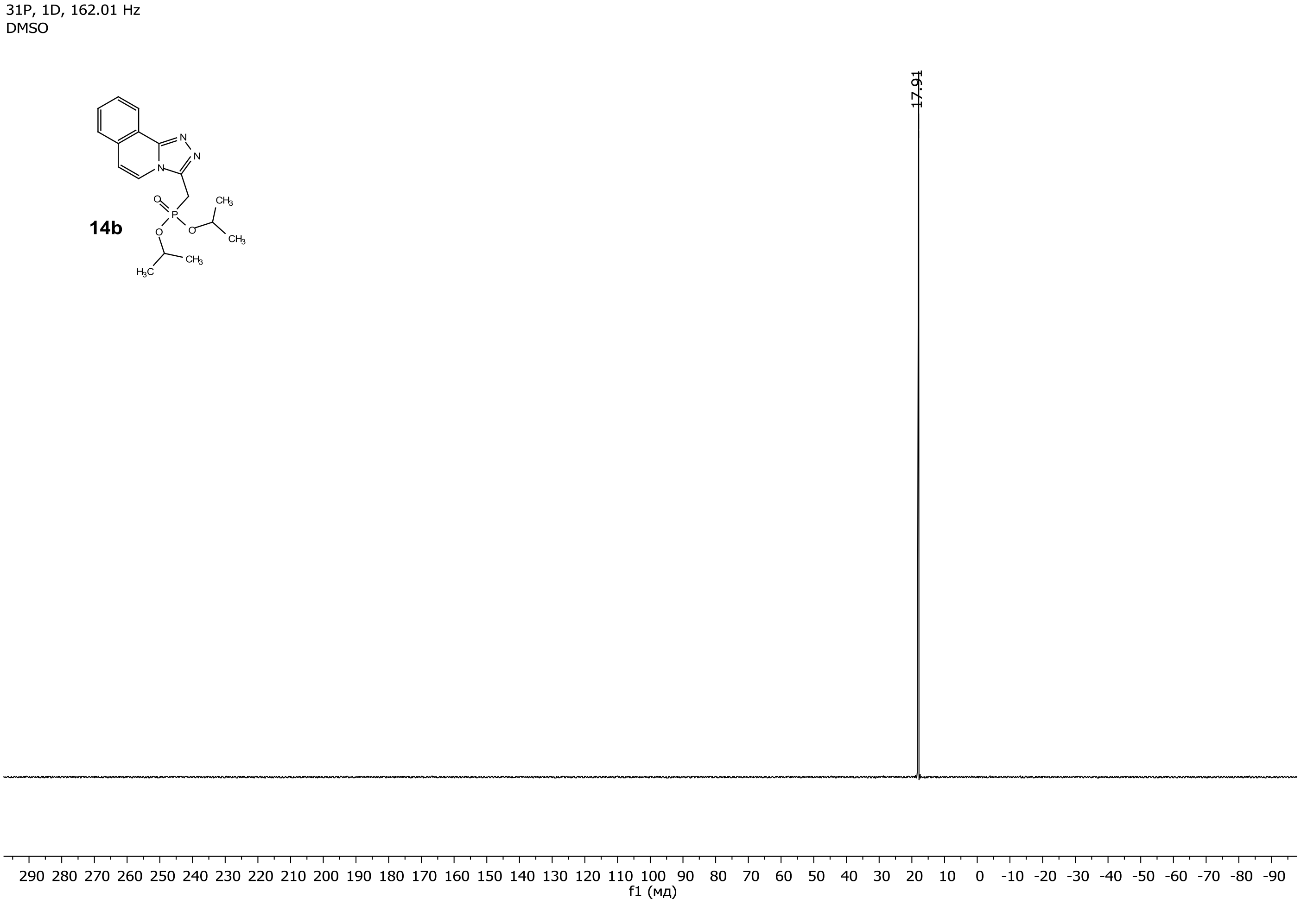
31P, 1D, 162.01 Hz
DMSO
14b
17.91
f1 (мд)

1H, 1D, 400.17 Hz
CDCl3

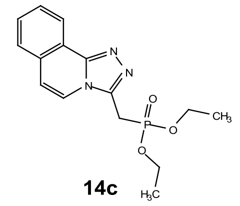

14c

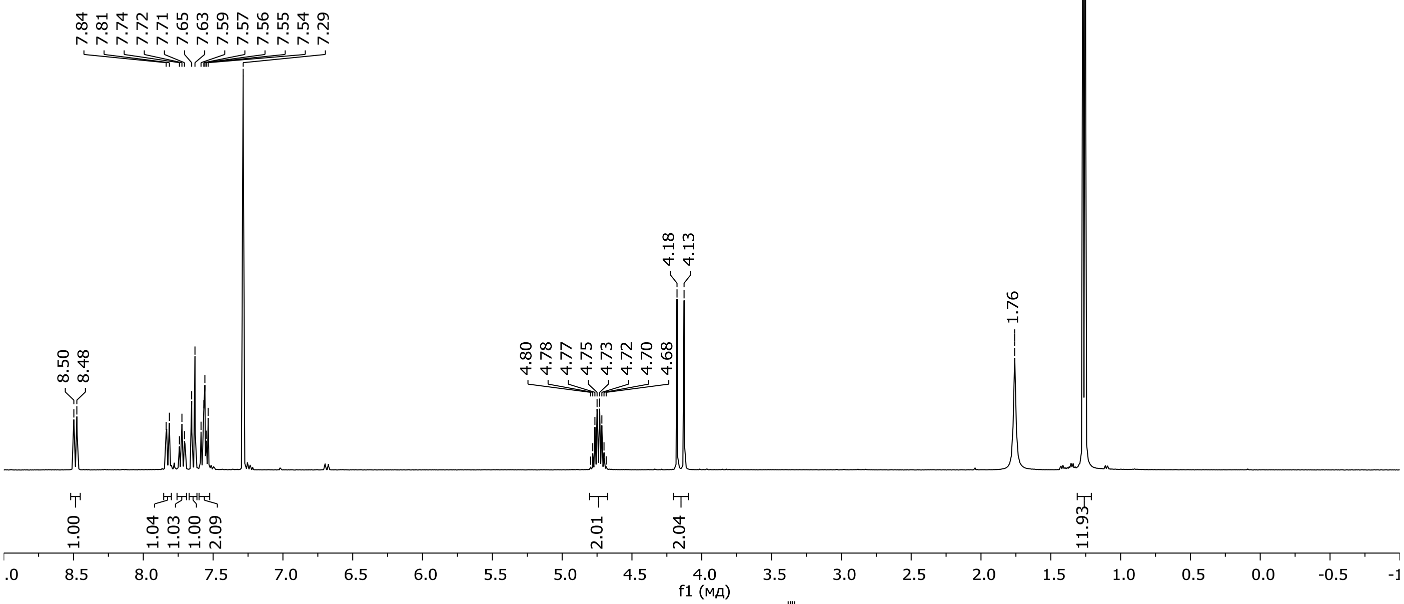

13C, 1D, 100.63 Hz
CDCl3

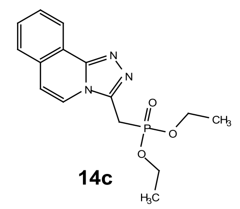

14c

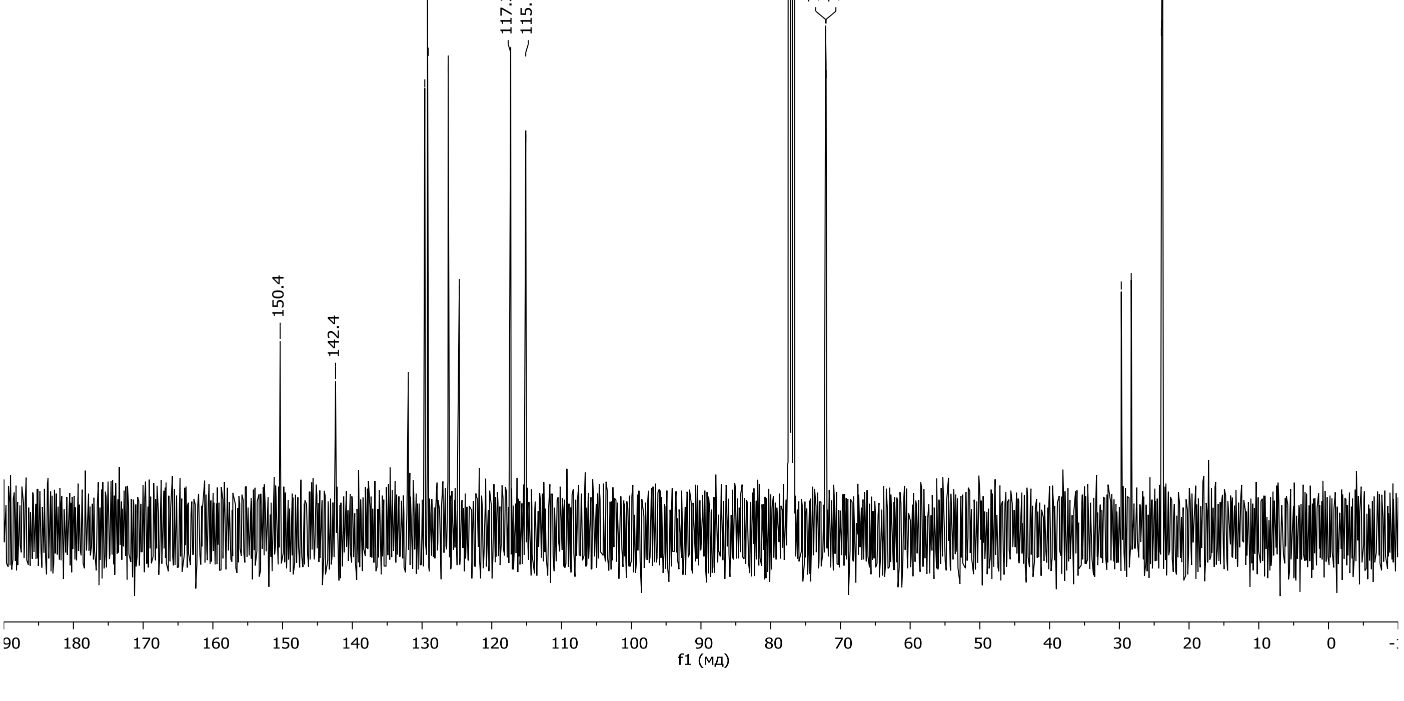

31P, 1D, 162.01 Hz
CDCl3

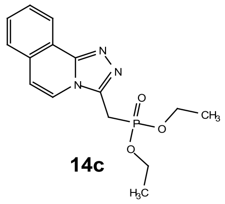

14c

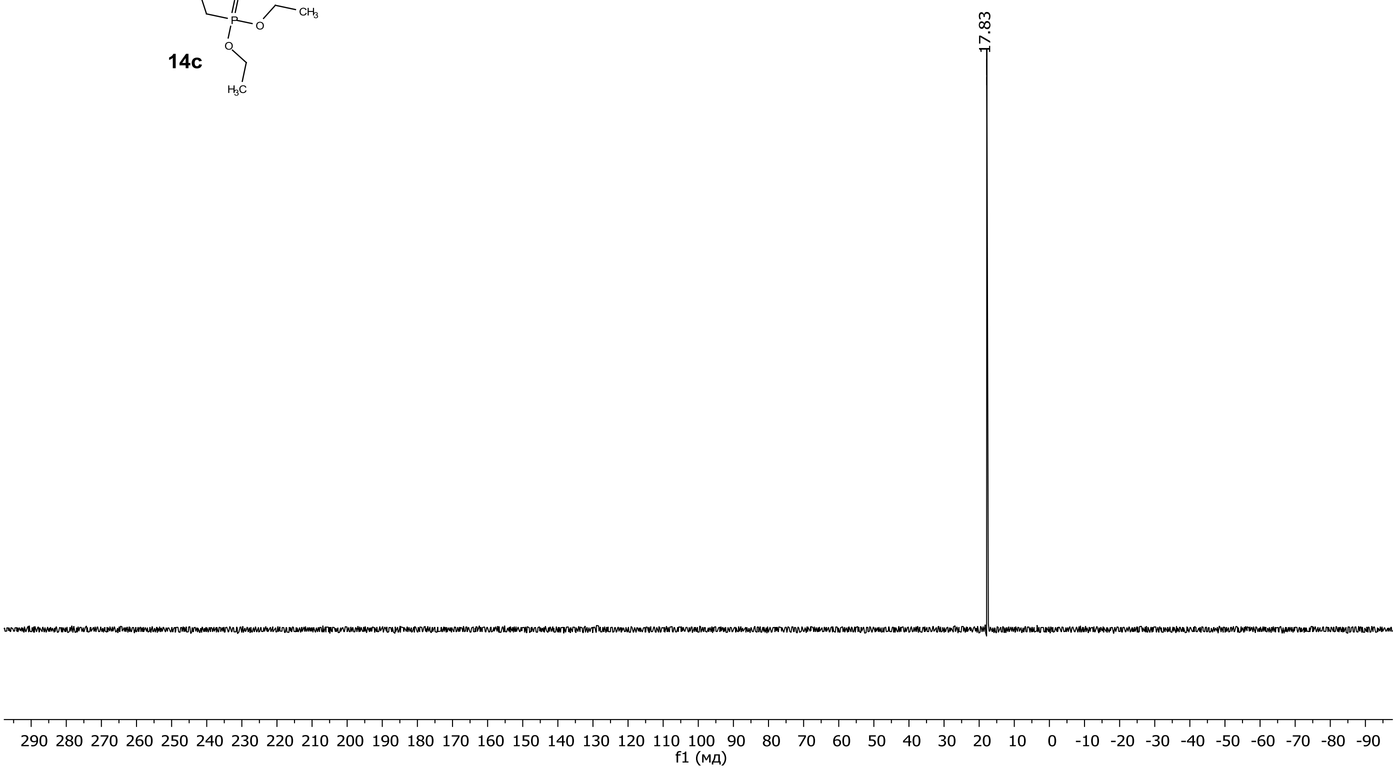

Supplement: File 1 — Experimental procedures, characterization data, and copies of 1H, 13C, and 31P NMR spectra for obtained compounds. [file Beilstein_J_Org_Chem-15-1563-s001.pdf]
